# Application of Monoferrocenylsumanenes Derived from Sonogashira Cross-Coupling or *Click Chemistry* Reactions in Highly Sensitive and Selective Cesium Cation Electrochemical Sensors

**DOI:** 10.1021/acs.joc.2c02767

**Published:** 2023-03-14

**Authors:** Artur Kasprzak, Aleksandra Gajda-Walczak, Agata Kowalczyk, Barbara Wagner, Anna M. Nowicka, Mikey Nishimoto, Mariola Koszytkowska-Stawińska, Hidehiro Sakurai

**Affiliations:** † Faculty of Chemistry, Warsaw University of Technology, Noakowskiego Str. 3, 00-664 Warsaw, Poland; ‡ Faculty of Chemistry, University of Warsaw, Pasteura Str. 1, 02-093 Warsaw, Poland; § Biological and Chemical Research Centre, Faculty of Chemistry, University of Warsaw, Zwirki i Wigury Str. 101, PL-02-093 Warsaw, Poland; ∥ Division of Applied Chemistry, Graduate School of Engineering, Osaka University, 2-1 Yamadaoka, Suita 565-0871, Osaka, Japan; ⊥ Innovative Catalysis Science Division, Institute for Open and Transdisciplinary Research Initiatives (ICS-OTRI), Osaka University, Suita 565-0871, Osaka, Japan

## Abstract

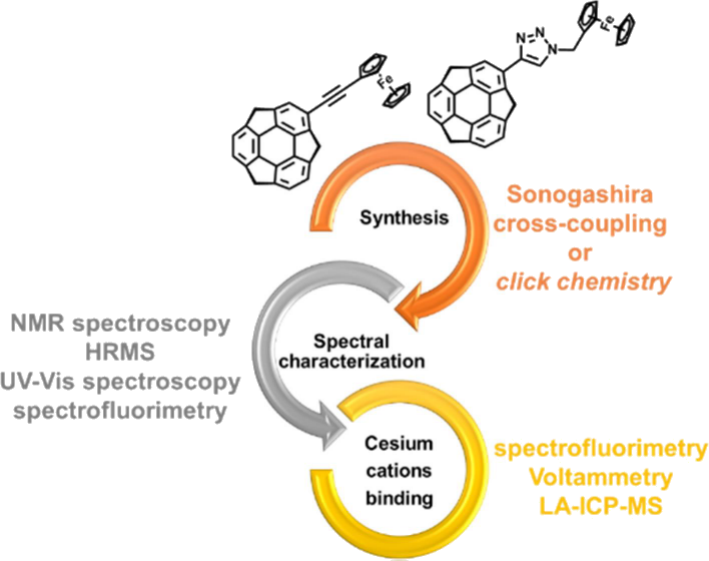

This paper reports the synthesis and characterization of novel monoferrocenylsumanenes obtained by means of the Sonogashira cross-coupling or *click chemistry* reaction as well as their application in cesium cation electrochemical sensors. A new synthetic protocol based on Sonogashira cross-coupling was developed for the synthesis of monoferrocenylsumanene or ethynylsumanene. The *click chemistry* reaction was introduced to the sumanene chemistry through the synthesis of 1,2,3-triazole containing monoferrocenylsumanene. The designed synthetic methods for the modification of sumanene at the aromatic position proved to be efficient and proceeded under mild conditions. The synthesized sumanene derivatives were characterized by detailed spectroscopic analyses of the synthesized sumanene derivatives. The supramolecular interactions between cesium cations and the synthesized monoferrocenylsumanenes were spectroscopically and electrochemically investigated. Furthermore, the design of the highly selective and sensitive cesium cation fluorescence and electrochemical sensors comprising the synthesized monoferrocenylsumanenes as receptor compounds was analyzed. The tested cesium cation electrochemical sensors showed excellent limit of detection values in the range of 6.0–9.0 nM. In addition, the interactions between the synthesized monoferrocenylsumanenes and cesium cations were highly selective, which was confirmed by emission spectroscopy, laser ablation inductively coupled plasma mass spectrometry (LA-ICP-MS), and cyclic voltammetry.

## Introduction

**Figure 1:**
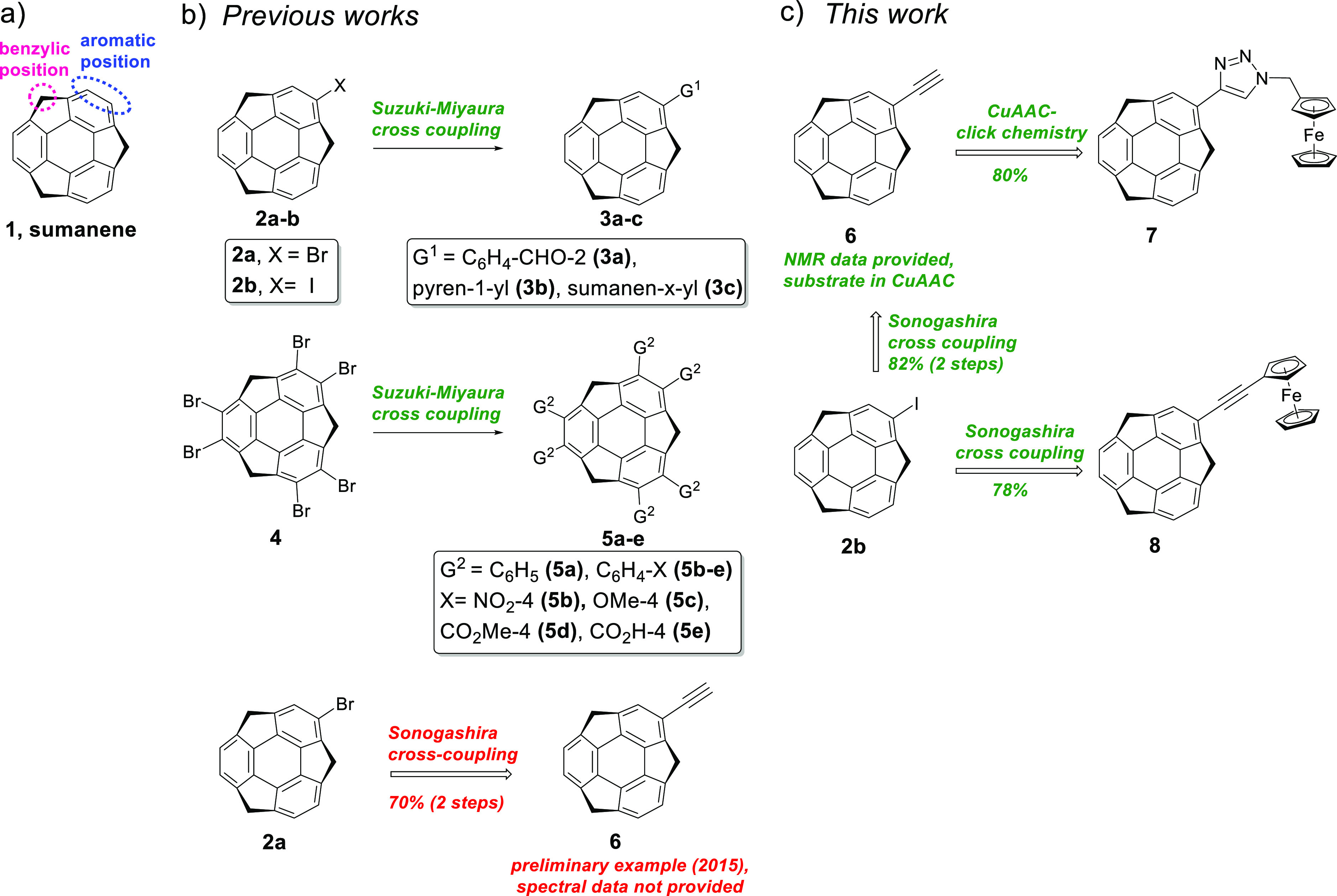
(a) Structure of sumanene (**1**). (b) Previous reports on the Suzuki–Miyaura or Sonograshira cross-coupling reactions with sumanene derivatives. (c) Graphical representation of the aims of this work.

Sumanene (**1**; Figure 1a), a bowl-shaped compound, is a subunit of fullerene C_60_. Due to its unique shape, sumanene has been widely studied over the last 15 years.^1−5^ From the structural viewpoint, sumanene (**1**) comprises both aromatic and benzylic positions. It is well established that sumanene (**1**) can be modified at the aromatic position by means of electrophilic aromatic substitution (S_E_Ar).^2,4,6−11^ Thus far, various sumanene derivatives modified at the aromatic position have been reported, which include 2-nitrosumanene,^8^ 2-formylsumanene,^8^ 2-acetylsumanene,^8^ 2-iodosumanene,^8,12^ 2-bromosumanene,^4^ 2,3,5,6,8,9-hexabromosumanene,^9−11^ and acylated sumanene derivatives.^13^

The successful synthesis of halogenated sumanenes through the S_E_Ar reaction provided access to the cross-coupling reactions toward carbon–carbon-bond-expanded sumanene derivatives. The synthetic utility of the Suzuki–Miyaura cross-coupling reaction in sumanene chemistry has been documented over the years (Figure 1b). In previous studies, 2-bromosumanene (**2a**),^7,14^ 2-iodosumanene (**2b**),^7,12^ or hexabromosumanene (**4**)^9,10^ was subjected to Suzuki–Miyaura cross-coupling reaction with various aryl boronic acid partners. This yielded promising sumanene derivatives, such as π-expanded bowls (**3a–3c**, **5a–5e**), or the starting material for a hydrogen-bonded organic framework^9^ (**5e**). However, despite the significant progress in sumanene chemistry since its first synthesis in 2003,^1^ the modification of sumanene at the aromatic position via the Sonogashira cross-coupling reaction, which introduces an acetylene linkage to the aromatic skeleton, remains yet unexplored. To date, 2-ethynylsumanene^7^ (**6**; Figure 1b) is the only known preliminary example of a sumanene derivative modified at the aromatic position by the Sonogashira cross-coupling reaction. The reported two-step synthesis of compound **6** began with 2-bromosumanene (**2a**), and the combined yield was close to 70%. However, **6** was a part of unpublished results, and neither a detailed synthetic protocol nor spectral data were provided for this compound. Despite low yield (12% by MALDI-MS, no spectral data available), Glaser homocoupling of **6** indicated the synthetic potential of sumanene-containing alkynes.^7^

Alkyne-containing reactants are versatile starting materials for synthesizing functional organic compounds through the copper-catalyzed azide-alkyne cycloaddition (CuAAC; *click chemistry*) reaction.^15−17^ In fact, the *click chemistry* reaction has not been utilized so far for the modification of sumanene at the aromatic position. Therefore, in order to design new methods for the sumanene modification, we decided to develop a new, efficient protocol for the synthesis of 2-ethynylsumanene (**6**) starting from 2-iodosumanene (**2b**). We characterized **6** by spectroscopy and examined the reactivity of 2-ethynylsumanene (**6**) in the *click chemistry* reaction toward the synthesis of novel 1,2,3-triazole containing monoferrocenylsumanene **7** (Figure 1c). We also aimed to synthesize Sonogashira cross-coupling-derived monoferrocenylsumanene **8** (Figure 1c). In addition, based on our recent studies on the use of specific sumanene-ferrocene conjugates in the detection of cesium cations in liquid samples,^18,19^ we investigated whether the newly synthesized monoferrocenylsumanenes **7** and **8** could act as effective building units of cesium cation fluorescence and electrochemical sensors. The environmental significance of developing novel receptors for the identification of cesium cations in solution stems from the discovery of considerable levels of radioactive cesium isotopes in the postdisaster sites of nuclear plant accidents in Fukushima (2011) and Chernobyl (1986).^20−25^ Because of its relatively long half-life (30 years) and presence in oceans and groundwater, a significant increase in the amount of radioactive cesium in the environment is harmful. Cesium pollution is thus hazardous and has severe impact on aquatic species, as well as agriculture, farming, and human health. Therefore, it is critical to design new methods for effective and selective monitoring of cesium concentration. This paper describes the construction of electrochemical sensors comprising **7** or **8** as a receptor layer which are dedicated to quick, selective, and effective recognition of cesium cations in solution.

## Results and Discussion

### Synthesis

**Scheme 1:**
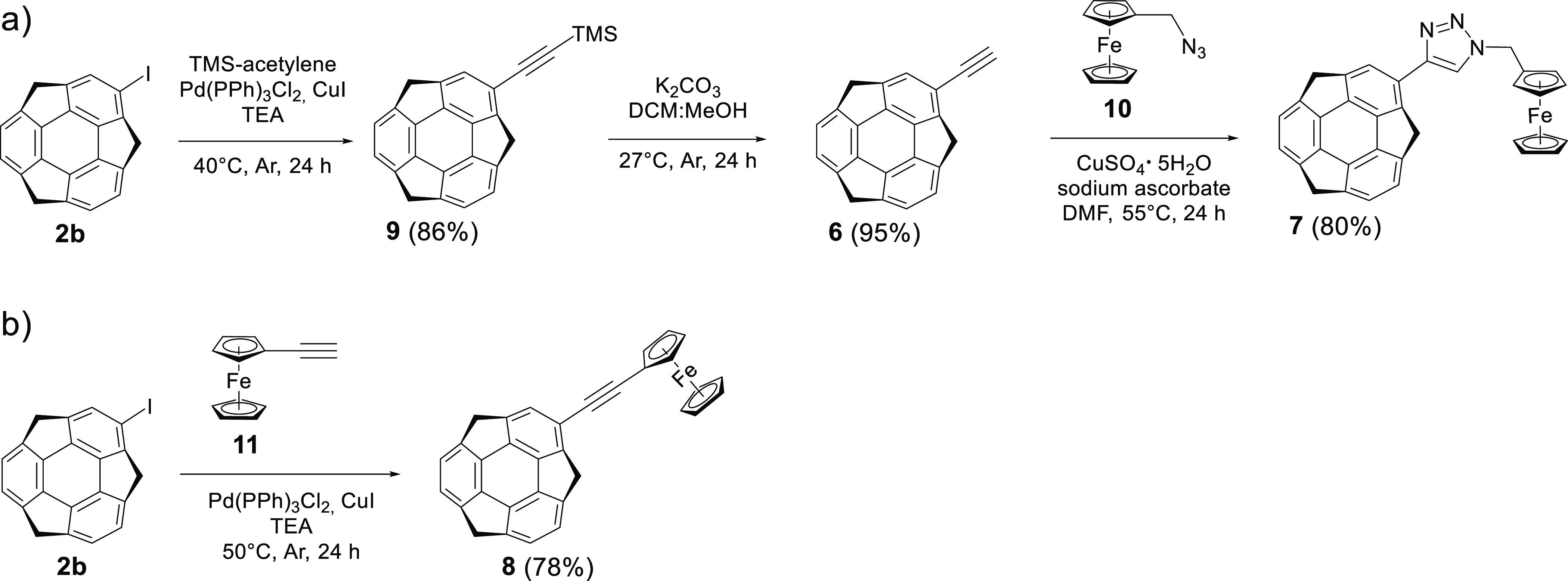
(a) Synthesis of Monoferrocenylsumanene **7** by the CuAAC (*Click Chemistry*) Approach, (b) Synthesis of Compound **8** by the Sonogashira Cross-Coupling Reaction

The synthesis of 2-ethynylsumanene **6** and monoferrocenylsumanenes **7** and **8** is presented in Scheme 1. For experimental details, see the Experimental Section and Supporting Information, Section 1.

First, the CuAAC-originating compound **7** was synthesized as follows (Scheme 1a). The three-step procedure, which starts from the key substrate, 2-iodosumanene **6**, involved (a) preparation of trimethyl(sumanen-2-ylethynyl)silane (**9**) from 2-iodosumanene (**2b**) and trimethylsilylacetylene using the Sonogashira cross-coupling reaction (86% yield), (b) desilylation of **9** (95% yield), and finally, (c) the CuAAC (*click chemistry*) reaction of **6** with ferrocenemethylazide **10** (80% yield). Owing to the efficient preparation of the intermediate **6** (82% yield vs the previously reported yield of 70%^7^), the three-step yield of the target compound **7** was very satisfactory.^26^

Synthesis of compound **8** by Sonogashira cross-coupling (Scheme 1b) involved the reaction between ethynylferrocene (**11**) and 2-iodosumanene (**2a**), selected among 2-halosumanenes (**2a** and **2b**; see details in the Experimental Section). In brief, the process was performed in triethylamine, using it as both the base and the solvent, and with the addition of bis(triphenylphosphine)palladium(II) dichloride (Pd(PPh_3_)_2_Cl_2_) and copper(I) iodide (CuI) as catalysts (Scheme 1). Under these conditions, monoferrocenylsumanene **8** was successfully synthesized with a good yield (78%).

### NMR Analysis

**Figure 2:**
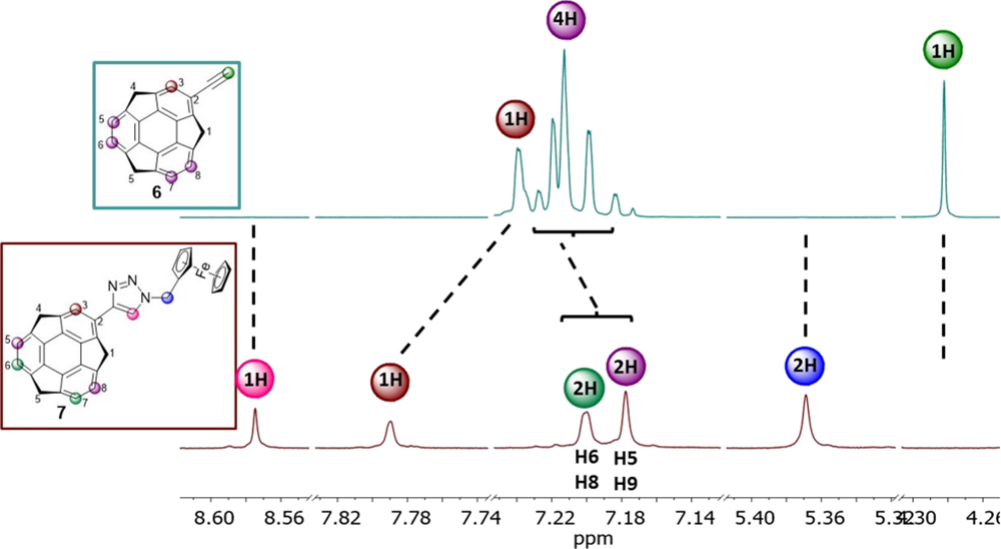
Selected insets of the ^1^H NMR spectra (500 MHz, DMSO-*d*_6_; 2-ethynylsumanene **6**—top and monoferrocenylsumanene **7**—bottom). For the complete spectra comparison, see the Supporting Information, Section 2, Figure S19.

1D and 2D NMR spectroscopy analyses were carried out to thoroughly characterize compounds **2a**, **2b**, and **6–8**. Full spectral data for compounds **1**, **2a**, **2b**, **6**–**9** are provided in Supporting Information, Sections 1 and 2. The spectra of target compounds **7** and **8** were markedly different from those of parent compounds **2b** and **6**, respectively. For example, the main differences between the spectra of representative compounds **6** and **7** are described herein. A comparison of the spectra of **2b** and **8** is presented and discussed in Figure S26, Section 2, Supporting Information. In a relation to the ^1^H NMR spectrum of compound **6**, the spectrum of compound **7** showed (Figure 2) (a) the singlets at 5.37 ppm (the CH_2_ protons, blue) and 8.57 ppm (the 1,2,3-triazole proton, pink) in the place of the singlet at 4.28 ppm (C(*sp*)-H in compound **6**, green), and (b) the signal of the sumanene aromatic proton from the substituted ring (brown), which was well separated from the signals of aromatic protons from the unsubstituted ring (violet). Signals of the ferrocene protons/carbon nuclei were also observed in the corresponding spectra of compound **7**.

Our NMR analyses revealed the most significant changes in the chemical shifts of signals corresponding to the H1_endo_ proton in the examined compounds **2a**, **2b**, and **6–8**. We have used 1D and 2D NMR (^1^H-^13^C HMBC and ^1^H-^13^C HSQC) to explain the distinct ^1^H NMR profiles of compounds **2a**, **2b**, and **6–8** (corresponding spectra are provided in the Supporting Information, Section 2). A detailed discussion on the NMR signal assignments and diagnostic 2D NMR correlations in the studied compounds can be found in the Supporting Information, Section 3.

### Photophysical Properties of Monoferrocenylsumanenes **7** and **8**

**Figure 3:**
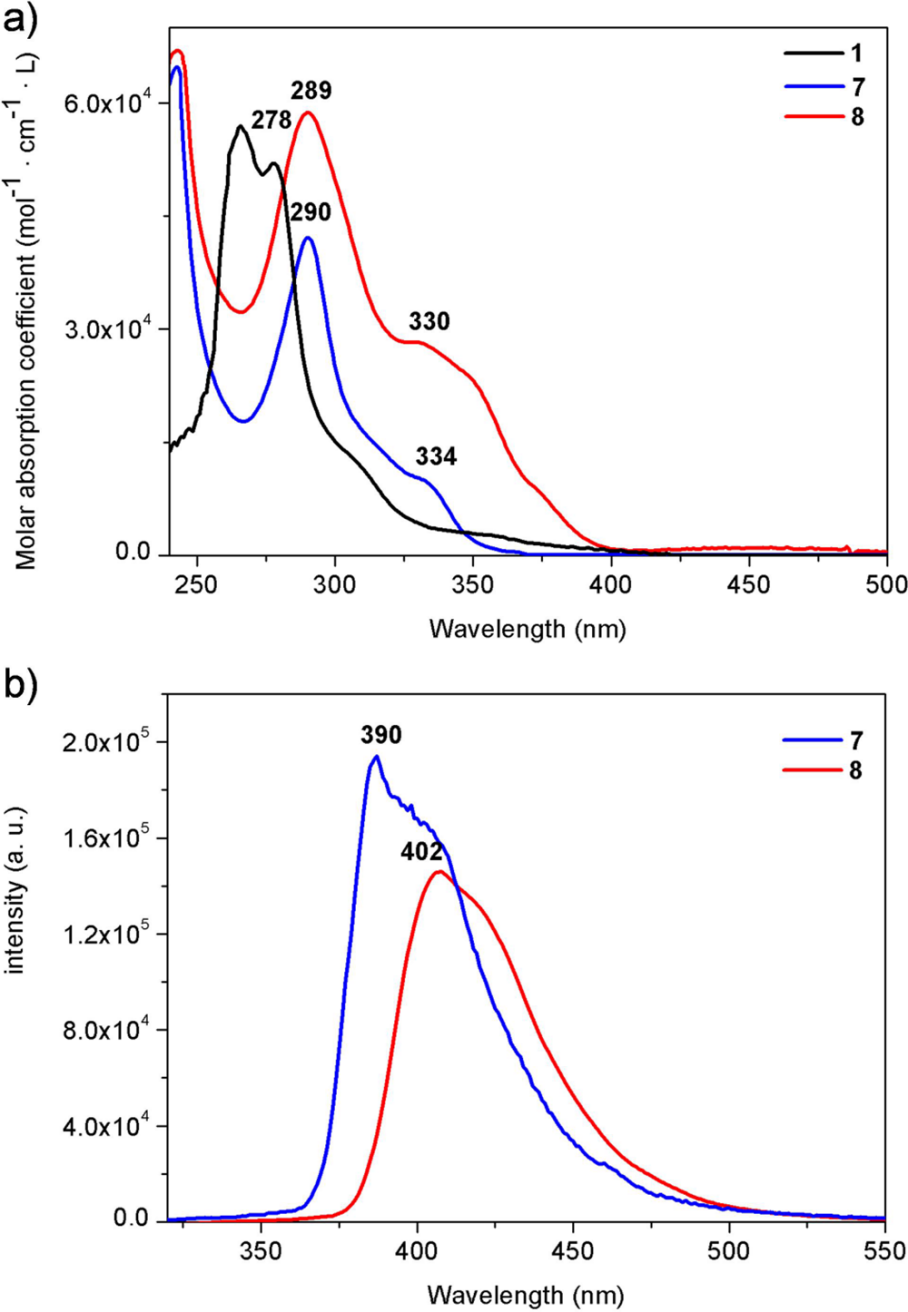
(a) UV–vis spectra of **1**, **7**, and **8**. (b) Emission spectra of **7** and **8**. Solvent: CHCl_3_, concentration: 2 × 10^–5^ M, λ_exc_ = 285 nm.

Absorption and emission spectra of monoferrocenylsumanenes **7** and **8** are presented in Figure 3. The absorption spectra of **7** and **8** (Figure 3a) were similar, with the absorption maxima (λ_max_) observed at 289–290 and 330–334 nm. The absorption maximum located at 289–290 nm was red-shifted compared to the unmodified sumanene (**1**; 278 nm). It was attributed^13,18,27^ to the π-extended conjugation in **7** and **8**. Molar absorption coefficient values for **8** were ca. 1.4-fold higher than that for **7**. It is related to better π-conjugation between sumanene and ferrocene units in **8** in comparison to **7**, due to the presence of the acetylene linkage between sumanene and ferrocene motifs in **8**. The electron transfer between those motifs in **7** is not so enhanced because of the presence of the methylene linkage. Theoretical calculations are also consistent with this experimental result, with the HOMO and LUMO of **7** and **8** delocalized to the triazole and ethynylferrocene moieties, respectively. The calculated HOMO–LUMO energy gaps are smaller in the order **1** > **7** > **8**, suggesting that **8** is the most effectively extended π-conjugated system (calculated energy level and Kohn–Sham orbitals at the HOMO and LUMO of **1**, **7**, and **8** are presented in Figure S35 in the Supporting Information). A red shift in comparison to unmodified sumanene (**1**) was also observed in the emission spectra of **7** and **8** (Figure 3b; emission maximum for **1** is located at ca. 375 nm^28^). This redshift is higher for **8** (λ_max_ = 402 nm) than that for **7** (λ_max_ = 390 nm). This is a result of π-conjugation between sumanene and ferrocene moieties in **8** in comparison to **7**. The emission intensity for **7** was ca. 1.4-fold higher in comparison to **8**.

### Supramolecular Interactions between Monoferrocenylsumanenes **7** and **8** and Cesium Cations

**Figure 4:**
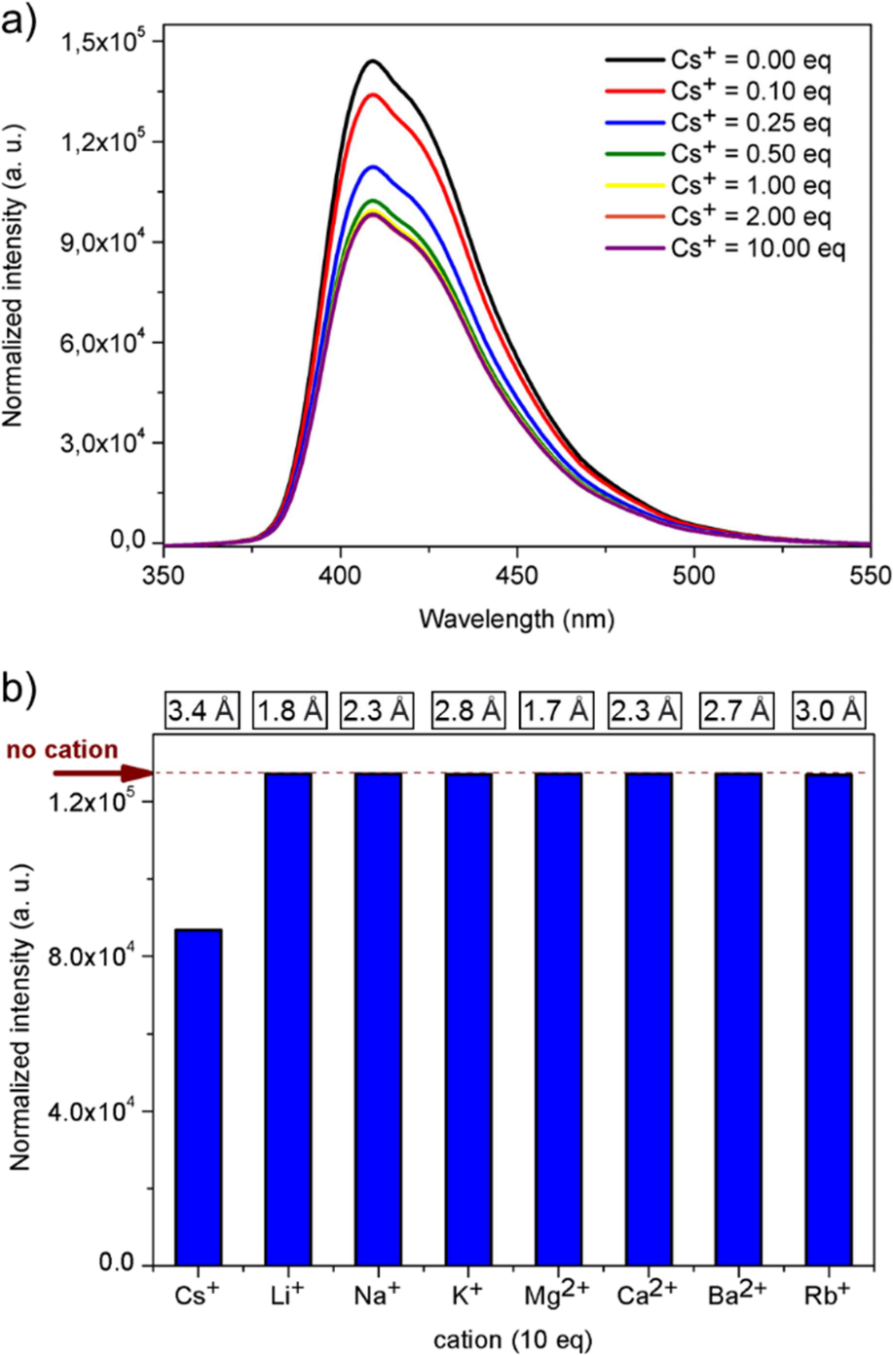
(a) Emission spectra (λ_ex_ = 285 nm) of monoferrocenylsumanene **8** in the presence of various amounts (equivalents = equiv) of cesium cations. (b) Emission intensity (λ_max_ = 402 nm) values for **8** in the presence of the excess (10 equiv) of given cations (emission intensity for **8** in the absence of cations is also marked with the arrow). The values of van Der Waals radius for a given cation are given in the top of the figure. Solvent: CHCl_3_–CH_3_OH (1/1 v/v).

Inspired by our recent reports^18,19^ on the use of selected ferrocene–sumanene conjugates as cesium cation (Cs^+^) molecular receptors, we investigated supramolecular interactions between Cs^+^ and newly synthesized monoferrocenylsumanenes (**7** or **8**) with emission spectroscopy. The spectra were measured in the methanol–chloroform mixture (1:1 v/v) in order to ensure the solubility of both monoferrocenylsumanene (**7** or **8**) and Cs^+^ (in the form of CsCl). Basically, the spectra of monoferrocenylsumanene (**7** or **8**) were measured in the presence of increasing amounts of Cs^+^ (from 0 up to 10 equiv). The results for the representative monoferrocenylsumanene **8** are presented in Figure 4a. Upon the addition of Cs^+^, a turn-off fluorescence behavior was observed. It suggested the supramolecular interaction between Cs^+^ and the anionic concave face of the sumanene bowl within the structure of **7** or **8** (calculated electrostatic potential (ESP) of **1**, **7**, and **8** from the concave face of the sumanene bowl are presented in Figure S36 in the Supporting Information). A further decrease in the emission intensity further was noted with the addition of further portions of Cs^+^. The changes between the respective spectra were not the same. This was related to the stoichiometry of the noncovalent systems formed. The stoichiometry of the complexes, which was analyzed with the continuous variation method (Job’s plot method), was 1:2 in the case of both monoferrocenylsumanenes (that is Cs^+^: **7** and Cs^+^: **8**; the Job’s plots are provided in the Supporting Information, Section 5). It was ascribed to the presence of dynamically formed sandwich-type complexes^18,19,29−31^ composed of one cesium cations and two sumanene bowls at their neutral state. To determine the apparent binding constant (*K*_app_) values for those systems, the Benesi–Hildebrand method^29,30,32,33^ was applied. The *K*_app_ values estimated for **7** and **8** were relatively high and equaled 5.9 × 10^5^ and 8.7 × 10^5^ mol^–2^ L^2^, respectively (for data and figures, see the Supporting Information, Section 5). Taking into account the slight differences in bowl depth values for **7** and **8** (1.16 and 1.19 Å, respectively; Figure S37 and discussion in Section 4 in the Supporting Information,), it may be assumed that a slightly deeper sumanene bowl may be correlated with a higher apparent binding constant value.

To get an insight into the selectivity of the studied interaction, the emission spectra of **8** were also measured in the presence of an excess (10 equiv) of other cations, namely, Li^+^, Na^+^, K^+^, Mg^2+^, Ca^2+^, Ba^2+^, and Rb^+^. The obtained results are graphically presented as a column chart in Figure 4b (the corresponding spectra are provided in the Supporting Information, Section 5). It was observed that the emission intensity of **8** decreased significantly only in the presence of Cs^+^. This indicates that supramolecular interactions between Cs^+^ and the studied monoferrocenylsumanenes are highly selective. This high selectivity can be ascribed to the perfect size match between the sumanene bowl and van der Waals radius of Cs^+^, as demonstrated in both theoretical^34,35^ and experimental^19,29−31^ studies. For general comparison, the van der Waals radius values for the tested cations are presented in Figure 4b.

**Figure 5:**
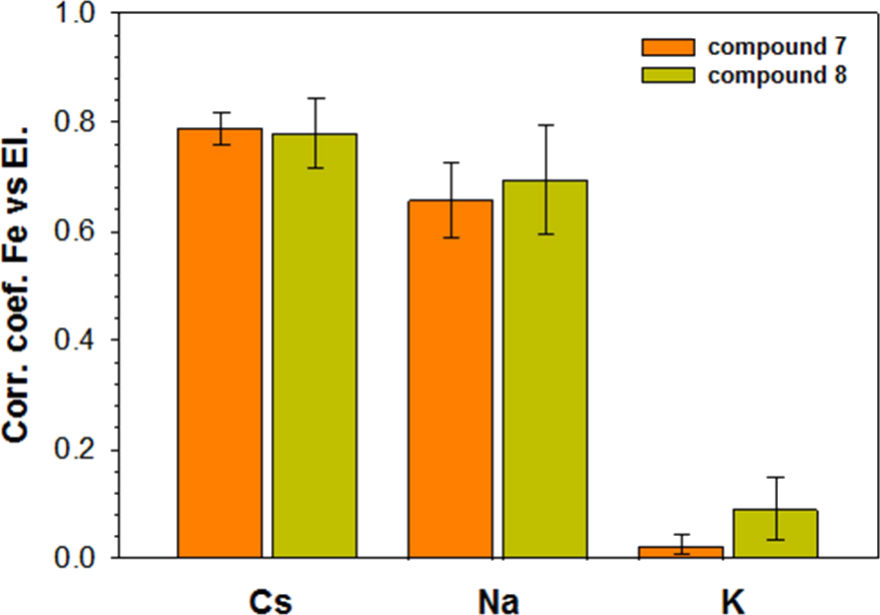
Correlation coefficients calculator for the co-occurrence of respective elements (Cs, Na, and K) and the Fe distribution over the samples of compound **7** and compound **8**.

The abovementioned spectrofluorimetric tests demonstrated the high selectivity of the interaction between Cs^+^ and monoferrocenylsumanenes **7** or **8**. To further confirm this high selectivity, the samples of monoferrocenylsumanenes **7** and **8** were subjected to laser ablation inductively coupled plasma mass spectrometry (LA-ICP-MS). The multi-line ablation and the resulting maps of elemental distribution are presented in Section 7 in the Supporting Information (Table S4 therein). The scale of the maps is normalized to the highest signal registered for each element individually, and the relative distribution allows the calculation of the correlation for the respective element over Fe distribution. Additionally, the edge of one sample was tested to examine the eventual influence of the observed inhomogeneity on the results. Although macroscopically the samples appeared heterogeneous, the results obtained were consistent for all repeated measurements. Fluctuations of transient signals registered for 10 lines (5 lines of ablation per each sample) were used to calculate the values of correlation coefficient for Na, K, and Cs versus Fe, which are shown in Figure 5. Ba was excluded from the calculations as a very low signal was registered for this element in the case of both samples that were examined by LA-ICP-MS. The highest elemental correlation values were obtained for Na and Cs (*r*^2^ = 0.757 ± 0.213 and *r*^2^ = 0.713 ± 0.099, respectively). Although their behavior seemed similar, the repeatability of Na distribution was lower than the repeatability of the distribution of Cs and Fe.

### Application of Monoferrocenylsumanens **7** and **8** as Receptor Units in Cesium Cation Electrochemical Sensors

**Figure 6:**
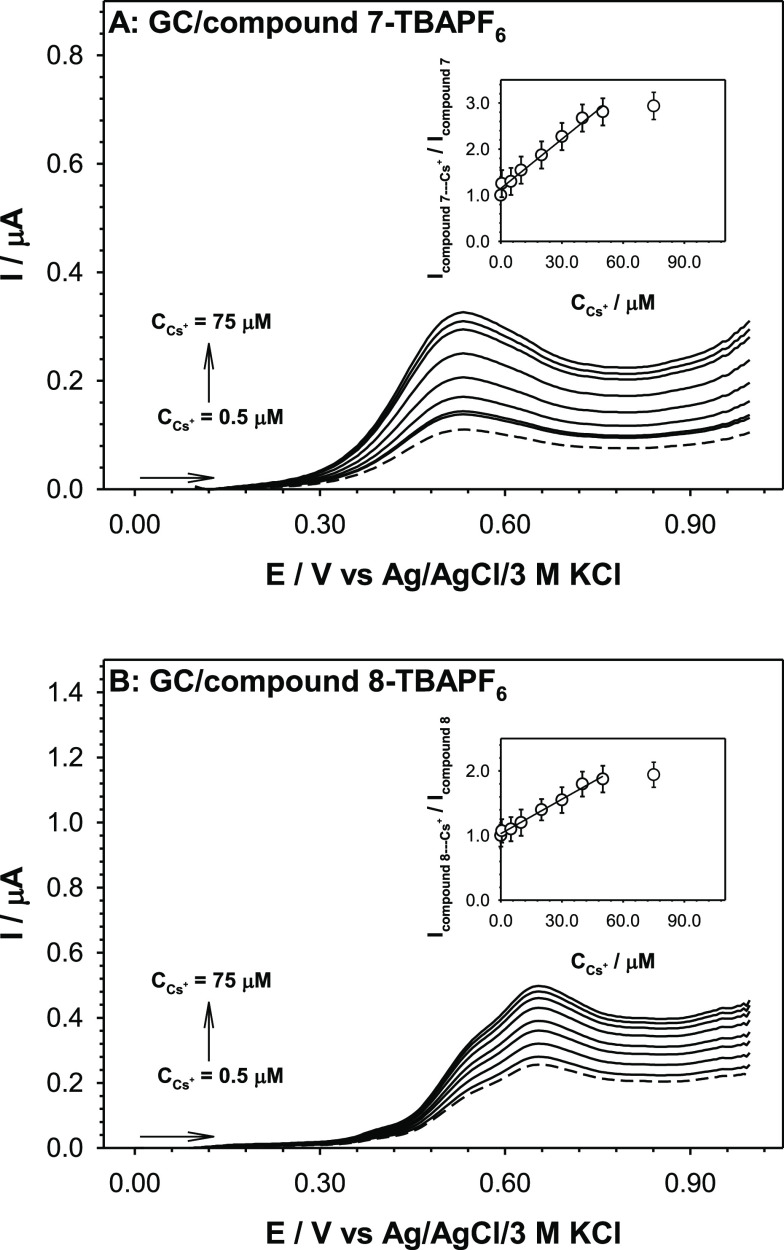
DPV (plotted in IUPAC convention) of recognition layers containing compound **7** (A) and compound **8** (B) recorded in 50 mM TBABF_4_ aqueous solution with the addition of Cs^+^. Inset: Dependencies of the ratio (*I*_compound–Cs+_/*I*_compound_) vs Cs^+^ concentration. Experimental conditions: solvent: water, supporting electrolyte: tetrabutylammonium tetrafluoroborate (TBABF_4_), analyte: Cs^+^ (CsNO_3_), *C*_compounds_ = 0.02 mM, *C*_TBABF4_ = 50 mM, *C*_Cs+_ = 0.5–75 μM, *T* = 21 °C, modulation time: 0.002 s, interval time: 0.1 s, modulation amplitude: 0.04995 V, working electrode: disc glassy electrode (ϕ = 3 mm), counter electrode: Pt plate (*A* = ca. 1 cm^2^, reference electrode: Ag/AgCl/3 M KCl.

The cyclic voltammograms (CV) of compounds **7** and **8** were characterized by one pair of current signals (anodic and cathodic) corresponding to the characteristic Fe^2+/3+^ redox couple in the ferrocene unit^36−38^ (see voltammograms in Supporting Information, Figure S45). We found that the electrode process was not affected significantly by the linking of ferrocene units with sumanene through acetylene or 1,2,3-triazole linkages (see voltammograms and discussion in Section 6 in the Supporting Information). The flexibility of the linker influences the orientation of the compounds in the layer, and in a consequence, the position of the current signal on the potential scale. The acetylene linker shall be less flexible than the 1,2,3-triazole linker under voltammetric conditions. The location of the compound **8** current signal at higher potential values (ca. 0.66 V, see Figure 6B) indicates that the ferrocene units conjugated with sumanene through acetylene linkage are further away from the electrode surface and the electrooxidation process is more difficult.

Having electrochemically characterized monoferrocenylsumanenes **7** and **8**, we envisioned that those compounds can be used as receptor layers of cesium cation electrochemical sensors. As noted above, the mechanism of Cs^+^ detection by the studied sumanene derivatives is based on the selective cation-π electron interaction between Cs^+^ and the concave site of the sumanene moiety. Introducing the ferrocene unit to the sumanene skeleton enables the voltammetric detection of Cs^+^. In this work, we examined the influence of the type of linker in the monoferrocenylsumanene receptor on the sensitivity of Cs^+^ detection. To form the recognition layer, the surface of the glassy carbon electrode surface was modified with appropriate monoferrocenylsumanene receptor **7** or **8**. The sensor preparation is described in detail in Subsection 1.9 in the Supporting Information. Prior to Cs^+^ detection experiments, the recognition layers were cycled in 50 mM aqueous solution of tetrabutylammonium tetrafluoroborate (TBABF_4_) in the potential range 0.0–1.0 V until a stable voltammogram was obtained. The analytical characteristics of the sensors containing each monoferrocenylsumanene receptor were determined based on the changes in the ferrocene oxidation current signal. The representative differential pulse voltammograms (DPV) of the sensors recorded in the presence of Cs^+^ cations in the solution are presented in Figure 6. The proposed relationships clearly proved that monoferrocenylsumanene receptors are sensitive to Cs^+^, with compound **7** being more sensitive than **8** (as evidenced by more significant increases in the current signal). More significant recorded ferrocene current oxidation signals were observed with the increase in Cs^+^ concentration in solution. Considering our previous studies on tris-ferrocenylated sumanenes,^18,19^ we consider that the cesium cations interact with the cavity (concave site) of the sumanene bowl, by means of cation-π interactions. As a result, we hypothesize that cesium cations are entrapped between two sumanene bowls of **7** or **8**. This mode of interactions can also be hypothesized analyzing the crystal structure of the sumanene–cesium complex,^31^ even though this complex is electronically different from the electronically neutral monoferrocenylsumanenes described herein. As a result of this process, there is a rearrangement of the ferrocene units in the receptor layer. Both the distance of ferrocene units between each other and the surface of the electrode change, making electron exchange easier. In consequence, the intensity of the Fc oxidation current signal increases with the increase of Cs^+^ concentration.

The limits of detection (LODs) were estimated from the linear regression equations of the calibration plots (refer to insets in Figure 6), taking into account the mean background value (ferrocene oxidation signal in the 50 mM TBABF_4_ aqueous solution without the addition of Cs^+^) plus 3 standard deviations of the background (*x̅* + 3σ_*x*_; *n* = 5). The estimated LOD values equaled 6.0 and 9.0 nM for compounds **7** and **8**, respectively. Comparison of LOD values for the designed voltammetric sensors comprising **7** or **8** as a receptor and reported sumanene–ferrocene conjugates is presented in Table 1. Notably, these LOD values for **7** or **8** containing sensors were the most satisfactory, being ca. 2–3-fold lower than the value for the previously reported tris(ferrocenylmethidene)sumanene containing sensor (LOD = 20 nM).^18^ The herein presented receptors (**7**, **8**) also featured the most satisfactory *K*_app_ values.

**Table 1:** Comparison of the *K*_app_ and LOD Values for Electrochemical Sensors Dedicated to the Recognition of Cs^+^ Comprising Compounds **7**–**8** and Reported Sumanene–Ferrocene Conjugates

entry	compound	*K*_app_ (mol^–2^ L^2^)^a^	LOD (nM)^b^	ref.
1	**7**	5.9 × 10^5^	6	this work
2	**8**	8.7 × 10^5^	9	this work
3	tris(ferrocenylmethidene)sumanene	N/D^c^	20	(18)
4	various monoferrocenylsumanenes	from 1.7 × 10^4^ to 4.5 × 10^5^	N/D^d^	(19)

a Estimated from fluorescence spectroscopy.

b Estimated from voltammetry.

c *K*_app_ values for the structurally similar tris-substituted sumanene derivatives^29,30^ ranges from 2.9 × 10^4^ to 9.0 × 10^5^ mol^–2^ L^2^.

d LOD for these derivatives, estimated from fluorescence spectroscopy, is from 120 to 700 nM.

Finally, we further investigated the selectivity of the Cs^+^ recognition with cyclic voltammetry. No significant changes were observed in the ferrocene oxidation signal in the presence of other cations like Ba^2+^, Na^+^, and K^+^ (see in Figure S45, Section 6, Supporting Information). This result is in a good agreement with the spectrofluorimetric and LA-ICP-MS experiments.

## Conclusions

In order to gain a deeper understanding of the chemistry and application of sumanene, we synthesized new monoferrocenylsumanenes **7** and **8** and analyzed their supramolecular interactions with cesium cations. We designed an efficient (78–86%) protocol based on the Sonogashira cross-coupling reaction for the synthesis of new sumanene derivatives **8** and **9**. We also attempted to synthesize 2-ethynylsumanene (**6**) and achieved a high combined yield (82%). Our study was the first to successfully modify sumanene at the aromatic position through the *click chemistry* reaction by successful derivatization of 2-ethynylferrocene (**6**) to novel 1,2,3-triazole containing monoferrocenylsumanene **7**. The discussed *click chemistry* reaction was very efficient (80%), and the process was performed on air and proceeded under mild conditions. Absorption and emission spectroscopy analyses on monoferrocenylsumanenes **7** and **8** indicated π-conjugation within these derivatives. We also studied the interactions between cesium cations and monoferrocenylsumanenes (**7** or **8**) and found that they were highly selective, as evidenced by emission spectroscopy and LA-ICP-MS and cyclic voltammetry. The apparent binding constant for the dynamically formed systems, which was estimated by emission spectroscopy, was in the range of 5.9–8.7 × 10^5^ mol^–2^ L^2^. Additionally, monoferrocenylsumanenes **7** and **8** were electrochemically characterized by cyclic voltammetry, which revealed one pair of current (anodic and cathodic) signals characteristic of the ferrocene moiety. Based on the obtained results, the newly synthesized sumanene derivatives **7** and **8** were used to construct the receptor layers of cesium cation electrochemical sensors. The constructed sensors showed a satisfactory LOD, ranging from 6.0 to 9.0 nM. Notably, the LOD value for the sensor containing tris(ferrocenylmethidene) sumanene that was previously reported was 0.02 μM. (20 nm).^18^ Due to the presence of three ferrocene moieties at the benzylic positions in each sumanene skeleton, tris(ferrocenylmethidene)sumanene is a more structurally improved sumanene derivative in comparison to compounds **7** and **8**. Thus, monoferrocenylsumanenes **7** and **8** might display better steric fit to bind cesium cations in comparison to tris(ferrocenylmethidene)sumanene, and therefore, sensors containing these compounds showed lower LOD values than the one containing tris(ferrocenylmethidene) sumanene. To sum up, this work not only improved the state of the art of sumanene modification methods but also expanded the knowledge about the application of ferrocene–sumanene derivatives in the design of cesium cation sensors.

## Experimental Section

### Materials and Methods

Chemical reagents and solvents for the synthesis were commercially purchased and purified according to the standard methods, if necessary. Thin layer chromatography (TLC) and preparative thin layer chromatography (PTLC) were performed using Merck Silica gel 60 F254 plates. An oil bath was used as a heating source for the reactions that required heating.

The NMR experiments were carried out using a Varian VNMRS 500 MHz spectrometer (^1^H NMR at 500 MHz, ^13^C{^1^H} NMR at 125 MHz) equipped with a multinuclear z-gradient inverse probe head. The spectra were recorded at 25 °C, and standard 5 mm NMR tubes were used. ^1^H chemical shifts (δ) were reported in parts per million (ppm) relative to the solvent signal, i.e., CDCl_3_: δ_H_ (residual CHCl_3_) 7.26 ppm, δ_C_ (residual CHCl_3_) 77.2 ppm; DMSO-*d*_6_: δ_H_ (residual DMSO) 2.50 ppm, δ_C_ (residual DMSO) 39.5 ppm. Spin–spin coupling constant values (*J*) were given in Hz and were calculated using the Resolution Booster processing mode. NMR spectra were analyzed with the MestReNova v12.0 software (Mestrelab Research S.L).

ESI-HRMS (TOF) measurements were performed with a Q-Exactive ThermoScientific spectrometer.

UV–vis measurements were performed with the PerkinElmer spectrometer Lambda 25 at room temperature in a quartz cuvette of 1 cm length of the optical window. For the UV–vis measurements, the wavelengths for the absorption maxima λ_max_ were reported in nm. Spectrofluorimetric analyses were performed with a Hitachi F-4500 fluorescence spectrophotometer with a spectral resolution of 1 nm, and the wavelengths for the emission maxima were reported in nm.

Cyclic voltammetry (CV) and differential pulse voltammetry (DPV) experiments were performed in the three-electrode system with using an Autolab potentiostat, model PGSTAT 12. The disc glassy carbon electrode (GC; ϕ = 3 mm) was used as a working electrode, the Ag/AgCl/3 M KCl as a reference electrode, and the platinum plate with an area of at least 1 cm^2^ as a counter electrode. To minimize the electrical noise, all experiments were carried out in the Faraday cage. The electrochemical characteristic of the studied ferrocene derivatives (compound **7** and compound **8**) was done in dichloromethane (DCM) with the addition of tetrabutylammonium hexafluorophosphate (TBAPF_6_) as a supporting electrolyte. The concentration of the studied ferrocene derivatives was 0.02 mM. Dry dichloromethane (DCM, Sigma-Aldrich), tetrabutylammonium hexafluorophosphate (TBAPF_6_, Sigma-Aldrich), tetrabutylammonium tetrafluoroborate (TBABF_4_, Sigma-Aldrich), cesium nitrate (CsNO_3_, Sigma-Aldrich), potassium nitrate (KNO_3_, Sigma-Aldrich), sodium nitrate (NaNO_3_, Sigma-Aldrich), barium nitrate (Ba(NO_3_)_2_, Sigma-Aldrich), and perfluorinated resin solution containing Nafion (Nafion, Sigma-Aldrich) were used in electrochemical studies without additional purification.

The experimental data for LA-ICP-MS experiments were as follows. The Nd:YAG laser ablation system (LSX-213, CETAC, USA) was coupled to an ICP-MS mass spectrometer (NexION 300D, Perkin Elmer, USA). The laser beam wavelength of λ = 213 nm, energy of 3 mJ, and diameter of 100 μm were used to ablate the surface layers of the analyzed samples. During multi-line ablation (*n* = 5, 20 Hz) with the constant scan rate of 100 μm/s, transient signals were registered for ^23^Na, ^39^K, ^57^Fe, ^133^Cs, and ^137^Ba. The operating conditions of the used ICP-MS system are given in the Supporting Information, Table S1. For each isotope, raw signals were individually background-corrected for Ar flow before the start of ablation. Spikes were defined as a single data point exceeding the intensities of the neighboring data for more than 2 times. They were replaced with the average value calculated based on the intensities of two neighboring signals. All recalculations were done with the use of a custom written formula in Excel (Microsoft Corp.).

Preparation of the cesium cation electrochemical sensor: the glassy carbon surface was first polished at the microcloth polishing pad with a slurry of alumina (1.0 μm diameter, Buehler). Then, the GC surface was rinsed with distilled water to remove the Al_2_O_3_ residues. In the next step to produce more carboxyl groups at the GC surface, the electrode was oxidized in 0.1 M H_2_SO_4_ by cycling in the potential range −0.35 to 1.3 to −0.35 V with a scan rate of 100 mV s^–1^. On such a prepared electrode surface, the 10 μL droplet of 0.02 mM monoferrocenylsumanene **7** or monoferrocenylsumanene **8** solution (CH_2_Cl_2_: DMSO (1:1 v/v)) with the addition of 150 mM TBAPF_6_ and 5% Nafion was placed and left to dry in a desiccator.

### Synthesis

#### Synthesis of 4,7-Dihydro-1H-tricyclopenta[*def*,*jkl*,*pqr*]triphenylene (Sumanene; **1**)

Sumanene (**1**) was synthesized following a literature procedure.^1^

^1^H NMR (CDCl_3_, 500 MHz, ppm), δ_H_ 7.10 (s, 6H), 4.71 (d, ^2^*J*_H–H_ = 18.1 Hz, 3H), 3.42 (d, ^2^*J*_H–H_ = 18.1 Hz, 3H); ^1^H NMR (CDCl_3_, 500 MHz, ppm), δ_H_ 7.18 (s, 1H), 4.70 (d, ^2^*J*_H–H_ = 18.3 Hz, 3H), 3.50 (d, ^2^*J*_H–H_ = 18.3 Hz, 3H).

#### Synthesis of 2-Bromo-4,7-dihydro-1H-tricyclopenta[*def*,*jkl*,*pqr*]triphenylene (2-Bromosumanene, **2a**)

2-Bromosumanene (**2a**) was synthesized from sumanene (**1**) following a literature procedure,^4^ with slight modifications in reaction time. To a stirred solution of sumanene (50.0 mg, 0.188 mmol, 1 equiv) in CH_2_Cl_2_ (17 mL), a solution of pyridinium hydrobromide perbromide (120.0 mg, 0.377 mmol, 1.8 equiv) in CH_3_CN (8.5 mL) was added dropwise at 0 °C. The reaction mixture was then stirred at 27 °C for 4 h. The reaction was quenched with sat. Na_2_S_2_O_3_ (7 mL) and sat. NaHCO_3_ (7 mL), and the crude product was extracted with CH_2_Cl_2_ (3 × 30 mL). Organic layers were combined and washed with water and brine. After drying with MgSO_4_ followed by filtration, volatiles were distilled off on a rotary evaporator. The product was purified using a preparative thin layer chromatography (PTLC; SiO_2_, 25% hex/ CH_2_Cl_2_) to provide 2-bromosumanene (**2a**) as a white solid (61.3 mg, 95%).

^1^H NMR (CDCl_3_, 500 MHz, ppm), δ_H_ 7.21 (s, 1H), 7.17–7.10 (m, 4H), 4.72–4.65 (m, 3H), 3.50 (d, ^2^*J*_H–H_ = 19.6 Hz, 1H), 3.44 (d, ^2^*J*_H–H_ = 19.5 Hz, 2H); ^13^C{^1^H} NMR (CDCl_3_, 125 MHz, ppm), δ_C_ 151.8, 149.3 × 2, 149.2, 149.0, 148.9, 148.7, 148.4 × 2, 148.2, 147.9, 147.4, 127.0, 124.2, 123.7, 123.6, 123.5, 116.7, 43.3, 41.9, 41.7.

#### Synthesis of 2-Iodo-4,7-dihydro-1H-tricyclopenta[*def*,*jkl*,*pqr*]triphenylene (2-Iodosumanene, **2b**)

2-Iodosumanene (**2b**) was synthesized from sumanene (**1**) following a literature procedure,^12^ using 1,3-diiodo-5,5-dimethylhydantoin (DIH) as the iodination reagent with a catalytic amount of trifluoroacetic acid (TFA).

^1^H NMR (CDCl_3_, 500 MHz, ppm), δ_H_ 7.43 (s, 1H), 7.17–7.09 (m, 4H), 4.73–4.66 (m, 3H), 3.43 (d, ^2^*J*_H–H_ = 19.6 Hz, 1H), 3.42 (d, ^2^*J*_H–H_ = 19.6 Hz, 1H), 3.34 (d, ^2^*J*_H–H_ = 19.6 Hz, 1H).

#### Synthesis of 2-(Ferrocenylethynyl)-4,7-dihydro-1H-tricyclopenta[*def*,*jkl*,*pqr*]triphenylene (Monoferrocenylsumanene, **8**)

2-Iodosumanene (**2b**; 14.4 mg, 0.04 mmol, 1 equiv), bis(triphenylphosphine)palladium(II) dichloride (Pd(PPh_3_)_2_Cl_2_; 2.6 mg, 0.0004 mmol, 0.1 equiv) and copper(I) iodide (CuI; 0.4 mg, 0.002 mmol, 0.05 equiv) were placed in a reaction flask. The content of the flask was evacuated and purged with argon. Triethylamine (TEA, 2.5 mL) was added, and the reaction mixture was stirred for 15 min at 50 °C under an argon atmosphere. A solution of ethynylferrocene (**11**; 11.6 mg, 0.06 mmol, 1.5 equiv) in TEA (1.5 mL) was added, and the reaction mixture was stirred for 24 h at 50 °C under an argon atmosphere. Distilled water (6 mL) was added, and the crude product was extracted with CH_2_Cl_2_ (3 × 20 mL). Organic layers were combined, washed with 2 M HCl (3 × 15 mL), water, and brine. After drying with MgSO_4_ followed by filtration, volatiles were distilled off on a rotary evaporator. The product was purified using two-step PTLC purification (SiO_2_; 1st purification: 25% CH_2_Cl_2_/hexane, 2nd purification: 50% THF/hexane) to provide the target monoferrocenylsumanene **8** as a yellow solid (14.8 mg, 78%), mp = 190–192 °C.

^1^H NMR (CDCl_3_, 500 MHz, ppm), δ_H_ 7.22 (1, 1H), 7.15–7.11 (m, 4H), 4.79–4.69 (m, 3H), 4.52–4.51 (m, 2H), 4.26–4.25 (t-like m, ^3^*J*_H–H_ = 1.8 Hz, 2H), 4.24 (s, 5H), 3.60 (d, ^2^*J*_H–H_ = 19.7 Hz, 1H), 3.44 (d, ^2^*J*_H–H_ = 19.4 Hz, 1H), 3.43 (d, ^2^*J*_H–H_ = 19.3 Hz, 1H); ^13^C{^1^H} NMR (CDCl_3_, 125 MHz, ppm), δ_C_ 150.7, 149.4, 149.2, 149.1, 149.0 × 2 (3C), 148.9, 148.8, 148.6, 148.5, 148.2 × 2, 126.7, 123.8, 123.7, 123.5 × 2, 118.8, 89.8, 85.4, 71.6 (2C), 70.2 (5C), 69.0. 65.6 (2C), 42.0, 41.9, 41.7; HRMS (ESI) *m/z* [M]^+^ calcd. For C_33_H_20_Fe 472.0909, found 472.0910; UV–Vis, λ_max_ (CHCl_3_, 2 × 10^–5^ M) 289, 330 nm; Emission spectrum (CHCl_3_, 2 × 10^–5^ M, λ_exc_ = 285 nm) 402 nm; *R*_f_ (50% THF/hexane) = 0.85.

Note 1: The major impurity in that reaction was 1,4-diferrocenylbuta-1,3-diyne (**12**; *R*_f_ (50% THF/hexane) = 0.88; a side product of Glaser coupling between two molecules of ethynylferrocene (**11**)). After the first PTLC separation (25% CH_2_Cl_2_/hexane), the presence of this side product was observed in the crude mixture. Second, PTLC separation (50% THF/hexane) enabled the isolation of the pure target product **8**.
Note 2: The formation of side products, namely, di-(ferrocenylethynyl)sumanenes (ca. 15 wt % of the mass of the crude mixture), was observed when the 2-iodosumanene sample that was prepared from sumanene (**1**) using the different method (employing gold(III) chloride and N-iodosuccinimide),^8^ was used in the Sonogashira cross-coupling reaction with ethynylferrocene (**11**). Those side products could be removed from the crude product using gel permeation chromatography (GPC; CHCl_3_); however, the yield of **8** in that synthesis was lower (ca. 65%).
Note 3: The **8** reaction yield starting from 2-bromosumanene (**2a**) is 37%.

#### Synthesis of 2-Ethynyl-4,7-dihydro-1H-tricyclopenta[*def*,*jkl*,*pqr*]triphenylene (2-Ethynylsumanene, **6**)

2-Ethynylsumanene (**6**) was synthesized in two steps.

##### Step 1: Synthesis of ((4,7-Dihydro-1H-tricyclopenta[*def*,*jkl*,*pqr*]triphenylen-2-yl)ethynyl)trimethylsilane (Trimethyl(sumanenylethynyl)silane, **9**) from **2b**

2-Iodosumanene (**2b**; 25.0 mg, 0.064 mmol, 1 equiv), Pd(PPh_3_)_2_Cl_2_ (4.5 mg, 0.0064 mmol, 0.1 equiv), and CuI (1.0 mg, 0.0032 mmol, 0.05 equiv) were placed in a reaction flask. The content of the flask was evacuated and purged with argon. Triethylamine (TEA, 5 mL) was added, and the reaction mixture was stirred for 15 min at 40 °C under the argon atmosphere. Trimethylsilylacetylene (15 μL, 10.0 mg, 0.096 mmol, 1.5 equiv) was added, and the reaction mixture was stirred for 24 h at 40 °C under the argon atmosphere. Distilled water (5 mL) was added, and the crude product was extracted with CH_2_Cl_2_ (3 × 20 mL). Organic layers were combined, washed with 2 M HCl (3 × 15 mL), water, and brine. After drying with MgSO_4_ followed by filtration, volatiles were distilled off on a rotary evaporator. The product was purified using PTLC (SiO_2_; 30% CH_2_Cl_2_/hexane) to provide the target compound **9** as a white solid (19.8 mg, 86%).

^1^H NMR (CDCl_3_, 500 MHz, ppm), δ_H_ 7.18 (s, 1H), 7.13–7.08 (m, 4H), 4.73–4.66 (m, 3H), 3.55 (d, ^2^*J*_H–H_ = 19.9 Hz, 1H), 3.42 (d, ^2^*J*_H–H_ = 19.6 Hz, 1H), 3.41 (d, ^2^*J*_H–H_ = 19.5 Hz, 1H), 0.25 (s, 9H); ^13^C{^1^H} NMR (CDCl_3_, 125 MHz, ppm), δ_C_ 152.0, 149.2 × 2, 149.1 × 2, 149.0, 148.9, 148.8, 148.5 × 2, 148.1, 127.1, 123.9, 123.8, 123.5, 123.4 × 2, 118.0, 104.8, 95.7, 41.9 × 2, 41.7, 0.3 (3C); HRMS (ESI) *m/z* [M]^+^ calcd. For C_26_H_20_Si 360.1334, found 360.1338; *R*_f_ (30% CH_2_Cl_2_/hexane) = 0.60.

##### Step 2: Synthesis of 2-Ethynylsumanene (**6**) from **9**

Trimethyl(sumanenylethynyl)silane (**9**; 16.0 mg, 0.044 mmol, 1 equiv) was placed in the reaction flask. The content of the flask was evacuated and purged with argon. Dry CH_2_Cl_2_ (3 mL) and MeOH (3 mL) were added, followed by the addition of dry potassium carbonate (K_2_CO_3_; 30.0 mg, 0.22 mmol, 5 equiv). The reaction mixture was stirred for 24 h at 27 °C under the argon atmosphere. Distilled water (10 mL) was added, and the crude product was extracted with CH_2_Cl_2_ (3 × 20 mL). Organic layers were combined, washed with water, and brine. After drying with MgSO_4_ followed by filtration, volatiles were distilled off on a rotary evaporator. The product was purified using a PTLC (SiO_2_; 25% CH_2_Cl_2_/cyclohexane) to provide the target compound **6** as a white solid (12.1 mg, 95%), mp = 129–130 °C.

^1^H NMR (CDCl_3_, 500 MHz, ppm), δ_H_ 7.25 (s, 1H), 7.14–7.10 (m, 4H), 4.73 (d, ^2^*J*_H–H_ = 20.0 Hz, 1H), 4.71 (d, ^2^*J*_H–H_ = 19.6 Hz, 1H), 4.70 (d, ^2^*J*_H–H_ = 19.4 Hz, 1H), 3.58 (d, ^2^*J*_H–H_ = 20.0 Hz, 1H), 3.44 (d, ^2^*J*_H–H_ = 19.6 Hz, 1H), 3.43 (d, ^2^*J*_H–H_ = 19.5 Hz, 1H), 3.14 (s, 1H); ^1^H NMR (DMSO-*d*_6_, 500 MHz, ppm), δ_H_ 7.24 (s, 1H), 7.23–7.18 (m, 4H), 4.76–4.68 (m, 3H), 4.28 (s, 1H), 3.55 (d, ^2^*J*_H–H_ = 19.9 Hz, 1H), 3.54 (d, ^2^*J*_H–H_ = 19.7 Hz, 1H), 3.50 (d, ^2^*J*_H–H_ = 19.6 Hz, 1H); ^13^C{^1^H} NMR (CDCl_3_, 125 MHz, ppm), δ_C_ 152.2, 149.3, 149.2 × 3, 149.1, 149.0 × 2, 148.8, 148.4 × 2, 148.0, 127.4, 124.0, 123.9, 123.6, 123.5, 116.7, 83.4, 78.3, 41.9 × 2, 41.8; HRMS (ESI) *m/z* [M]^+^ calcd. For C_23_H_12_ 288.0934, found 288.0935; *R*_f_ (25% CH_2_Cl_2_/cyclohexane) = 0.55.

Note 1: The **6** reaction yield at the larger scale was as follows: Step 1 (0.21 mmol of **2b**)—83% (62.8 mg of the product), Step 2 (0.174 mmol of **9**)—96% (48.2 mg of the product).

#### Synthesis of Ferrocenemethylazide (**10**)

Ferrocenemethylazide (**10**) was synthesized from ferrocenemethylalcohol following a literature procedure.^39^

^1^H NMR (CDCl_3_, 500 MHz, ppm), δ_H_ 4.24–4.23 (t-like m, ^3^*J*_H–H_ = 1.8 Hz, 2 H), 4.21–4.20 (t-like m, ^3^*J*_H–H_ = 1.8 Hz, 2 H), 4.18 (s, 5H), 4.11 (s, 2H).

#### Synthesis of 1-(Ferrocenylmethylmethyl)-4-(4,7-dihydro-1H-tricyclopenta[*def*,*jkl*,*pqr*]triphenylen-2-yl)-1H-1,2,3-triazole (Monoferrocenylsumanene, **7**)

Ferrocenemethylazide (**10**; 16.0 mg, 0.064 mmol, 1.5 equiv), 2-ethynylsumanene (**6**; 12.2 mg, 0.042 mmol, 1 equiv), copper(II) sulfate pentahydrate (CuSO_4_·5H_2_O; 4.1 mg, 0.064 mmol, 0.25 equiv), and sodium ascorbate (6.4 mg, 0.032 mmol, 0.75 equiv) were placed in a reaction flask. DMF (6 mL) was added. The reaction mixture was stirred for 24 h at 55 °C. Distilled water (30 mL) was added, and the formed precipitate was filtrated on a nylon membrane (0.45 μm). The resultant solid was dissolved in CHCl_3_ (45 mL). After drying with MgSO_4_ followed by filtration, volatiles were distilled off on a rotary evaporator. Finally, the product was purified using a PTLC (SiO_2_, 2% CH_3_OH/CHCl_3_) to provide the target monoferrocenylsumanene **7** as a yellow solid (18.7 mg, 80%), mp = 200–201 °C.

^1^H NMR (DMSO-*d*_6_, 500 MHz, ppm), δ_H_ 8.57 (s, 1H), 7.79 (s, 1H), 7.20–7.18 (m, 4H), 5.37 (s, 2H), 4.97 (d, ^2^*J*_H–H_ = 20.5 Hz, 1H), 4.79–4.68 (m, 2H), 4.43–4.41 (m, 2H), 4.21–4.19 (m, 7H), 3.66 (d, ^2^*J*_H–H_ = 20.5 Hz, 1H), 3.57 (d, ^2^*J*_H–H_ = 19.7 Hz, 1H), 3.48 (d, ^2^*J*_H–H_ = 19.7 Hz, 1H); ^13^C{^1^H} NMR (DMSO-*d*_6_, 125 MHz, ppm), δ_C_ 149.6, 149.0, 148.9, 148.8, 148.4, 148.1, 147.9 × 2, 147.6, 147.1, 146.1, 144.2, 126.8, 124.1, 123.8 × 2, 122.1, 120.9, 82.5, 79.2, 79.0, 78.7, 68.7 × 2, 68.6 (5C), 68.3 (2C), 49.1, 42.7, 41.3; HRMS (ESI) *m/z* [M]^+^ calcd. For C_34_H_23_FeN_3_ 529.1241, found 529.1242; UV–Vis, λ_max_ (CHCl_3_, 2 × 10^–5^ M) 290, 334 nm; Emission spectrum (CHCl_3_, 2 × 10^–5^ M, λ_exc_ = 285 nm) 390 nm; *R*_f_ (2% CH_3_OH/CHCl_3_) = 0.40.

## Data Availability

The data underlying this study are available in the published article and its online Supplementary Material.

## Supporting Information Available

Experimental Section; compound characterization data; discussion on the NMR spectra; data on the spectrofluorimetric analyses of the complexes; calculation details; and electrochemical characterization of **7** and **8** (PDF)

## References

[ref1] Sakurai H.; Daiko T.; Hirao T. A Synthesis of Sumanene, a Fullerene Fragment. Science 2003, 301, 1878–1878. 10.1126/science.1088290. 14512619

[ref2] Amaya T.; Hirao T. A Molecular Bowl Sumanene. Chem. Commun. 2011, 47, 10524 10.1039/c1cc12532j. 21743888

[ref3] Sakurai H. The Dawn of Sumanene Chemistry: My Personal History with π-Figuration. Bull. Chem. Soc. Jpn. 2021, 94, 1579–1587. 10.1246/bcsj.20210046.

[ref4] Amaya T.; Seki S.; Moriuchi T.; Nakamoto K.; Nakata T.; Sakane H.; Saeki A.; Tagawa S.; Hirao T. Anisotropic Electron Transport Properties in Sumanene Crystal. J. Am. Chem. Soc. 2009, 131, 408–409. 10.1021/ja805997v. 19105693

[ref5] Saito M.; Shinokubo H.; Sakurai H. Figuration of Bowl-Shaped π-Conjugated Molecules: Properties and Functions. Mater. Chem. Front. 2018, 2, 635–661. 10.1039/C7QM00593H.

[ref6] Alvi S.; Ali R. Synthetic Approaches to Bowl-Shaped π-Conjugated Sumanene and Its Congeners. Beilstein J. Org. Chem. 2020, 16, 2212–2259. 10.3762/bjoc.16.186. 32983269 PMC7492699

[ref7] Amaya T.; Hirao T. Chemistry of Sumanene. Chem. Rec. 2015, 15, 310–321. 10.1002/tcr.201402078. 25474759

[ref8] Shrestha B. B.; Karanjit S.; Panda G.; Higashibayashi S.; Sakurai H. Synthesis of Substituted Sumanenes by Aromatic Electrophilic Substitution Reactions. Chem. Lett. 2013, 42, 386–388. 10.1246/cl.121273.

[ref9] Hisaki I.; Toda H.; Sato H.; Tohnai N.; Sakurai H. A Hydrogen-Bonded Hexagonal Buckybowl Framework. Angew. Chem., Int. Ed. 2017, 56, 15294–15298. 10.1002/anie.201708115. 29024384

[ref10] Toda H.; Yakiyama Y.; Shoji Y.; Ishiwari F.; Fukushima T.; Sakurai H. 2,3,5,6,8,9-Hexabromosumanene: Synthesis and Its Application to Suzuki–Miyaura Cross-Coupling. Chem. Lett. 2017, 46, 1368–1371. 10.1246/cl.170566.

[ref11] Yakiyama Y.; Hasegawa T.; Sakurai H. Formation of a Large Confined Spherical Space with a Small Aperture Using Flexible Hexasubstituted Sumanene. J. Am. Chem. Soc. 2019, 141, 18099–18103. 10.1021/jacs.9b07902. 31608631

[ref12] Shrestha B. B.; Higashibayashi S.; Sakurai H. Columnar/Herringbone Dual Crystal Packing of Pyrenylsumanene and Its Photophysical Properties. Beilstein J. Org. Chem. 2014, 10, 841–847. 10.3762/bjoc.10.80. 24778739 PMC3999760

[ref13] Nakazawa H.; Ohya A.; Morimoto Y.; Uetake Y.; Ikuma N.; Okada K.; Nakano M.; Yakiyama Y.; Sakurai H. Synthesis of Sumanene-fused Acenes. Asian J. Org. Chem. 2022, 11, e202200471 10.1002/ajoc.202200471.

[ref14] Amaya T.; Nakata T.; Hirao T. Synthesis of Highly Strained π-Bowls from Sumanene. J. Am. Chem. Soc. 2009, 131, 10810–10811. 10.1021/ja9031693. 19621924

[ref15] Thirumurugan P.; Matosiuk D.; Jozwiak K. Click Chemistry for Drug Development and Diverse Chemical–Biology Applications. Chem. Rev. 2013, 113, 4905–4979. 10.1021/cr200409f. 23531040

[ref16] Sada K.; Kokado K. Click Chemistry to Metal-Organic Frameworks as a Synthetic Tool for MOF and Applications for Functional Materials. In Advances in Organic Crystal Chemistry; Sakamoto M., Uekusa H., Eds.; Springer Singapore: Singapore, 2020; pp. 523–538.

[ref17] Goto Y.; Sato H.; Shinkai S.; Sada K. “Clickable” Metal–Organic Framework. J. Am. Chem. Soc. 2008, 130, 14354–14355. 10.1021/ja7114053. 18839949

[ref18] Kasprzak A.; Kowalczyk A.; Jagielska A.; Wagner B.; Nowicka A. M.; Sakurai H. Tris(Ferrocenylmethidene)Sumanene: Synthesis, Photophysical Properties and Applications for Efficient Caesium Cation Recognition in Water. Dalton Trans. 2020, 49, 9965–9971. 10.1039/D0DT01506G. 32597432

[ref19] Kasprzak A.; Sakurai H. Site-Selective Cation−π Interaction as a Way of Selective Recognition of the Caesium Cation Using Sumanene-Functionalized Ferrocenes. Dalton Trans. 2019, 48, 17147–17152. 10.1039/C9DT03162F. 31573023

[ref20] Kaeriyama H. Oceanic Dispersion of Fukushima-Derived Radioactive Cesium: A Review. Fish. Oceanogr. 2017, 26, 99–113. 10.1111/fog.12177.

[ref21] Yasunari T. J.; Stohl A.; Hayano R. S.; Burkhart J. F.; Eckhardt S.; Yasunari T. Cesium-137 Deposition and Contamination of Japanese Soils Due to the Fukushima Nuclear Accident. Proc. Natl. Acad. Sci. U. S. A. 2011, 108, 19530–19534. 10.1073/pnas.1112058108. 22084074 PMC3241755

[ref22] Levy I.; Povinec P. P.; Aoyama M.; Hirose K.; Sanchez-Cabeza J. A.; Comanducci J.-F.; Gastaud J.; Eriksson M.; Hamajima Y.; Kim C. S.; Komura K.; Osvath I.; Roos P.; Yim S. A. Marine Anthropogenic Radiotracers in the Southern Hemisphere: New Sampling and Analytical Strategies. Prog. Oceanogr. 2011, 89, 120–133. 10.1016/j.pocean.2010.12.012.

[ref23] Kotilainen A. T.; Kotilainen M. M.; Vartti V.-P.; Hutri K.-L.; Virtasalo J. J. Chernobyl Still with Us: 137Caesium Activity Contents in Seabed Sediments from the Gulf of Bothnia, Northern Baltic Sea. Mar. Pollut. Bull. 2021, 172, 112924 10.1016/j.marpolbul.2021.112924. 34526264

[ref24] Mizuno T.; Kubo H. Overview of Active Cesium Contamination of Freshwater Fish in Fukushima and Eastern Japan. Sci. Rep. 2013, 3, 1742 10.1038/srep01742. 23625055 PMC3638159

[ref25] Lyon A. W.; Mayhew W. J. Cesium Toxicity: A Case of Self-Treatment by Alternate Therapy Gone Awry. Ther. Drug Monitor. 2003, 25, 114–116. 10.1097/00007691-200302000-00018. 12548155

[ref26] Compound **7** was well-soluble in DMSO and DMF whilst sparingly soluble in CHCl_3_ and CH2Cl2 or 50% CHCl_3_/CH_3_OH.

[ref27] Amaya T.; Mori K.; Wu H.-L.; Ishida S.; Nakamura J.; Murata K.; Hirao T. Synthesis and Characterization of π-Extended Bowl-Shaped π-Conjugated Molecules. Chem. Commun. 2007, 19, 1902–1904. 10.1039/B701322A. 17695223

[ref28] Ngamsomprasert N.; Yakiyama Y.; Sakurai H. A Sumanene-Based Aryne, “Sumanyne.”. Chem. Lett. 2017, 46, 446–448. 10.1246/cl.161117.

[ref29] Kasprzak A.; Sakurai H. Disaggregation of a Sumanene-Containing Fluorescent Probe towards Highly Sensitive and Specific Detection of Caesium Cations. Chem. Commun. 2021, 57, 343–346. 10.1039/D0CC07226E. 33319217

[ref30] Kasprzak A.; Tobolska A.; Sakurai H.; Wróblewski W. Tuning the Sumanene Receptor Structure towards the Development of Potentiometric Sensors. Dalton Trans. 2022, 51, 468–472. 10.1039/D1DT03467G. 34904597

[ref31] Spisak S. N.; Wei Z.; Rogachev A. Y.; Amaya T.; Hirao T.; Petrukhina M. A. Double Concave Cesium Encapsulation by Two Charged Sumanenyl Bowls. Angew. Chem., Int. Ed. 2017, 56, 2582–2587. 10.1002/anie.201610696. 28170146

[ref32] Benesi H. A.; Hildebrand J. H. A Spectrophotometric Investigation of the Interaction of Iodine with Aromatic Hydrocarbons. J. Am. Chem. Soc. 1949, 71, 2703–2707. 10.1021/ja01176a030.

[ref33] Goswami S.; Aich K.; Das S.; Das A. K.; Manna A.; Halder S. A Highly Selective and Sensitive Probe for Colorimetric and Fluorogenic Detection of Cd2+ in Aqueous Media. Analyst 2013, 138, 1903 10.1039/c3an36884j. 23392200

[ref34] Priyakumar U. D.; Sastry G. N. Cation-π Interactions of Curved Polycyclic Systems: M+ (M=Li and Na) Ion Complexation with Buckybowls. Tetrahedron Lett. 2003, 44, 6043–6046. 10.1016/S0040-4039(03)01512-0.

[ref35] Vijay D.; Sakurai H.; Subramanian V.; Sastry G. N. Where to Bind in Buckybowls? The Dilemma of a Metal Ion. Phys. Chem. Chem. Phys. 2012, 14, 3057 10.1039/c2cp22087c. 22278659

[ref36] Tsierkezos N. G. Cyclic Voltammetric Studies of Ferrocene in Nonaqueous Solvents in the Temperature Range from 248.15 to 298.15 K. J. Solution Chem. 2007, 36, 289–302. 10.1007/s10953-006-9119-9.

[ref37] Lewandowski A.; Waligora L.; Galinski M. Ferrocene as a Reference Redox Couple for Aprotic Ionic Liquids. Electroanalysis 2009, 21, 2221–2227. 10.1002/elan.200904669.

[ref38] Neghmouche N.; Khelef A.; Lanez T. Electrochemistry Characterization of Ferrocene/Ferricenium Redox Couple at Glassycarbon Electrode. J. Fundam. Appl Sci. 2015, 1, 23 10.4314/jfas.v1i2.3.

[ref39] Casas-Solvas J. M.; Ortiz-Salmerón E.; Giménez-Martínez J. J.; García-Fuentes L.; Capitán-Vallvey L. F.; Santoyo-González F.; Vargas-Berenguel A. Ferrocene-Carbohydrate Conjugates as Electrochemical Probes for Molecular Recognition Studies. Chem. – Eur. J. 2009, 15, 710–725. 10.1002/chem.200800927. 19053085

