# Supplementary material for: Application of Monoferrocenylsumanenes Derived from Sonogashira Cross-Coupling or Click Chemistry Reactions in Highly Sensitive and Selective Cesium Cation Electrochemical Sensors
Source: J Org Chem. 2023 Mar 14;88(7):4199–208. doi: 10.1021/acs.joc.2c02767 (PMC10088032; doi:10.1021/acs.joc.2c02767)
Supplement: Supplementary file 1 — jo2c02767_si_001.pdf [file jo2c02767_si_001.pdf]

Application of monoferrocenylsumanenes derived from Sonogashira cross-coupling or *click chemistry* reactions in highly sensitive and selective cesium cations electrochemical sensors

SUPPORTING INFORMATION

Artur Kasprzak[a]*, Aleksandra Gajda-Walczak[b], Agata Kowalczyk[b], Barbara Wagner[c], Anna M. Nowicka[b], Mikey Nishimoto[d], Mariola Koszytkowska-Stawińska[a], Hidehiro Sakurai[d,e]

[a] Faculty of Chemistry, Warsaw University of Technology, Noakowskiego Str. 3, 00-664 Warsaw, Poland

* Corresponding author e-mail: artur.kasprzak@pw.edu.pl (A.K.)

[b] Faculty of Chemistry, University of Warsaw, Pasteura Str. 1, 02-093 Warsaw, Poland

[c] Biological and Chemical Research Centre, Faculty of Chemistry, University of Warsaw, Zwirki i Wigury Str. 101, PL-02-093 Warsaw, Poland

[d] Division of Applied Chemistry, Graduate School of Engineering, Osaka University, 2-1 Yamadaoka, Suita, 565-0871 Osaka, Japan

[e] Innovative Catalysis Science Division, Institute for Open and Transdisciplinary Research Initiatives (ICS-OTRI), Osaka University, Suita, Osaka 565-0871, Japan

Table of contents

List of Figures.......... S3

1. Experimental section.......... S6

   1.1. Materials and methods.......... S6

   1.2. Synthesis of 4,7-dihydro-1H-tricyclopenta[*def*,*jkl*,*pqr*]triphenylene (sumanene; **1**). S7

   1.3. Synthesis of 2-bromo-4,7-dihydro-1H-tricyclopenta[*def*,*jkl*,*pqr*]triphenylene (2-bromosumanene, **2a**).......... S7

   1.4. Synthesis of 2-iodo-4,7-dihydro-1H-tricyclopenta[*def*,*jkl*,*pqr*]triphenylene (2-iodosumanene, **2b**).......... S8

   1.5. Synthesis of 2-(ferrocenylethynyl)-4,7-dihydro-1H-tricyclopenta[*def*,*jkl*,*pqr*]triphenylene (monoferrocenylsumanene, **8**).......... S8

   1.6. Synthesis of 2-ethynyl-4,7-dihydro-1H-tricyclopenta[*def*,*jkl*,*pqr*]triphenylene (2-ethynylsumanene, **6**).......... S9

   1.7. Synthesis of ferrocenemethylazide (**10**).......... S10

   1.8. Synthesis of 1-(ferrocenylmethylmethyl)-4-(4,7-dihydro-1H-tricyclopenta[*def*,*jkl*,*pqr*]triphenylen-2-yl)-1H-1,2,3-triazole (monoferrocenylsumanene, **7**).......... S10

   1.9. Preparation of the cesium cations electrochemical sensor.......... S11

2. NMR spectra.......... S12

3. Explanation of the specific ^{1}H NMR profiles of compounds **2a**, **2b** and **6-8**.......... S43

4. Calculations.......... S46

5. Spectrofluorimetric analyzes of the interactions between monoferrocenylsumanene **7** or **8** and cesium cations.......... S57

6. Electrochemical characterization of monoferrocenylsumanenes **7** and **8**.......... S62

7. Additional data on LA-ICP-MS.......... S64

8. Supporting references.......... S66

List of Figures

Figure S1. ^{1}H NMR (500 MHz, $CDCl_3$) spectrum of sumanene (**1**). S12
Figure S2. ^{1}H NMR (500 MHz, DMSO-d_6) spectrum of sumanene (**1**) S13
Figure S3. ^{1}H NMR (500 MHz, $CDCl_3$) spectrum of 2-bromosumanene (**2a**). S14
Figure S4. $^{13}C\{^{1}H\}$ NMR (125 MHz, $CDCl_3$) spectrum of 2-bromosumanene (**2a**). The unlabeled peaks come from the traces of dibromosumanenes. S15
Figure S5. ^{1}H-^{13}C HSQC NMR ($CDCl_3$) spectrum of 2-bromosumanene (**2a**). S16
Figure S6. ^{1}H-^{13}C HMBC NMR ($CDCl_3$) spectrum of 2-bromosumanene (**2a**). S17
Figure S7. ^{1}H NMR (500 MHz, $CDCl_3$) spectrum of 2-iodosumanene (**2b**). S18
Figure S8. $^{13}C\{^{1}H\}$ NMR (125 MHz, $CDCl_3$) spectrum of 2-iodosumanene (**2b**). S19
Figure S9. ^{1}H-^{13}C HSQC NMR ($CDCl_3$) spectrum of 2-iodosumanene (**2b**) S20
Figure S10. ^{1}H-^{13}C HMBC NMR ($CDCl_3$) spectrum of 2-iodosumanene (**2b**). S21
Figure S11. ^{1}H NMR (500 MHz, $CDCl_3$) spectrum of 2-ethynylsumanene (**6**). The distinctive singlet at 3.14 ppm (^{1}H NMR, $CDCl_3$; the C(*sp*)-H) and two signals at 83.4 ppm and 78.3 ppm ($\{^{1}H\}^{13}C$ NMR, $CDCl_3$; the C(*sp*)) confirmed the presence of the acetylene group. The ^{1}H NMR spectrum also comprised the signals characteristic for the sumanene benzylic H_{endo} protons (3.60-3.41 ppm), benzylic H_{exo} protons (4.75-4.68 ppm), and aromatic protons (7.21-7.11 ppm). S22
Figure S12. $^{13}C\{^{1}H\}$ NMR (125 MHz, $CDCl_3$) spectrum of 2-ethynylsumanene (**6**). S23
Figure S13. ^{1}H-^{13}C HSQC NMR ($CDCl_3$) spectrum of 2-ethynylsumanene (**6**). S24
Figure S14. ^{1}H-^{13}C HMBC NMR ($CDCl_3$) spectrum of 2-ethynylsumanene (**6**). S25
Figure S15. ^{1}H-^{13}C HMBC NMR ($CDCl_3$) spectrum of 2-ethynylsumanene (**6**) - inset. S26
Figure S16. ^{1}H-^{13}C HMBC NMR ($CDCl_3$) spectrum of 2-ethynylsumanene (**6**) - inset. S27
Figure S17. ^{1}H NMR (500 MHz, DMSO-d_6) spectrum of monoferrocenylsumanene **7**. S28
Figure S18. $^{13}C\{^{1}H\}$ NMR (125 MHz, DMSO-d_6) spectrum of monoferrocenylsumanene **7**. S29
Figure S19. Comparison of ^{1}H NMR spectra (500 MHz, DMSO-d_6) 2-ethynylsumanene (**6**), and monoferrocenylsumanene **7**. Selected insets of the spectra are presented. S30
Figure S20. ^{1}H-^{13}C HSQC NMR (DMSO-d_6) spectrum of monoferrocenylsumanene **7**. S31
Figure S21. ^{1}H-^{13}C HMBC NMR (DMSO-d_6) spectrum of monoferrocenylsumanene **7**. S32
Figure S22. ^{1}H-^{13}C HMBC NMR (DMSO-d_6) spectrum of monoferrocenylsumanene **7** – insets. S33
Figure S23. ^{1}H NMR (500 MHz, $CDCl_3$) spectrum of monoferrocenylsumanene **8**. S34
Figure S24. $^{13}C\{^{1}H\}$ NMR (125 MHz, $CDCl_3$) spectrum of monoferrocenylsumanene **8**. S35
Figure S25. Comparison of ^{1}H NMR spectra (500 MHz) of 1,4-diferrocenylbuta-1,3-diyne (**12**; brown spectrum), the product mixture from the first PTLC purification (green spectrum), and the pure **8** (from the second PTLC purification; blue spectrum). The inset of ESI-HRMS spectrum of the green spectrum sample is also presented. Labels of signals are also presented. The same color does not correspond to the same chemical shift. Selected insets of the spectra are presented. The signals of impurity **12** were clearly seen in the ^{1}H NMR spectrum of the sample obtained from the first PTLC purification process (eluent: 25% CH_2Cl_2/hexane) and the presence of residual **12** in that sample was also detected with high-resolution mass. The second PTLC purification (eluent: 50% THF/hexane) yielded pure **8**. S36
Figure S26. The selected insets of the ^{1}H NMR spectra (500 MHz, $CDCl_3$; 2-iodosumanene **2b** - top and monoferrocenylsumanene **8** - bottom). The signals of the ferrocene protons are marked with the yellow frame. In a relation to the ^{1}H NMR spectrum of compound **2b**, the spectrum of compound **8** showed: (a) signals of the ferrocene protons at 4.52-4.51 ppm and 4.26-4.25 ppm (multiplets, protons from the monosubstituted cyclopentadienyl (Cp) ring), and at 4.24 ppm (singlet, protons from the unsubstituted Cp ring), (b) signal of the sumanene

aromatic proton from the substituted ring (brown) less separated from the signals of aromatic protons from the unsubstituted ring (violet), (c) downfield-shifted signals of the benzylic H_{exo} protons (light green) and benzylic H_{endo} protons (dark green). The linking of sumanene **1** with the 2-ferroceneethynyl moiety was also evidenced with the $^{13}C\{^{1}H\}$ NMR spectrum by signals at 89.8 ppm (1xC), 85.4 ppm (1xC), 71.6 ppm (2xC), 70.2 ppm (5xC), 69.0 ppm (2xC) and 65.6 ppm (1xC). S37
Figure S27. ^{1}H-^{13}C HSQC NMR ($CDCl_3$) spectrum of monoferrocenylsumanene **8**........ S38
Figure S28. ^{1}H-^{13}C HMBC NMR ($CDCl_3$) spectrum of monoferrocenylsumanene **8**. S39
Figure S29. ^{1}H-^{13}C HMBC NMR ($CDCl_3$) spectrum of monoferrocenylsumanene **8** – insets. S40
Figure S30. ^{1}H NMR (500 MHz, $CDCl_3$) spectrum of compound **9**. S41
Figure S31. $^{13}C\{^{1}H\}$ NMR (125 MHz, $CDCl_3$) spectrum of compound **9**. S42
Figure S32. DFT-optimized structure of the compound **6** viewed from two different perspectives. S52
Figure S33. DFT-optimized structure of the monoferrocenylsumanene **8** viewed from two different perspectives. S52
Figure S34. DFT-optimized structure of the monoferrocenylsumanene **7** viewed from two different perspectives. S53
Figure S35. Calculated energy level and Kohn–Sham orbitals at the HOMO and LUMO of **1**, **7**, and **8**. S54
Figure S36. Calculated electrostatic potential (ESP) of a) **1**, b) **7**, and c) **8** from concave face of sumanene bowl**.** Isosurface for electrostatic potential was 0.002 au. The calculation was performed at the ωB97X-D/Def2-SVP level. S55
Figure S37. (**a**) Method of defining the bowl depth. (**b**) Bowl depth for sumanene (**1**). (**c**) Structures of sumanene derivatives **6**, **7** and **8** together with their DFT optimized structures (the sumanene bowl is marked yellow) and bowl depths (black arrow denotes the carbon atom for which bowl depth was taken) at the ωB97X-D/Def2-SVP level of theory. The bowl depth for sumanene (**1**) was estimated based on the DFT-optimized structure. S56
Figure S38. Comparison between emission spectra (λ_{ex} = 285 nm) of **8** in $CHCl_3$ and $CHCl_3$:CH_3OH (1/1 *v*/*v*). S57
Figure S39. Emission spectra (λ_{ex} = 285 nm) of **8** in the presence of various amounts (equivalents = eq) of cesium cations. S58
Figure S40. Job's plot regarding the interactions between monoferrocenylsumanene **8** and cesium cations (*x* stands for the molar fraction of **8**; I_0 and I are the fluorescence intensities of **8** in the absence and in the presence of cesium cations). S58
Figure S41. Benesi-Hildebrand plot regarding the interactions between **8** and cesium cations (*C* stands for the molar concentration of cesium cations in the sample; I_0 and I are the fluorescence intensities of **8** in the absence and in the presence of cesium cations). The linear fit data and the calculated K_{app} are also presented. S59
Figure S42. Emission spectra (λ_{ex} = 285 nm) of **8** in the absence or in the presence of various cations (10 eq). S59
Figure S43. Comparison between emission spectra (λ_{ex} = 285 nm) of **7** in $CHCl_3$ and $CHCl_3$:CH_3OH (1/1 *v*/*v*). S60
Figure S44. Emission spectra (λ_{ex} = 285 nm) of **7** in the presence of various amounts (equivalents = eq) of cesium cations. S60
Figure S45. Job's plot regarding the interactions between **7** and cesium cations (*x* stands for the molar fraction of **7**; I_0 and I are the fluorescence intensities of **7** in the absence and in the presence of cesium cations). S61
Figure S46. Benesi-Hildebrand plot regarding the interactions between monoferrocenylsumanene **7** and cesium cations (*C* stands for the molar concentration of cesium cations in the sample; I_0 and I are the fluorescence intensities of **7** in the absence and

in the presence of cesium cations). The linear fit data and the calculated K_{app} are also presented.. S61
Figure S47. Cyclic voltammograms (plotted in IUPAC convention) of monoferrocenylsumanenes **7** (A) and monoferrocenylsumanene **8** (B) recorded in DCM. Insets: Dependencies of anodic peak currents *vs.* square root of scan rate. Experimental conditions: solvent: dichloromethane (DCM), supporting electrolyte: tetrabutylammonium hexafluorophosphate ($TBAPF_6$), $C_{compound}$ = 0.02 mM, C_{TBAPF6} = 100 mM, T = 21 °C, working electrode: disc glassy electrode (ϕ = 3 mm), counter electrode: Pt plate (A = *ca.* 1 cm^2, reference electrode: Ag/AgCl/3 M KCl. S63
Figure S48. Cyclic voltammograms (plotted in IUPAC convention) of monoferrocenylsumanenes **7** (A) and monoferrocenylsumanene **8** (B) recorded in DCM. Insets: Dependencies of anodic peak currents *vs.* square root of scan rate. Experimental conditions: solvent: dichloromethane (DCM), supporting electrolyte: tetrabutylammonium hexafluorophosphate ($TBAPF_6$), $C_{compound}$ = 0.02 mM, C_{TBAPF6} = 100 mM, T = 21 °C, working electrode: disc glassy electrode (ϕ = 3 mm), counter electrode: Pt plate (A = *ca.* 1 cm^2, reference electrode: Ag/AgCl/3 M KCl. S64

1. **Experimental section**

1.1. Materials and methods

Chemical reagents and solvents for the synthesis were commercially purchased and purified according to the standard methods, if necessary. Thin layer chromatography (TLC) and preparative thin layer chromatography (PTLC) were performed using Merck Silica gel 60 F254 plates.

The NMR experiments were carried out using a Varian VNMRS 500 MHz spectrometer (^{1}H NMR at 500 MHz, ^{13}C{^{1}H} NMR at 125 MHz) equipped with a multinuclear z-gradient inverse probe head. The spectra were recorded at 25 °C and standard 5 mm NMR tubes were used. ^{1}H chemical shifts (δ) were reported in parts per million (ppm) relative to the solvent signal, *i.e.*, $CDCl_3$: δ_H (residual $CHCl_3$) 7.26 ppm, δ_C (residual $CHCl_3$) 77.2 ppm; DMSO-d_6: δ_H (residual DMSO) 2.50 ppm, δ_C (residual DMSO) 39.5 ppm. Spin-spin coupling constant values (*J*) were given in Hz and were calculated using the Resolution Booster processing mode. NMR spectra were analyzed with the MestReNova v12.0 software (Mestrelab Research S.L).

ESI-HRMS (TOF) measurements were performed with a Q-Exactive ThermoScientific spectrometer.

UV-vis measurements were performed with the PerkinElmer spectrometer Lambda 25, at room temperature in quartz cuvette of 1 cm length of optical window. For the UV-Vis measurements, the wavelengths for the absorption maxima λ_{max} were reported in nm. Spectrofluorimetric analyzes were performed with a Hitachi F-4500 fluorescence spectrophotometer with the spectral resolution of 1 nm, the wavelengths for the emission maxima were reported in nm.

Cyclic voltammetry (CV) and differential pulse voltammetry (DPV) experiments were performed in the three-electrode system with using a an Autolab potentiostat, model PGSTAT 12. The disc glassy carbon electrode (GC; ϕ = 3 mm) was used as a working electrode, the Ag/AgCl/3 M KCl as a reference electrode and the platinum plate with an area of at least 1 cm^2 as a counter electrode. To minimize the electrical noise all experiments were carried out in Faraday cage. The electrochemical characteristic of the studied ferrocene derivatives (compound 7 and compound 8) was done in the dichloromethane (DCM) with addition of tetrabutylammonium hexafluorophosphate ($TBAPF_6$) as a supporting electrolyte. The concentration of the studied ferrocene derivatives was 0.02 mM. Dry dichlorometane (DCM, Sigma-Aldrich), tetrabutylammonium hexafluorophosphate ($TBAPF_6$, Sigma-Aldrich), tetrabutylammonium tetrafluoroborate ($TBABF_4$, Sigma-Aldrich), cesium nitrate ($CsNO_3$, Sigma-Aldrich), potassium nitrate (KNO_3, Sigma-Aldrich), sodium nitrate ($NaNO_3$, Sigma-Aldrich), barium nitrate ($Ba(NO_3)_2$, Sigma-Aldrich) and perfluorinated resin solution containing Nafion™ (nafion™, Sigma-Aldrich) were used in electrochemical studies without additional purification.

The experimental data for LA-ICP-MS (Laser Ablation Inductively Coupled Plasma Mass Spectrometry) experiments were as follows. The Nd:YAG laser ablation system (LSX-213, CETAC, USA) was coupled to ICP-MS mass spectrometer (NexION 300D, Perkin Elmer, USA). The laser beam wavelength of λ = 213 nm, energy of 3mJ and diameter of 100 µm was used to ablate the surface layers of the analysed samples. During a multi-line ablation (n=5, 20Hz) with the constant scan rate of 100 µm/s transient signals were registered for ^{23}Na, ^{39}K, ^{57}Fe, ^{133}Cs and ^{137}Ba. The operating

conditions of the used ICP-MS system are given in **Table S1**. For each isotope raw signals were individually background corrected for Ar flow before the start of ablation. Spikes were defined as a single data point exceeding the intensities of the neighbouring data for more than 2 times. They were replaced with the average value calculated based on the intensities of two neighbouring signals. All recalculations were done with the use of a custom written formula in Excel® (Microsoft Corp.).

Table S1. Optimized parameters of ICP-MS.

**Parameters :**	
Plasma power	1400 W
Carrier gas flow (Ar)	0.88 L/min
Sweeps	1
Readings	24321
Repeats	1
Dwell time	1 ms

1.2. Synthesis of 4,7-dihydro-1H-tricyclopenta[*def*,*jkl*,*pqr*]triphenylene (sumanene; 1)

Sumanene (**1**) was synthesized following a literature procedure.[1]

^{1}H NMR ($CDCl_3$, 500 MHz, ppm), δ_H 7.10 (s, 6H), 4.71 (d, $^2J_{H\text{-}H}$ = 18.1 Hz, 3H), 3.42 (d, $^2J_{H\text{-}H}$ = 18.1 Hz, 3H); ^{1}H NMR ($CDCl_3$, 500 MHz, ppm), δ_H 7.18 (s, 1H), 4.70 (d, $^2J_{H\text{-}H}$ = 18.3 Hz, 3H), 3.50 (d, $^2J_{H\text{-}H}$ = 18.3 Hz, 3H).

1.3. Synthesis of 2-bromo-4,7-dihydro-1H-tricyclopenta[*def*,*jkl*,*pqr*]triphenylene (2-bromosumanene, 2a)

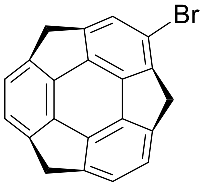

2-Bromosumanene (**2a**) was synthesized from sumanene (**1**) following a literature procedure[2], with slight modification in reaction time. To a stirred solution of sumanene (50.0 mg, 0.188 mmol, 1 eq) in CH_2Cl_2 (17 mL), a solution of pyridinium hydrobromide perbromide (120.0 mg, 0.377 mmol, 1.8 eq) in CH_3CN (8.5 mL) was added dropwise at 0°C. The reaction mixture was then stirred at 27°C for 4 hours. The reaction was quenched with sat. $Na_2S_2O_3$ (7 mL) and sat. $NaHCO_3$ (7 mL), and the crude product was extracted with CH_2Cl_2 (3x30 mL). Organic layers were combined, washed with water and brine. After drying with $MgSO_4$ followed by filtration, volatiles were distilled off on a rotary evaporator. The product was purified using a preparative thin layer chromatography (PTLC; SiO_2, 25% hex/ CH_2Cl_2), to provide the 2-bromosumanene (**2a**) as a white solid (61.3 mg, 95%).

^{1}H NMR ($CDCl_3$, 500 MHz, ppm), δ_H 7.21 (s, 1H), 7.17-7.10 (m, 4H), 4.72-4.65 (m, 3H), 3.50 (d, $^2J_{H\text{-}H}$ = 19.6 Hz, 1H), 3.44 (d, $^2J_{H\text{-}H}$ = 19.5 Hz, 2H); ^{13}C{^{1}H} NMR ($CDCl_3$, 125

MHz, ppm), δ_C 151.8, 149.3x2, 149.2, 149.0, 148.9, 148.7, 148.4x2, 148.2, 147.9, 147.4, 127.0, 124.2, 123.7, 123.6, 123.5, 116.7, 43.3, 41.9, 41.7.

1.4. Synthesis of 2-iodo-4,7-dihydro-1H-tricyclopenta[*def,jkl,pqr*]triphenylene (2-iodosumanene, 2b)

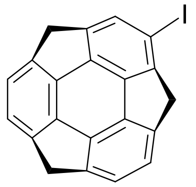

2-Iodosumanene (**2b**) was synthesized from sumanene (**1**) following a literature procedure[3], using 1,3-diiodo-5,5-dimethylhydantoin (DIH) as the iodination reagent with the catalytic amount of trifluoroacetic acid (TFA).

^{1}H NMR ($CDCl_3$, 500 MHz, ppm), δ_H 7.43 (s, 1H), 7.17-7.09 (m, 4H), 4.73-4.66 (m, 3H), 3.43 (d, $^2J_{H\text{-}H}$ = 19.6 Hz, 1H), 3.42 (d, $^2J_{H\text{-}H}$ = 19.6 Hz, 1H), 3.34 (d, $^2J_{H\text{-}H}$ = 19.6 Hz, 1H).

1.5. Synthesis of 2-(ferrocenylethynyl)-4,7-dihydro-1H-tricyclopenta[*def,jkl,pqr*]triphenylene (monoferrocenylsumanene, 8)

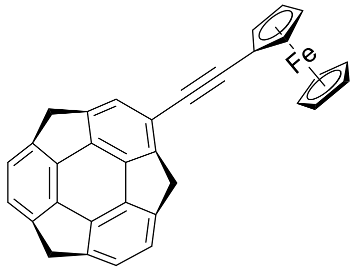

2-Iodosumanene (**2b**; 14.4 mg, 0.04 mmol, 1 eq), bis(triphenylphosphine)palladium(II) dichloride ($Pd(PPh_3)_2Cl_2$; 2.6 mg, 0.0004 mmol, 0.1 eq) and copper(I) iodide (CuI; 0.4 mg, 0.002 mmol, 0.05 eq) were placed in a reaction flask. The content of the flask was evacuated and purged with argon. Triethylamine (TEA, 2.5 mL) was added, and the reaction mixture was stirred for 15 min at 50°C under argon atmosphere. A solution of ethynylferrocene (**11**; 11.6 mg, 0.06 mmol, 1.5 eq) in TEA (1.5 mL) was added, and the reaction mixture was stirred for 24 hours at 50°C under argon atmosphere. Distilled water (6 mL) was added, and the crude product was extracted with CH_2Cl_2 (3x20 mL). Organic layers were combined, washed with 2M HCl (3x15 mL), water, and brine. After drying with $MgSO_4$ followed by filtration, volatiles were distilled off on a rotary evaporator. The product was purified using a two-step PTLC purification (SiO_2; 1st purification: 25% CH_2Cl_2/hexane, 2nd purification: 50% THF/hexane) to provide the target monoferrocenylsumanene **8** as a yellow solid (14.8 mg, 78%), mp = 190−192 °C.

^{1}H NMR ($CDCl_3$, 500 MHz, ppm), δ_H 7.22 (1, 1H), 7.15-7.11 (m, 4H), 4.79-4.69 (m, 3H), 4.52-4.51 (m, 2H), 4.26-4.25 (t-like m, $^3J_{H\text{-}H}$ = 1.8 Hz, 2H), 4.24 (s, 5H), 3.60 (d, $^2J_{H\text{-}H}$ = 19.7 Hz, 1H), 3.44 (d, $^2J_{H\text{-}H}$ = 19.4 Hz, 1H), 3.43 (d, $^2J_{H\text{-}H}$ = 19.3 Hz, 1H); $^{13}C\{^1H\}$ NMR ($CDCl_3$, 125 MHz, ppm), δ_C 150.7, 149.4, 149.2, 149.1, 149.0x2 (3C), 148.9, 148.8, 148.6, 148.5, 148.2x2, 126.7, 123.8, 123.7, 123.5x2, 118.8, 89.8, 85.4, 71.6 (2C), 70.2 (5C), 69.0. 65.6 (2C), 42.0, 41.9, 41.7; HRMS (ESI) *m/z* $[M]^+$ calcd. for $C_{33}H_{20}Fe$ 472.0909, found 472.0910; UV-Vis, λ_{max} ($CHCl_3$, $2{\cdot}10^{-5}$ M) 289, 330 nm; Emission spectrum ($CHCl_3$, $2{\cdot}10^{-5}$ M, λ_{exc} = 285 nm) 402 nm; R_f (50% THF/hexane) = 0.85.

*Note 1: The major impurity in that reaction was 1,4-diferrocenylbuta-1,3-diyne (**12**; R_f (50% THF/hexane) = 0.88; a side product of Glaser coupling between two molecules of ethynylferrocene (**11**)). After the first PTLC separation (25% CH_2Cl_2/hexane), the presence of this side product was observed in the crude mixture. Second PTLC separation (50% THF/hexane) enabled the isolation of the pure target product **8**.*

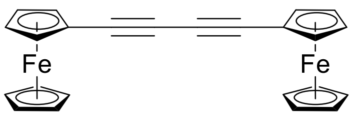

12

*Note 2: The formation of side products, namely di-(ferrocenylethynyl)sumanenes (ca. 15 wt% of the mass of the crude mixture), was observed when 2-iodosumanene sample that was prepared from sumanene (**1**) using the different method (employing gold(III) chloride and N-iodosuccinimide)[4], was used in the Sonogashira cross-coupling reaction with ethynylferrocene (**11**). Those side products could be removed from the crude product using gel permeation chromatography (GPC; $CHCl_3$), however, yield of **8** in that synthesis was lower (ca. 65%).*

*Note 3: The **8** reaction yield starting from 2-bromosumanene (**2a**) is 37%.*

1.6. Synthesis of 2-ethynyl-4,7-dihydro-1H-tricyclopenta[*def*,*jkl*,*pqr*]triphenylene (2-ethynylsumanene, 6)

2-Ethynylsumanene (**6**) was synthesized in two steps.

Step 1: Synthesis of ((4,7-dihydro-1H-tricyclopenta[*def*,*jkl*,*pqr*]triphenylen-2-yl)ethynyl)trimethylsilane (trimethyl(sumanenylethynyl)silane, **9**) from **2b**.

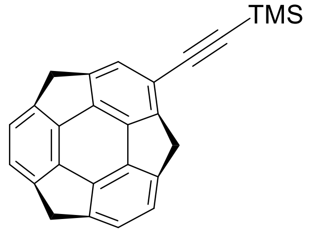

2-Iodosumanene (**2b**; 25.0 mg, 0.064 mmol, 1 eq), $Pd(PPh_3)_2Cl_2$ (4.5 mg, 0.0064 mmol, 0.1 eq) and CuI (1.0 mg, 0.0032 mmol, 0.05 eq) were placed in a reaction flask. The content of the flask was evacuated and purged with argon. Triethylamine (TEA, 5 mL) was added, and the reaction mixture was stirred for 15 min at 40°C under argon atmosphere. The trimethylsilylacetylene (15 µL, 10.0 mg, 0.096 mmol, 1.5 eq) was added, and the reaction mixture was stirred for 24 hours at 40°C under argon atmosphere. Distilled water (5 mL) was added, and the crude product was extracted with CH_2Cl_2 (3x20 mL). Organic layers were combined, washed with 2M HCl (3x15 mL), water, and brine. After drying with $MgSO_4$ followed by filtration, volatiles were distilled off on a rotary evaporator. The product was purified using a PTLC (SiO_2; 30% CH_2Cl_2/hexane) to provide the target compound **9** as a white solid (19.8 mg, 86%).

^{1}H NMR ($CDCl_3$, 500 MHz, ppm), δ_H 7.18 (s, 1H), 7.13–7.08 (m, 4H), 4.73-4.66 (m, 3H), 3.55 (d, $^2J_{H-H}$ = 19.9 Hz, 1H), 3.42 (d, $^2J_{H-H}$ = 19.6 Hz, 1H), 3.41 (d, $^2J_{H-H}$ = 19.5 Hz, 1H), 0.25 (s, 9H); $^{13}C\{^1H\}$ NMR ($CDCl_3$, 125 MHz, ppm), δ_C 152.0, 149.2x2, 149.1x2, 149.0, 148.9, 148.8, 148.5x2, 148.1, 127.1, 123.9, 123.8, 123.5, 123.4x2, 118.0, 104.8, 95.7, 41.9x2, 41.7, 0.3 (3C); HRMS (ESI) *m/z* $[M]^+$ calcd. for $C_{26}H_{20}Si$ 360.1334, found 360.1338; R_f (30% CH_2Cl_2/hexane) = 0.60.

Step 2: Synthesis of 2-ethynylsumanene (**6**) from **9**.

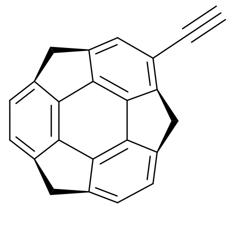

Trimethyl(sumanenylethynyl)silane (***9***; 16.0 mg, 0.044 mmol, 1 eq) was placed in the reaction flask. The content of the flask was evacuated and purged with argon. Dry CH_2Cl_2 (3 mL) and MeOH (3 mL) were added, followed be the addition of dry potassium carbonate (K_2CO_3; 30.0 mg, 0.22 mmol, 5 eq). The reaction mixture was stirred for 24 hours at 27°C under argon atmosphere. Distilled water (10 mL)

was added, and the crude product was extracted with CH_2Cl_2 (3x20 mL). Organic layers were combined, washed with water, and brine. After drying with $MgSO_4$ followed by filtration, volatiles were distilled off on a rotary evaporator. The product was purified using a PTLC (SiO_2; 25% CH_2Cl_2/cyclohexane) to provide the target compound **6** as a white solid (12.1 mg, 95%), mp = 129−130 °C.

^{1}H NMR ($CDCl_3$, 500 MHz, ppm), δ_H 7.25 (s, 1H), 7.14–7.10 (m, 4H), 4.73 (d, $^2J_{H-H}$ = 20.0 Hz, 1H), 4.71 (d, $^2J_{H-H}$ = 19.6 Hz, 1H), 4.70 (d, $^2J_{H-H}$ = 19.4 Hz, 1H), 3.58 (d, $^2J_{H-H}$ = 20.0 Hz, 1H), 3.44 (d, $^2J_{H-H}$ = 19.6 Hz, 1H), 3.43 (d, $^2J_{H-H}$ = 19.5 Hz, 1H), 3.14 (s, 1H); ^{1}H NMR (DMSO-d_6, 500 MHz, ppm), δ_H 7.24 (s, 1H), 7.23–7.18 (m, 4H), 4.76-4.68 (m, 3H), 4.28 (s, 1H), 3.55 (d, $^2J_{H-H}$ = 19.9 Hz, 1H), 3.54 (d, $^2J_{H-H}$ = 19.7 Hz, 1H), 3.50 (d, $^2J_{H-H}$ = 19.6 Hz, 1H); ^{13}C{^{1}H} NMR ($CDCl_3$, 125 MHz, ppm), δ_C 152.2, 149.3, 149.2x3, 149.1, 149.0x2, 148.8, 148.4x2, 148.0, 127.4, 124.0, 123.9, 123.6, 123.5, 116.7, 83.4, 78.3, 41.9x2, 41.8; HRMS (ESI) *m/z* $[M]^+$ calcd. for $C_{23}H_{12}$ 288.0934, found 288.0935; R_f (25% CH_2Cl_2/cyclohexane) = 0.55.

*Note 1: The **6** reaction yield at the larger scale was as follows: Step 1 (0.21 mmol of **2b**) - 83% (62.8 mg of the product), Step 2 (0.174 mmol of **9**) - 96% (48.2 mg of the product).*

1.7. Synthesis of ferrocenemethylazide (10)

Ferrocenemethylazide (**10**) was synthesized from ferrocenemethylalcohol following a literature procedure.[5]

^{1}H NMR ($CDCl_3$, 500 MHz, ppm), δ_H 4.24-4.23 (t-like m, $^3J_{H-H}$ = 1.8 Hz, 2 H), 4.21-4.20 (t-like m, $^3J_{H-H}$ = 1.8 Hz, 2 H), 4.18 (s, 5H), 4.11 (s, 2H).

1.8. Synthesis of 1-(ferrocenylmethylmethyl)-4-(4,7-dihydro-1H-tricyclopenta[*def,jkl,pqr*]triphenylen-2-yl)-1H-1,2,3-triazole (monoferrocenylsumanene, 7)

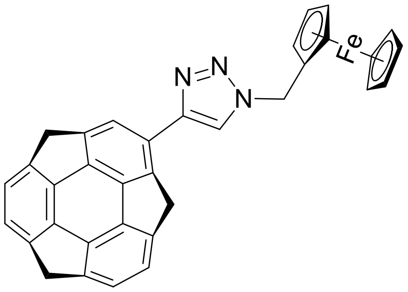

Ferrocenemethylazide (**10**; 16.0 mg, 0.064 mmol, 1.5 eq), 2-ethynylsumanene (**6**; 12.2 mg, 0.042 mmol, 1 eq), copper(II) sulphate pentahydrate ($CuSO_4·5H_2O$; 4.1 mg, 0.064 mmol, 0.25 eq) and sodium ascorbate (6.4 mg, 0.032 mmol, 0.75 eq) were placed in a reaction flask. DMF (6 mL) was added. The reaction mixture was stirred for 24 hours at 55°C. Distilled water (30 mL) was added, and the formed precipitate was filtrated on a nylon membrane (0.45 µm). The resultant solid was dissolved in $CHCl_3$ (45 mL). After drying with $MgSO_4$ followed by filtration, volatiles were distilled off on a rotary evaporator. Finally, the product was purified using a PTLC (SiO_2, 2% $CH_3OH/CHCl_3$) to provide the target monoferrocenylsumanene **7** as a yellow solid (18.7 mg, 80%), mp = 200−201 °C.

^{1}H NMR (DMSO-d_6, 500 MHz, ppm), δ_H 8.57 (s, 1H), 7.79 (s, 1H), 7.20-7.18 (m, 4H), 5.37 (s, 2H), 4.97 (d, $^2J_{H-H}$ = 20.5 Hz, 1H), 4.79-4.68 (m, 2H), 4.43-4.41 (m, 2H), 4.21-4.19 (m, 7H), 3.66 (d, $^2J_{H-H}$ = 20.5 Hz, 1H), 3.57 (d, $^2J_{H-H}$ = 19.7 Hz, 1H), 3.48 (d, $^2J_{H-H}$ = 19.7 Hz, 1H); ^{13}C{^{1}H} NMR (DMSO-d_6, 125 MHz, ppm), δ_C 149.6, 149.0, 148.9, 148.8, 148.4, 148.1, 147.9x2, 147.6, 147.1, 146.1, 144.2, 126.8, 124.1, 123.8x2, 122.1, 120.9,

82.5, 79.2, 79.0, 78.7, 68.7x2, 68.6 (5C), 68.3 (2C), 49.1, 42.7, 41.3; HRMS (ESI) *m/z* $[M]^+$ calcd. for $C_{34}H_{23}FeN_3$ 529.1241, found 529.1242; UV-Vis, λ_{max} ($CHCl_3$, $2\cdot10^{-5}$ M) 290, 334 nm; Emission spectrum ($CHCl_3$, $2\cdot10^{-5}$ M, λ_{exc} = 285 nm) 390 nm; R_f (2% $CH_3OH/CHCl_3$) = 0.40.

1.9. Preparation of the cesium cations electrochemical sensor

The glassy carbon surface was first polished at the microcloth polishing pad with a slurry of alumina (1.0 μm diameter, Buehler). Then, the GC surface was rinsed with distilled water to remove the Al_2O_3 residues. In the next step to produce more carboxyl groups at GC surface the electrode was oxidized in 0.1 M H_2SO_4 by cycling in the potential range -0.35 – 1.3 – -0.35 V with scan rate 100 $mV\cdot s^{-1}$. On the such prepared electrode surface the 10-μL droplet of 0.02 mM monoferrocenylsumanene **7** or monoferrocenylsumanene **8** solution (CH_2Cl_2 : DMSO (1:1 v/v)) with addition of 150 mM $TBAPF_6$ and 5% nafion® was placed, and left to dry in desiccator.

2. NMR spectra

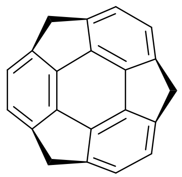

1

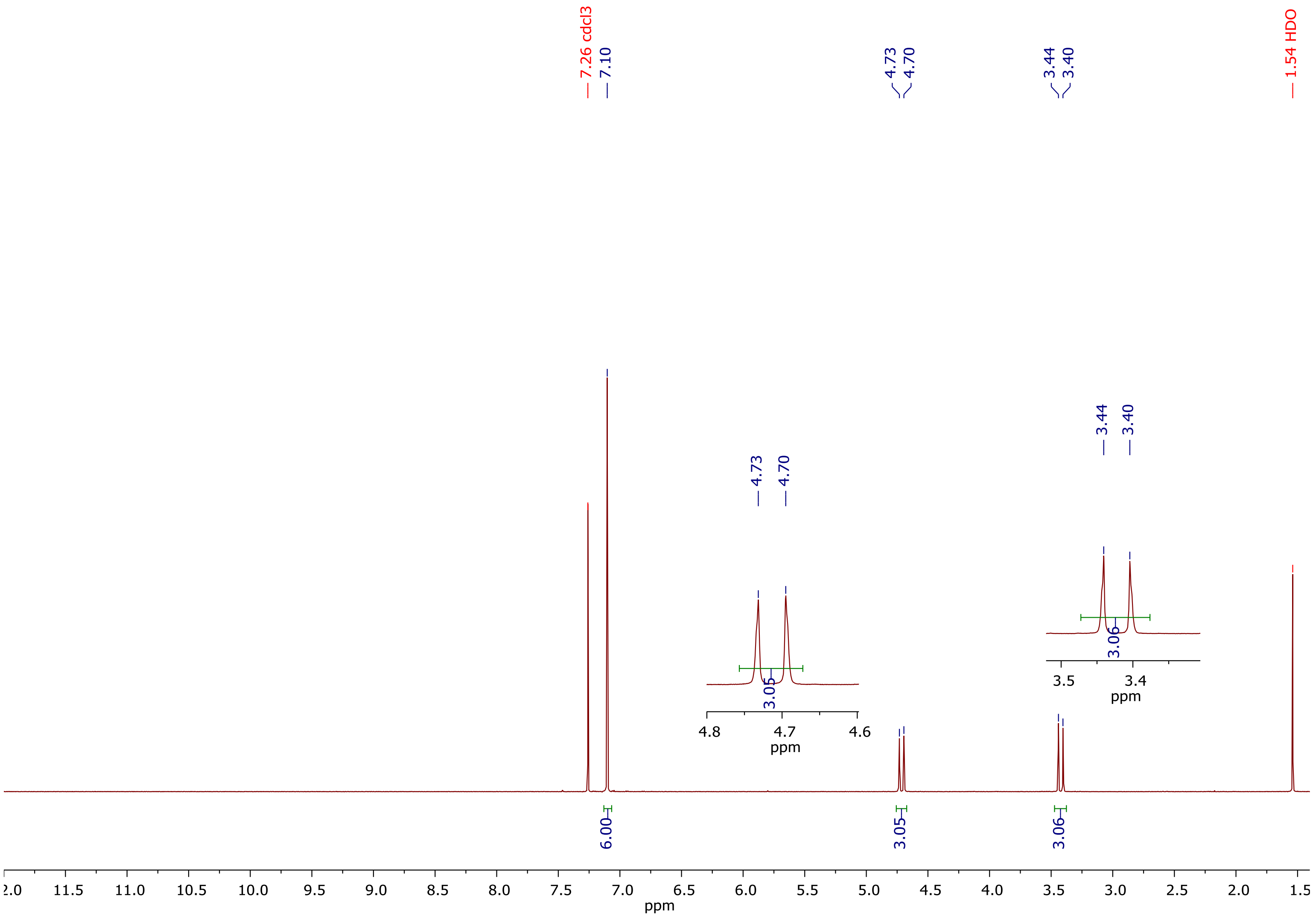

Figure S1. ^{1}H NMR (500 MHz, $CDCl_3$) spectrum of sumanene (**1**).

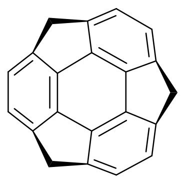

1

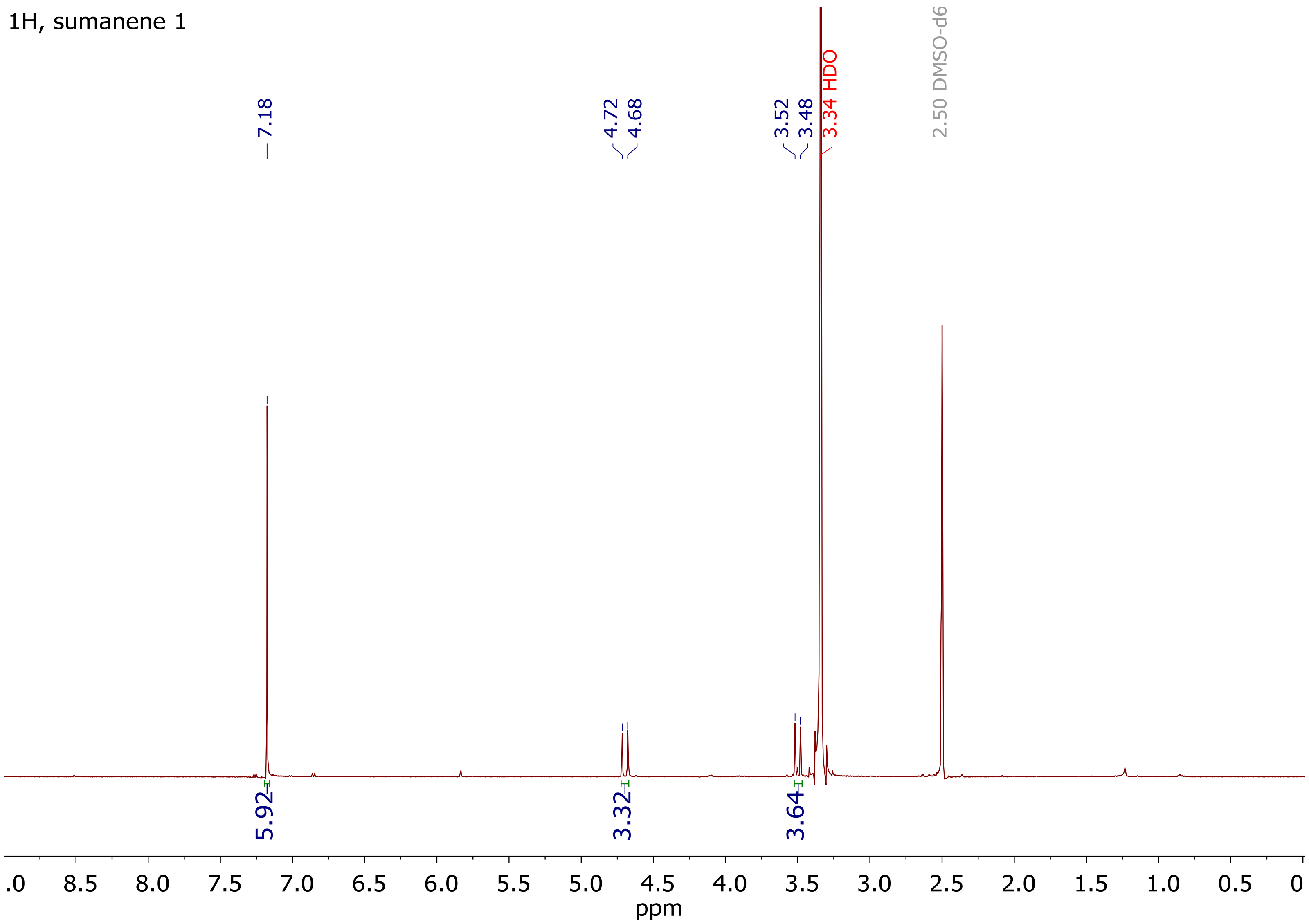

Figure S2. ^{1}H NMR (500 MHz, DMSO-d_6) spectrum of sumanene (**1**).

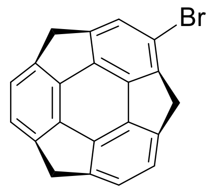

2a

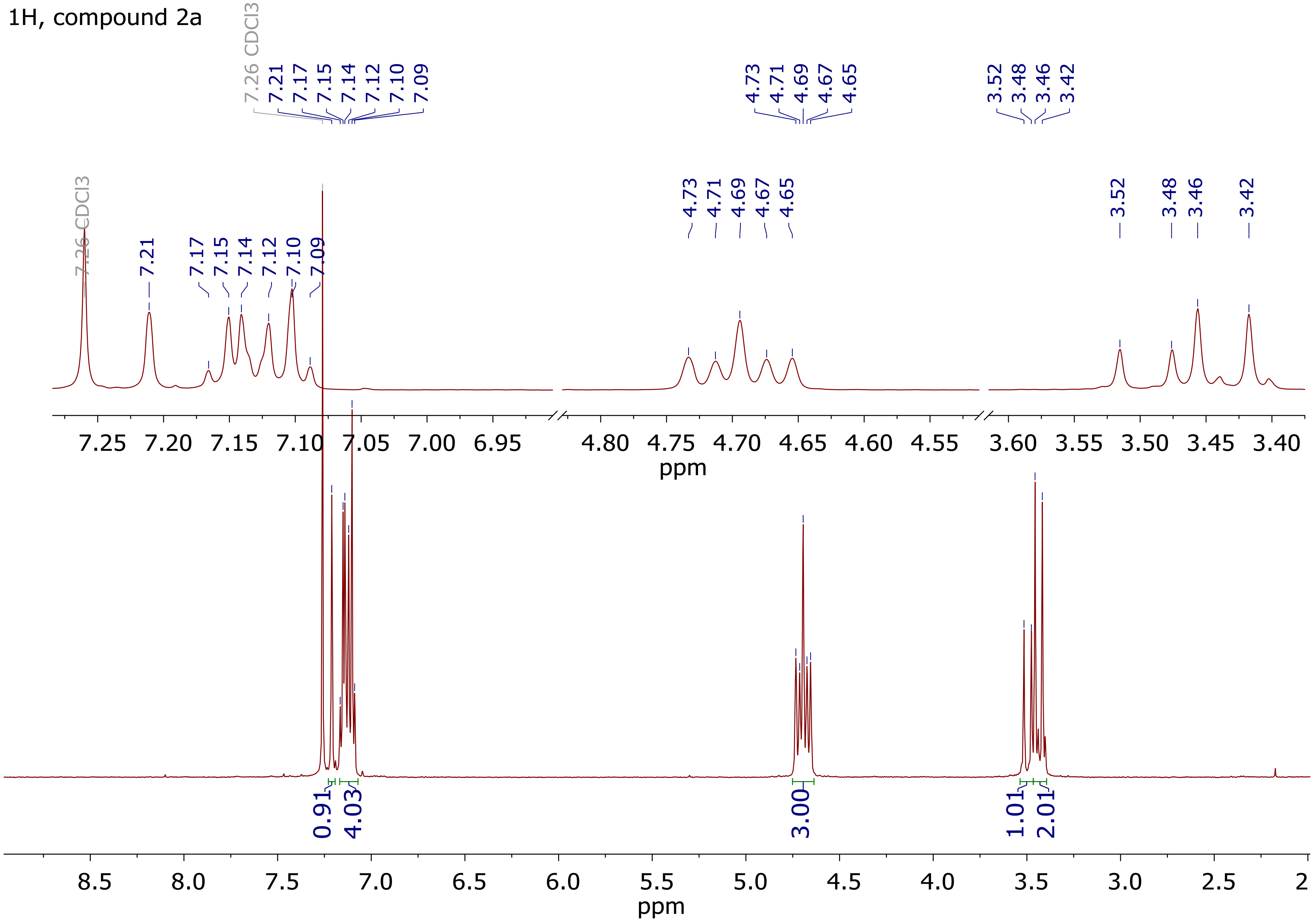

Figure S3. ^{1}H NMR (500 MHz, $CDCl_3$) spectrum of 2-bromosumanene (**2a**).

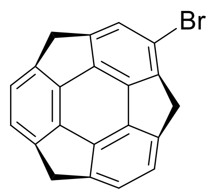

2a

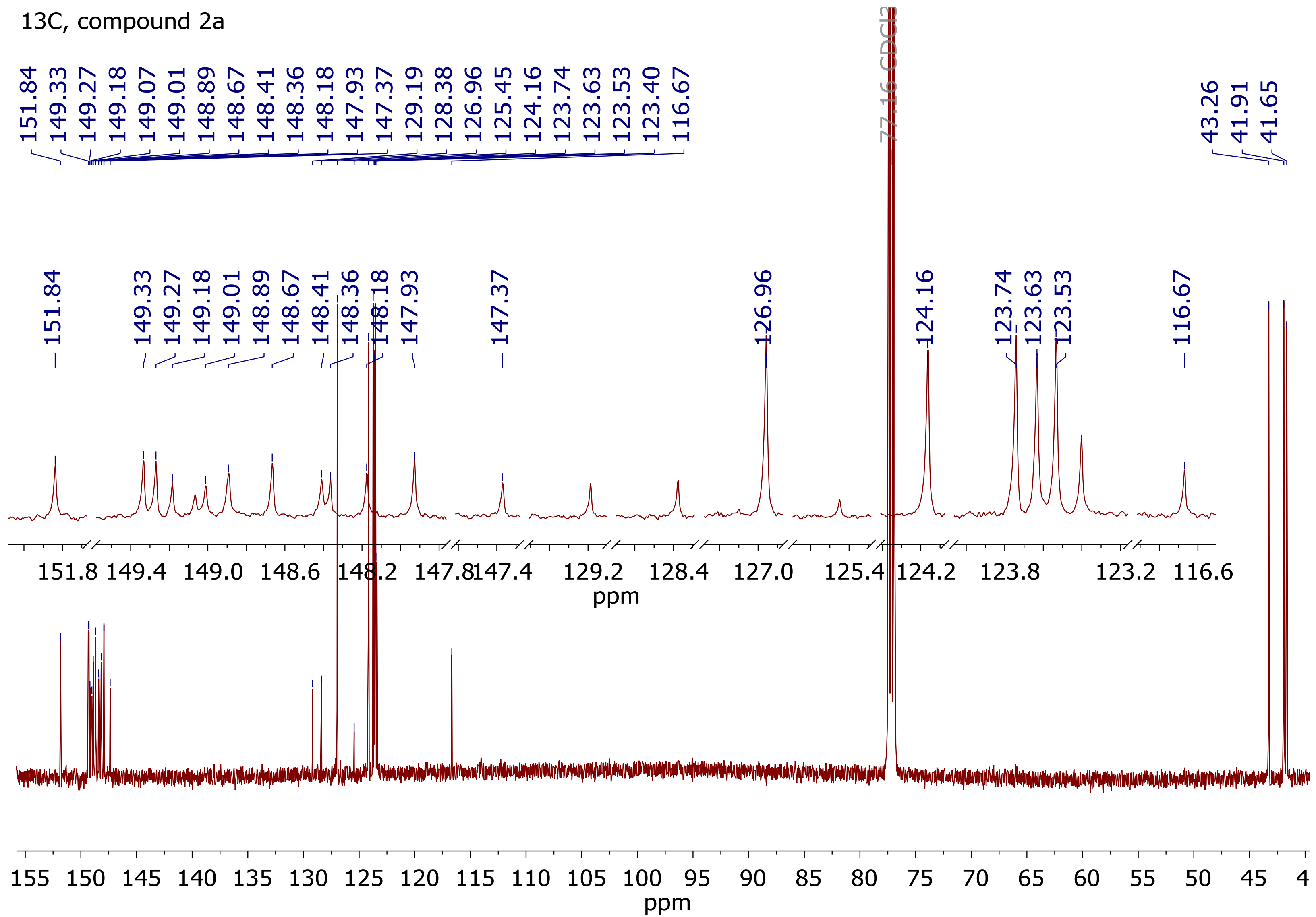

Figure S4. $^{13}C\{^{1}H\}$ NMR (125 MHz, $CDCl_3$) spectrum of 2-bromosumanene (**2a**). The unlabeled peaks come from the traces of dibromosumanenes.

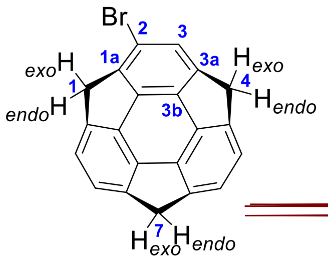

2a

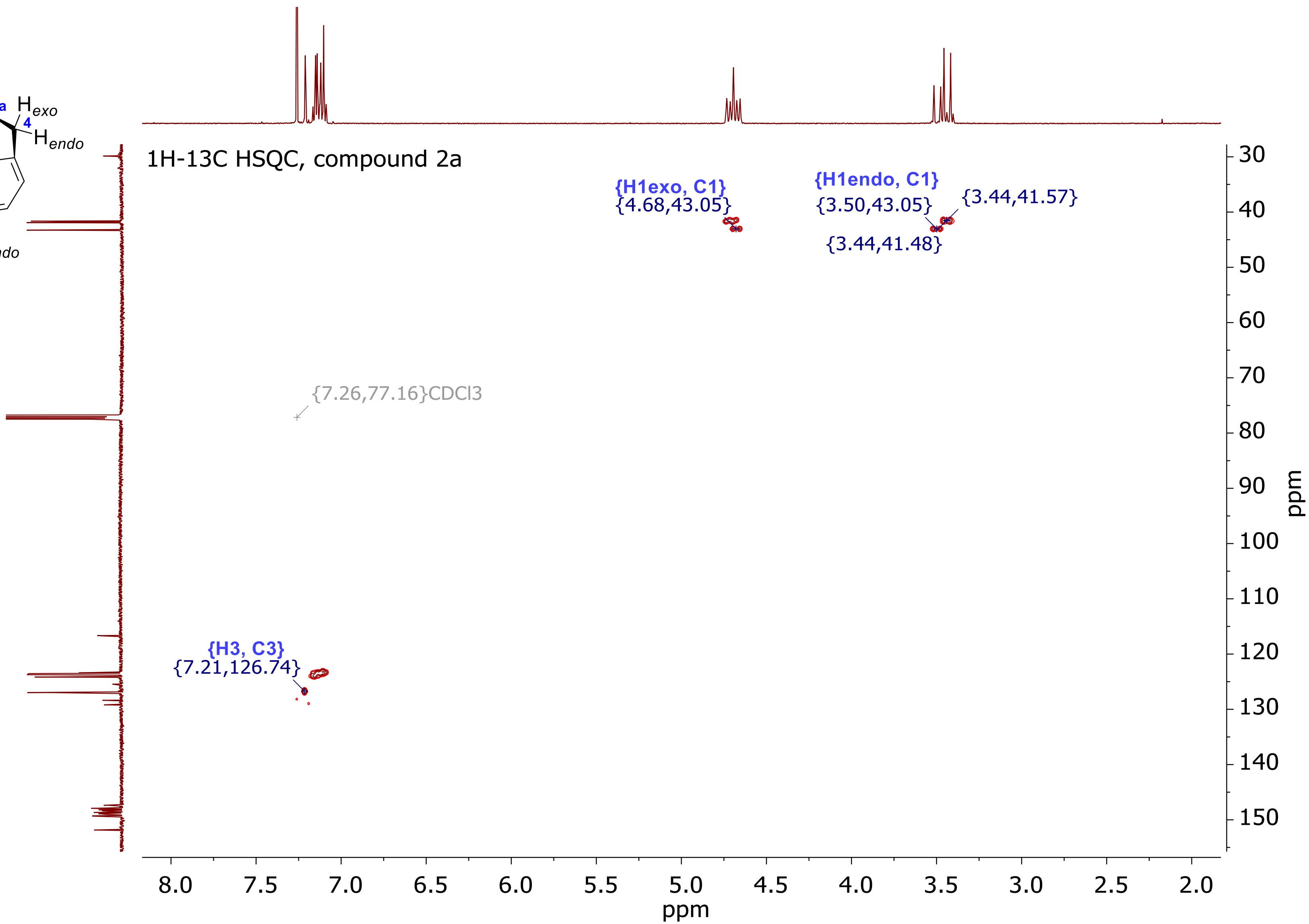

Figure S5. ^{1}H-^{13}C HSQC NMR ($CDCl_3$) spectrum of 2-bromosumanene (**2a**).

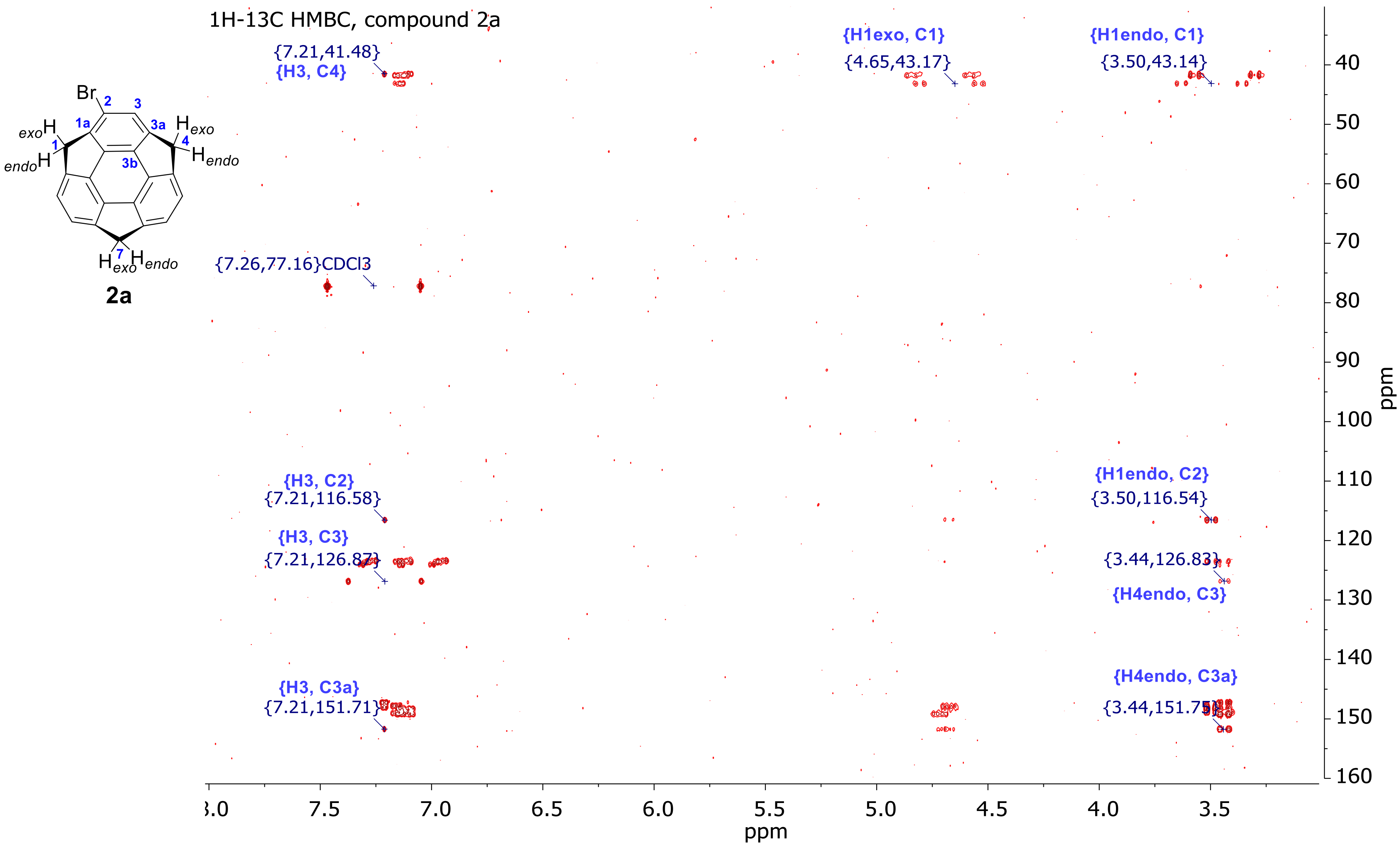

Figure S6. ^{1}H-^{13}C HMBC NMR ($CDCl_3$) spectrum of 2-bromosumanene (**2a**).

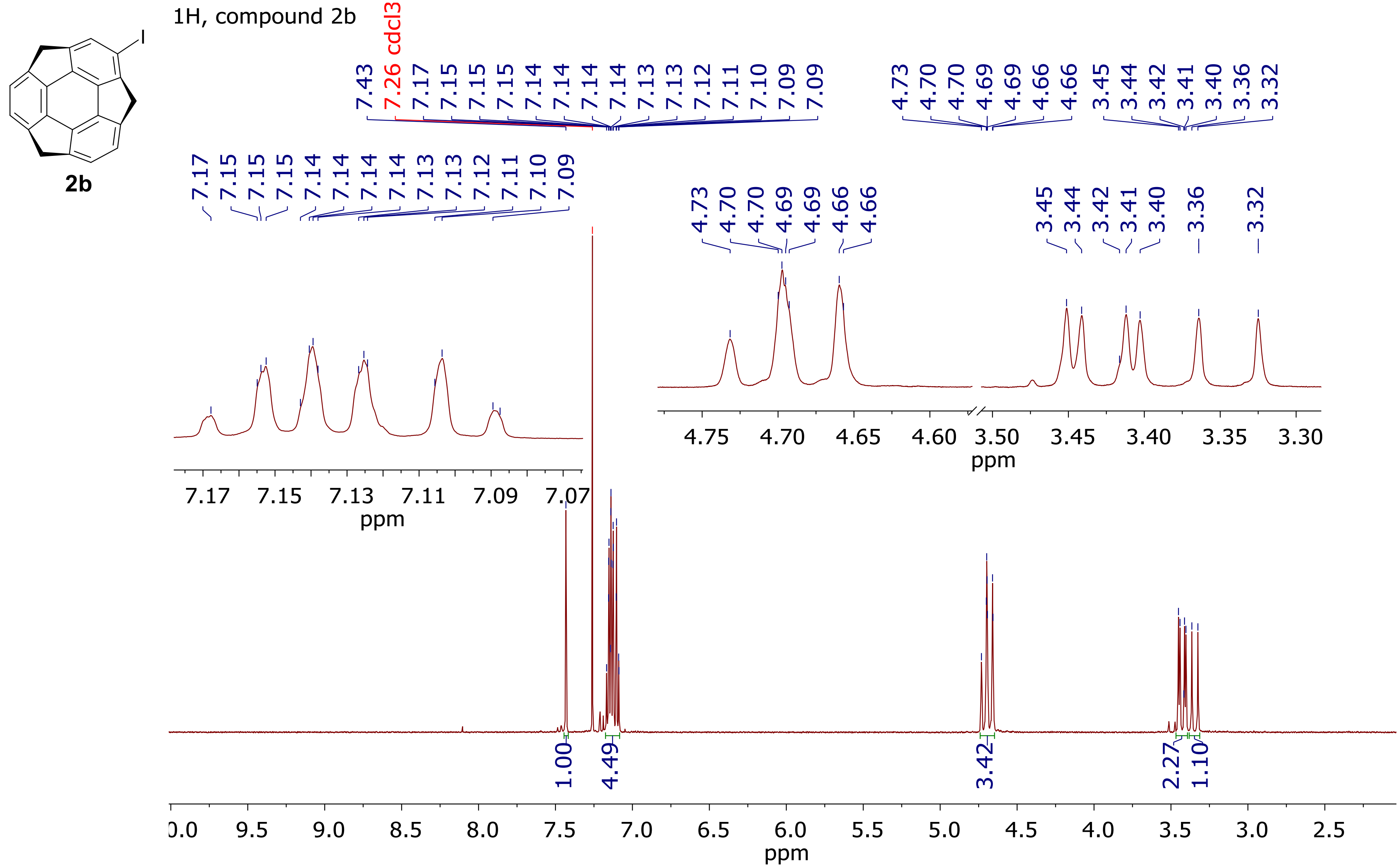

Figure S7. ^{1}H NMR (500 MHz, $CDCl_3$) spectrum of 2-iodosumanene (**2b**).

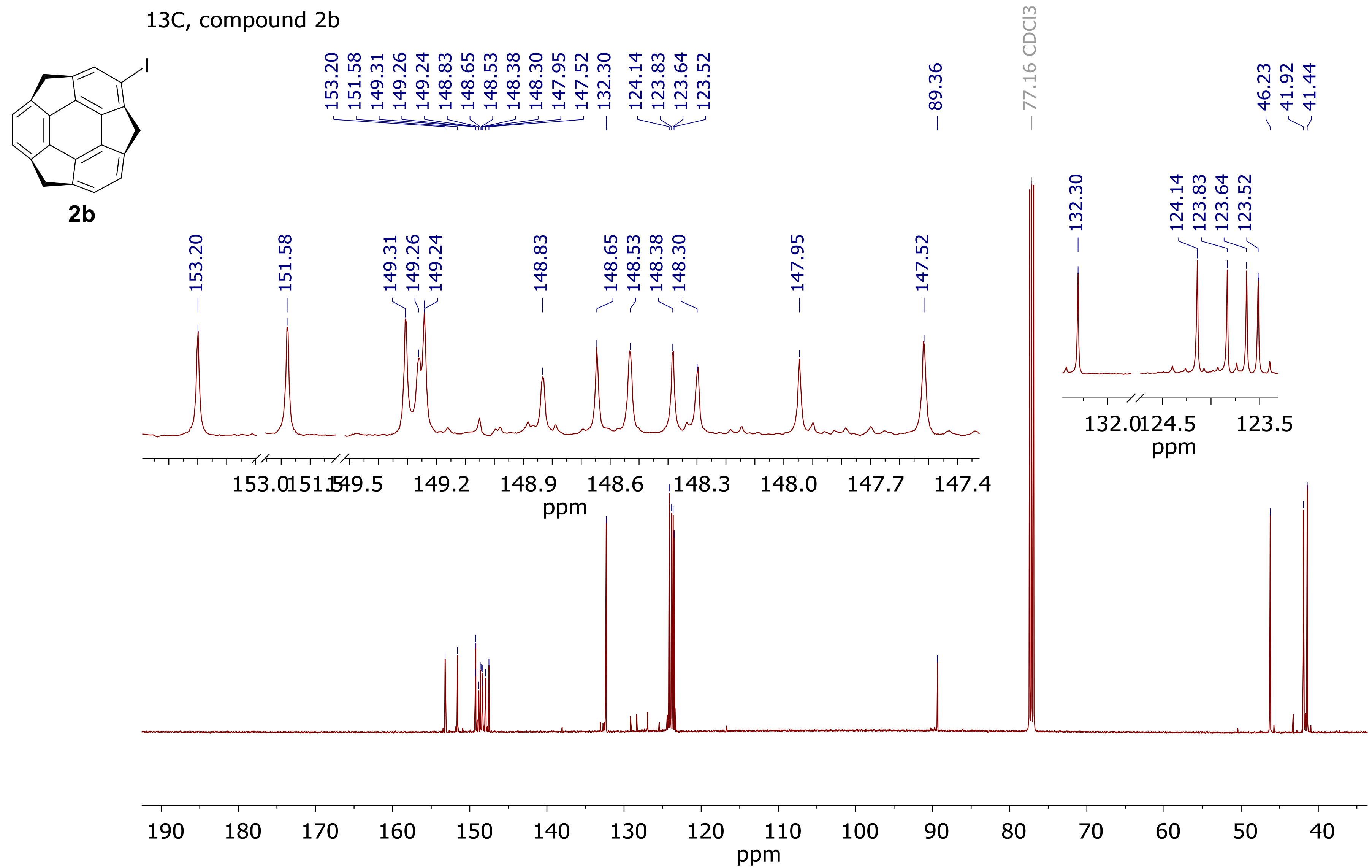

Figure S8. $^{13}C\{^{1}H\}$ NMR (125 MHz, $CDCl_3$) spectrum of 2-iodosumanene (**2b**).

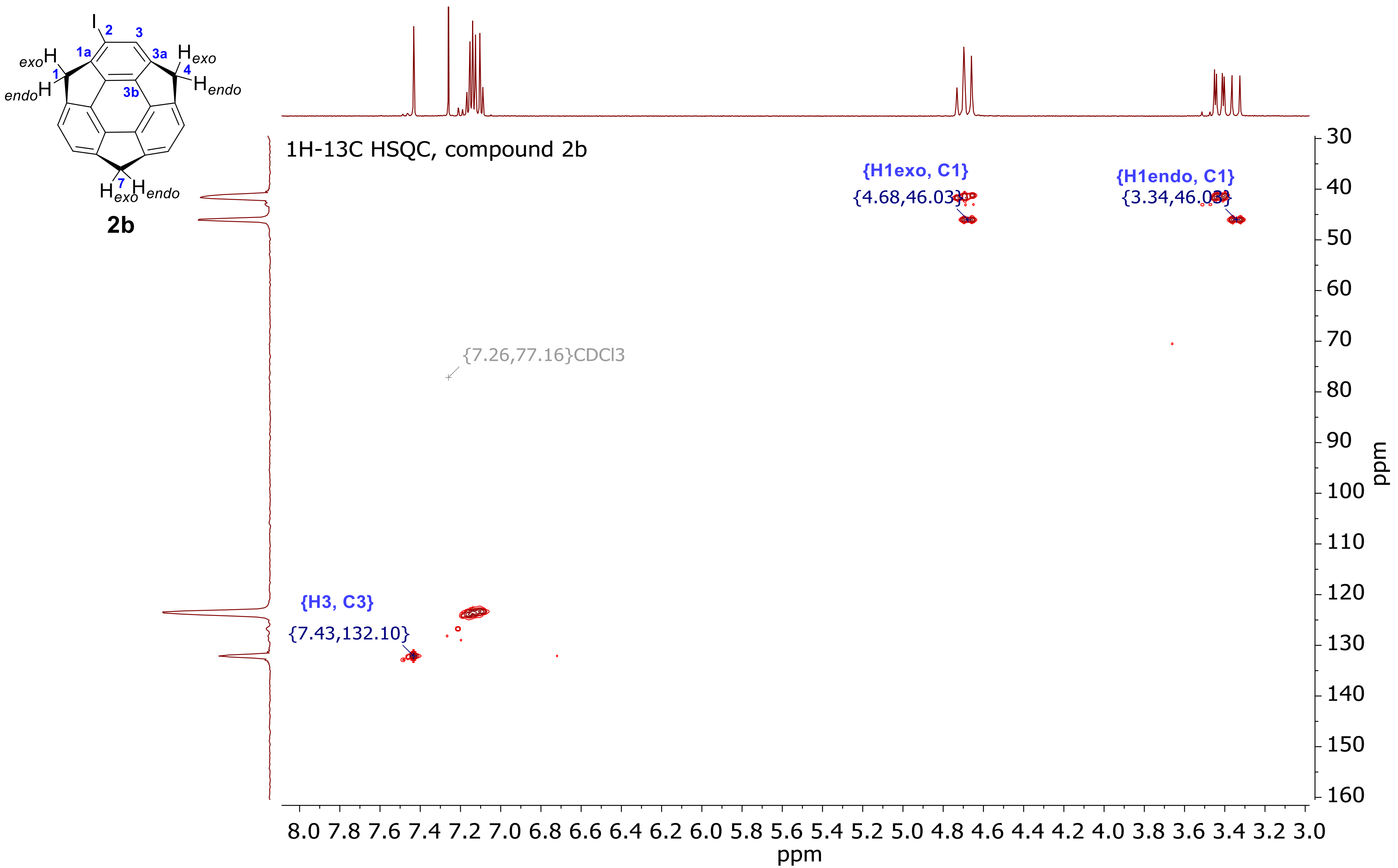

Figure S9. ^{1}H-^{13}C HSQC NMR ($CDCl_3$) spectrum of 2-iodosumanene (**2b**).

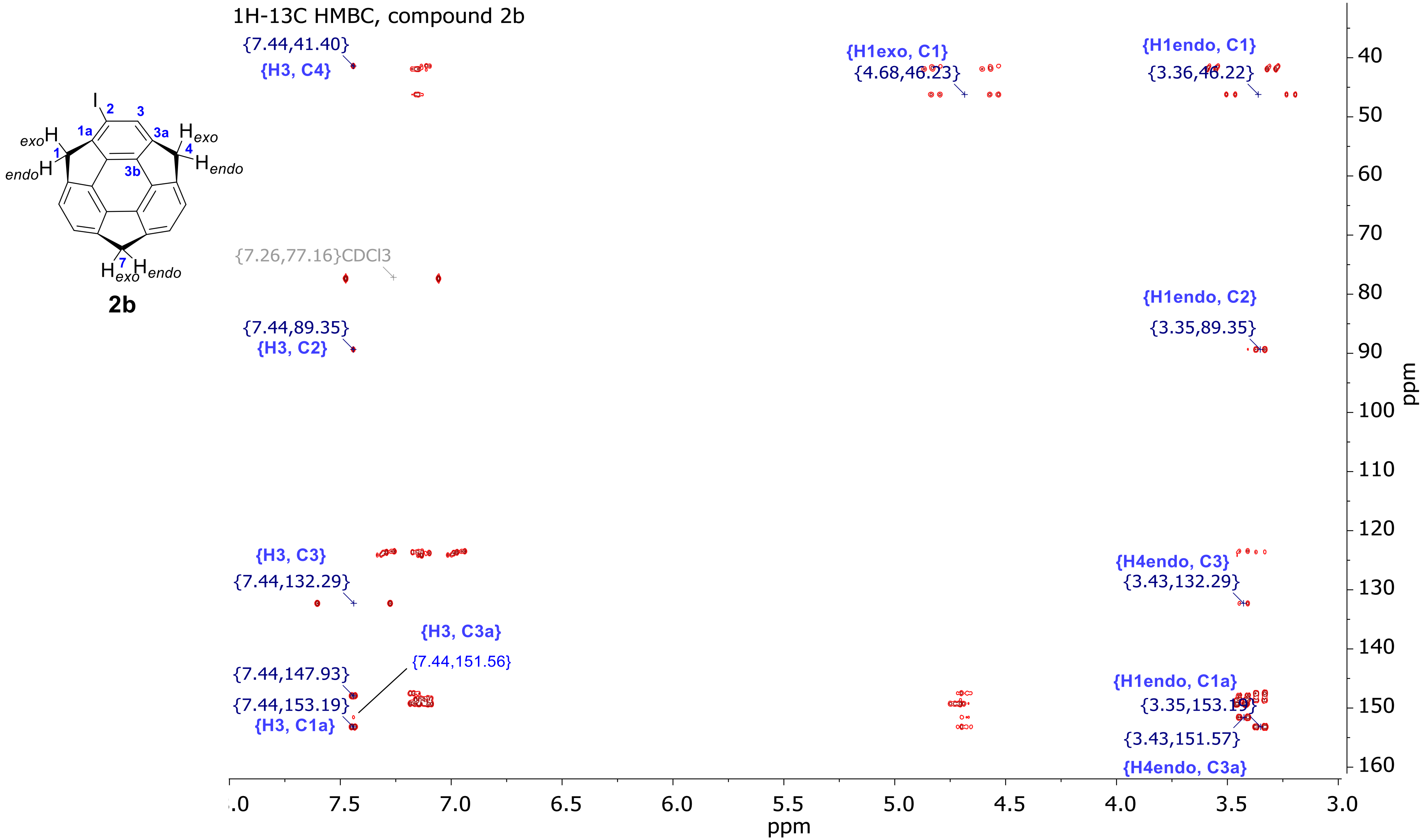

Figure S10. ^{1}H-^{13}C HMBC NMR ($CDCl_3$) spectrum of 2-iodosumanene (**2b**).

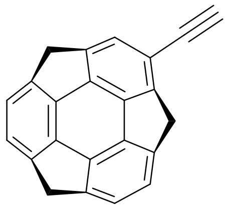

6

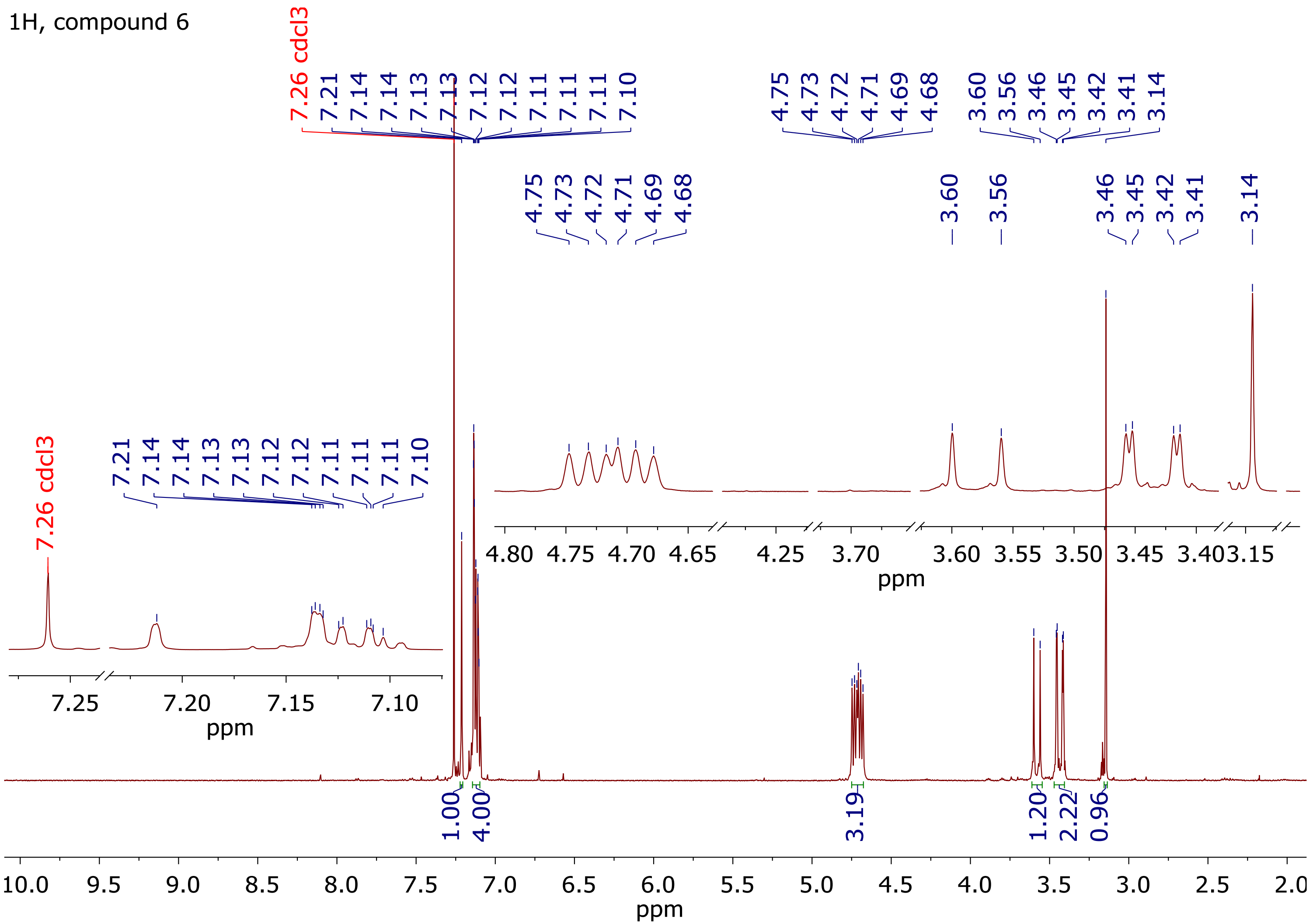

Figure S11. ^{1}H NMR (500 MHz, $CDCl_3$) spectrum of 2-ethynylsumanene (**6**). The distinctive singlet at 3.14 ppm (^{1}H NMR, $CDCl_3$; the C(*sp*)-H) and two signals at 83.4 ppm and 78.3 ppm ({^{1}H}^{13}C NMR, $CDCl_3$; the C(*sp*)) confirmed the presence of the acetylene group. The ^{1}H NMR spectrum also comprised the signals characteristic for the sumanene benzylic H_{endo} protons (3.60-3.41 ppm), benzylic H_{exo} protons (4.75-4.68 ppm), and aromatic protons (7.21-7.11 ppm).

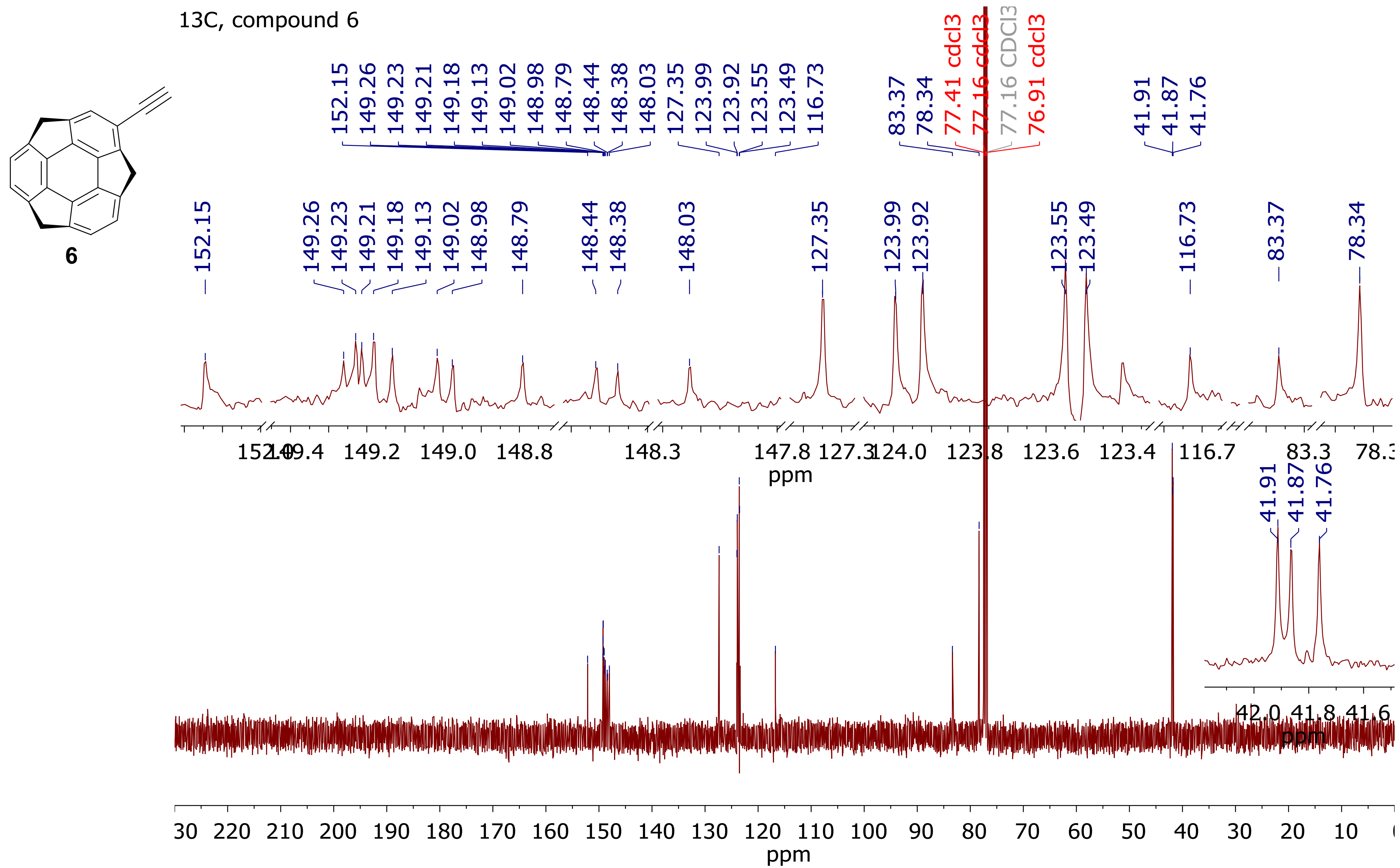

Figure S12. $^{13}C\{^{1}H\}$ NMR (125 MHz, $CDCl_3$) spectrum of 2-ethynylsumanene (**6**).

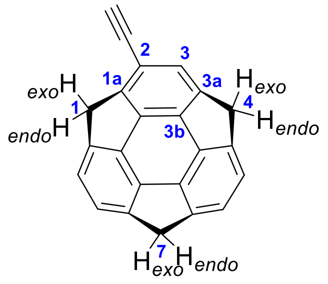

6

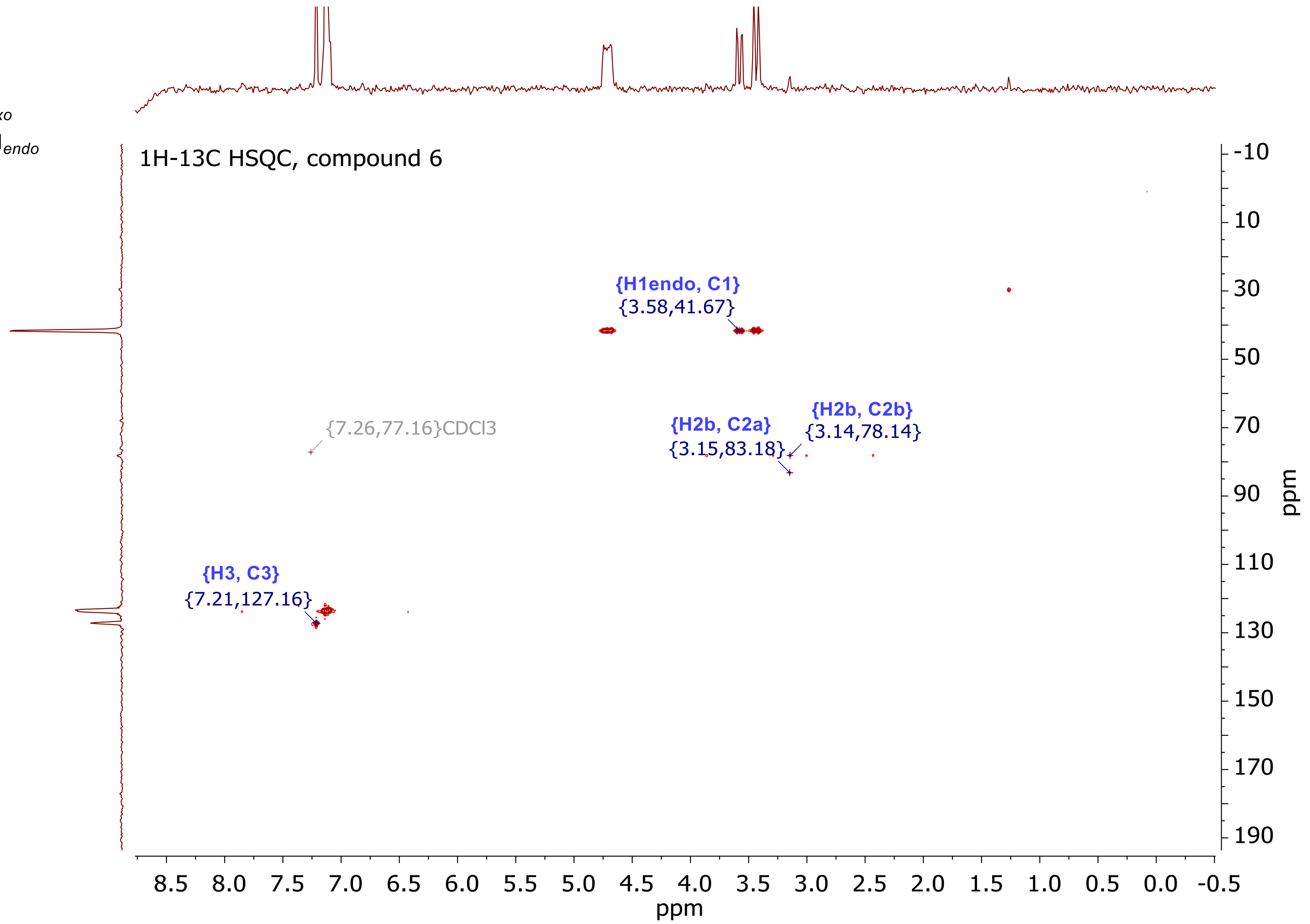

Figure S13. ^{1}H-^{13}C HSQC NMR ($CDCl_3$) spectrum of 2-ethynylsumanene (**6**).

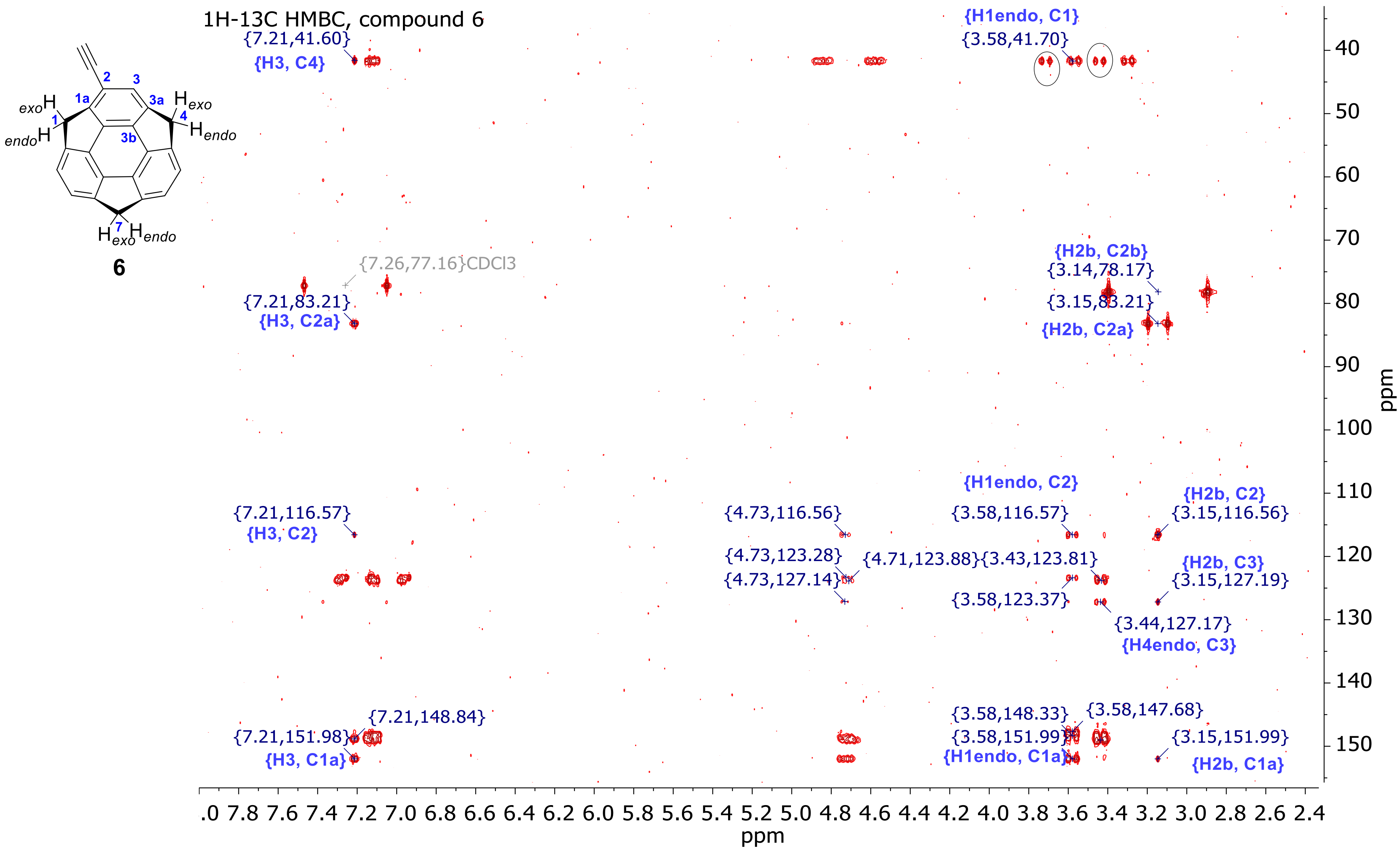

Figure S14. ^{1}H-^{13}C HMBC NMR ($CDCl_3$) spectrum of 2-ethynylsumanene (**6**).

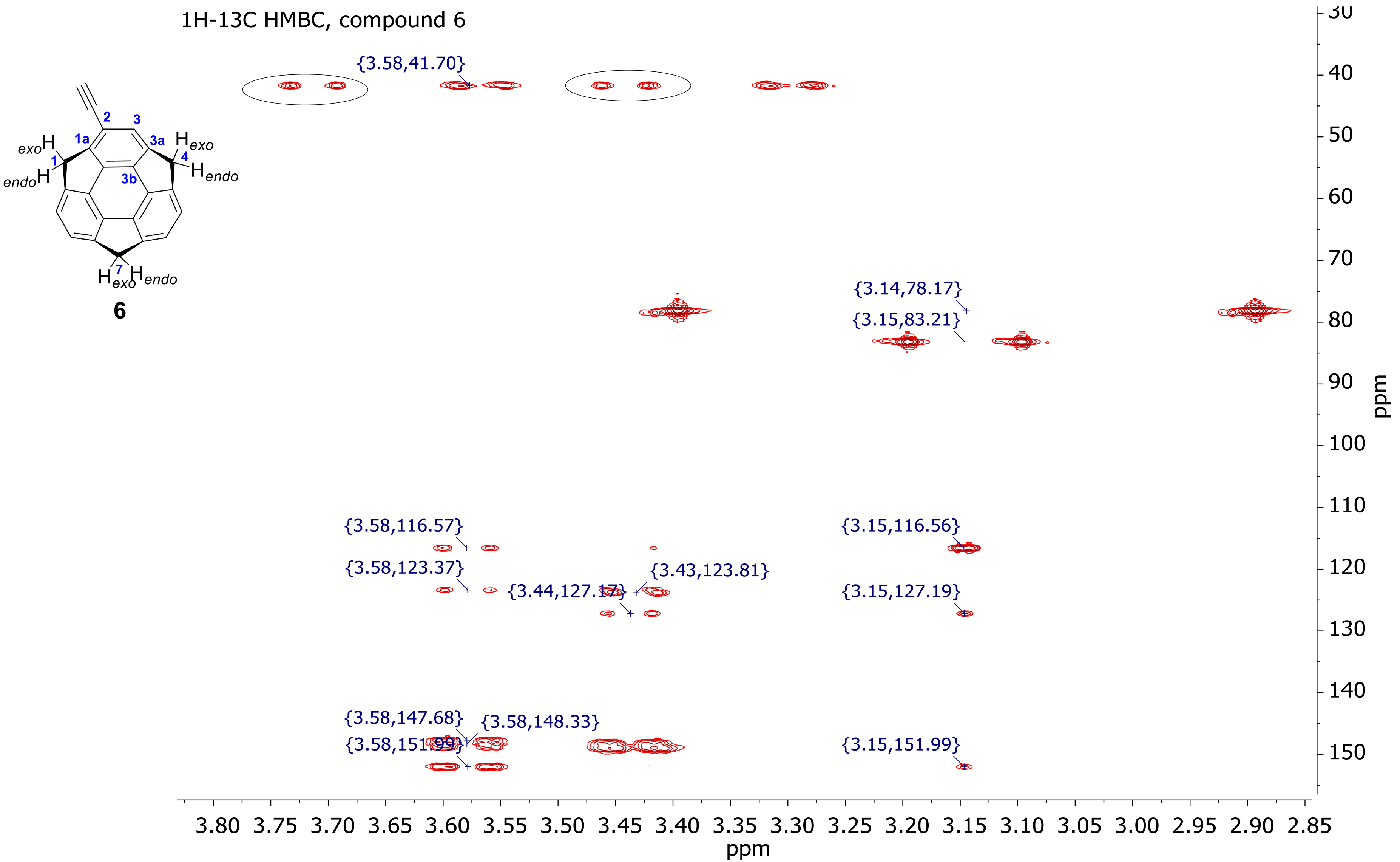

Figure S15. ^{1}H-^{13}C HMBC NMR ($CDCl_3$) spectrum of 2-ethynylsumanene (**6**) - inset.

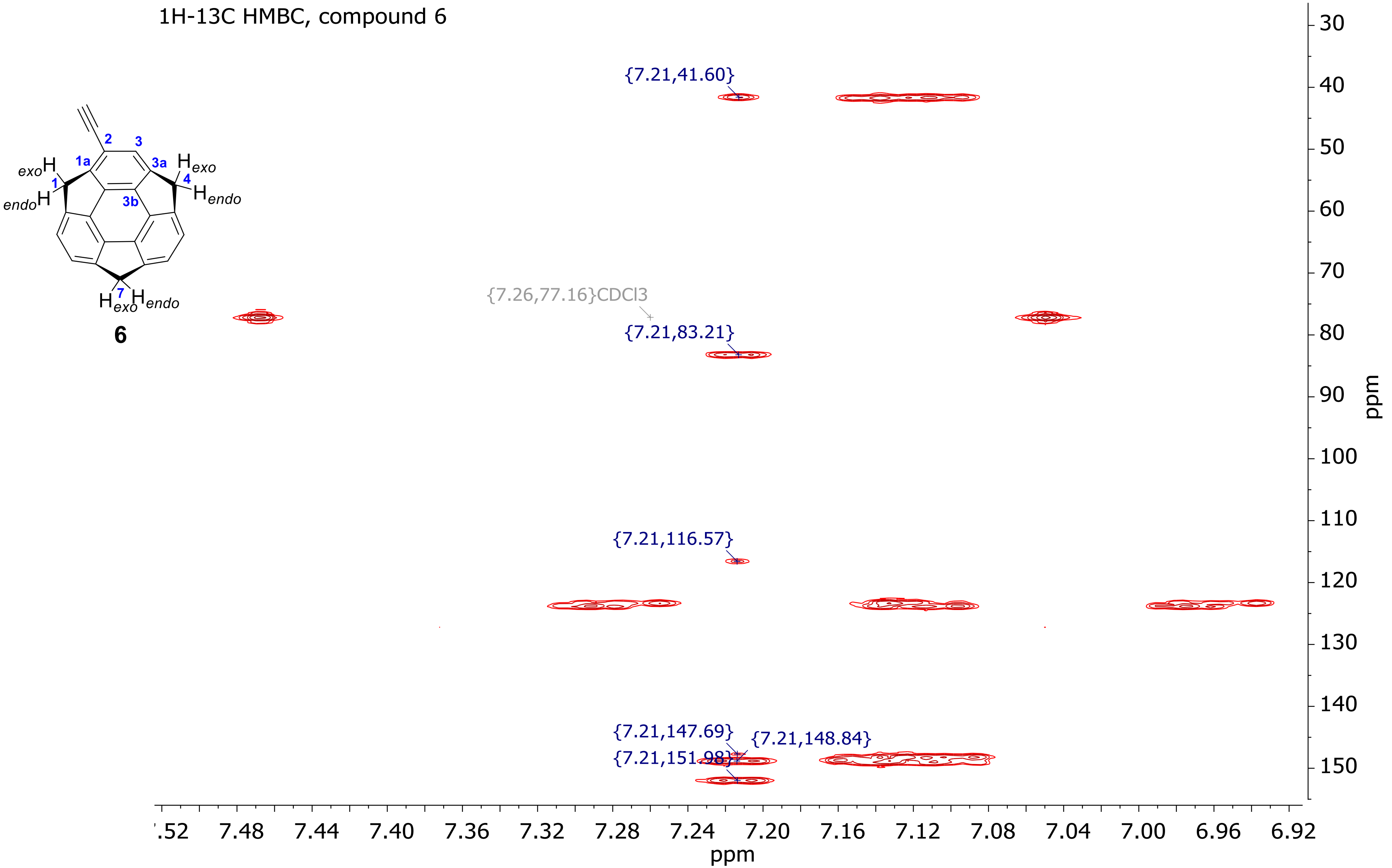

Figure S16. ^{1}H-^{13}C HMBC NMR ($CDCl_3$) spectrum of 2-ethynylsumanene (**6**) - inset.

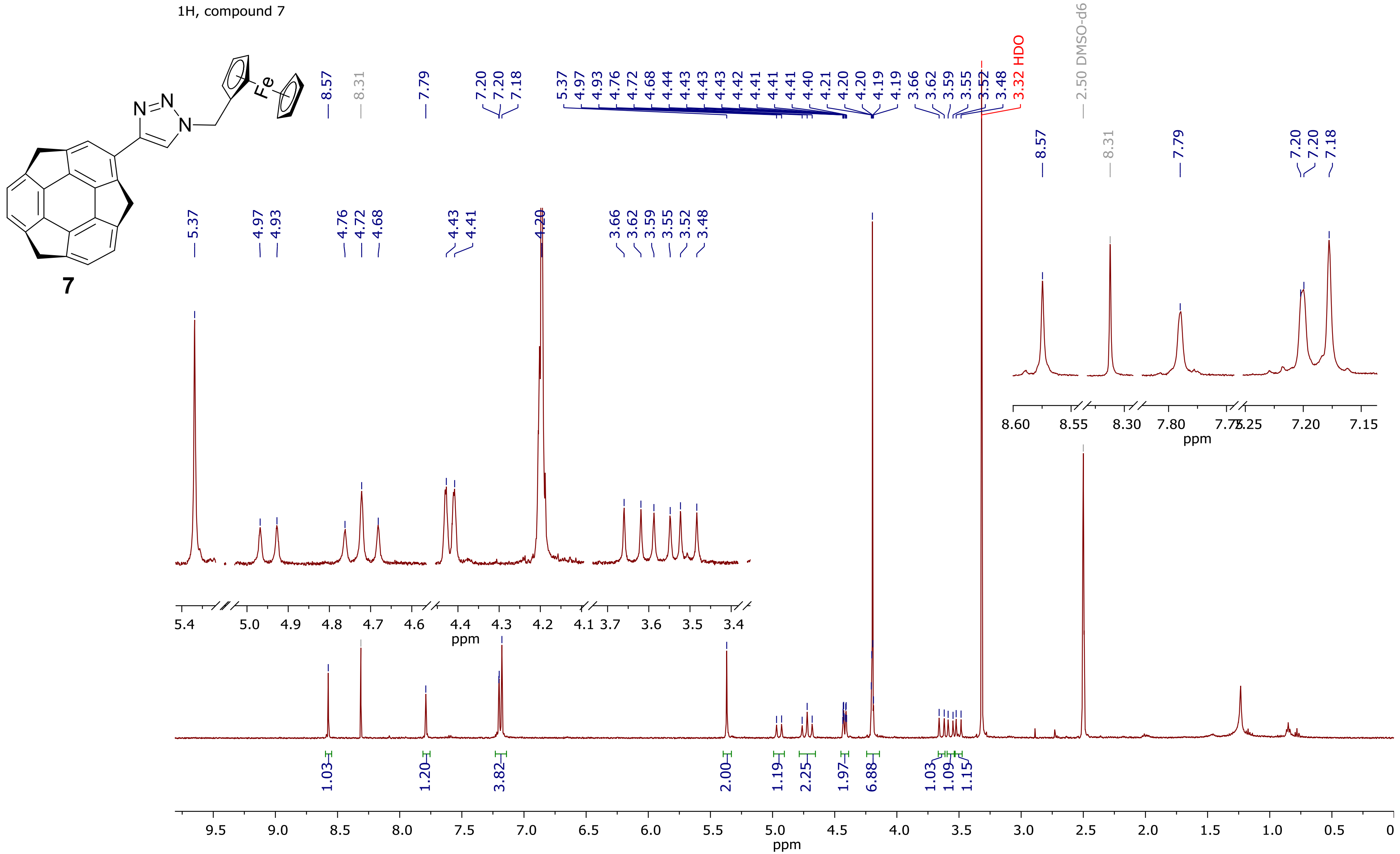

Figure S17. ^{1}H NMR (500 MHz, DMSO-d_6) spectrum of monoferrocenylsumanene **7**.

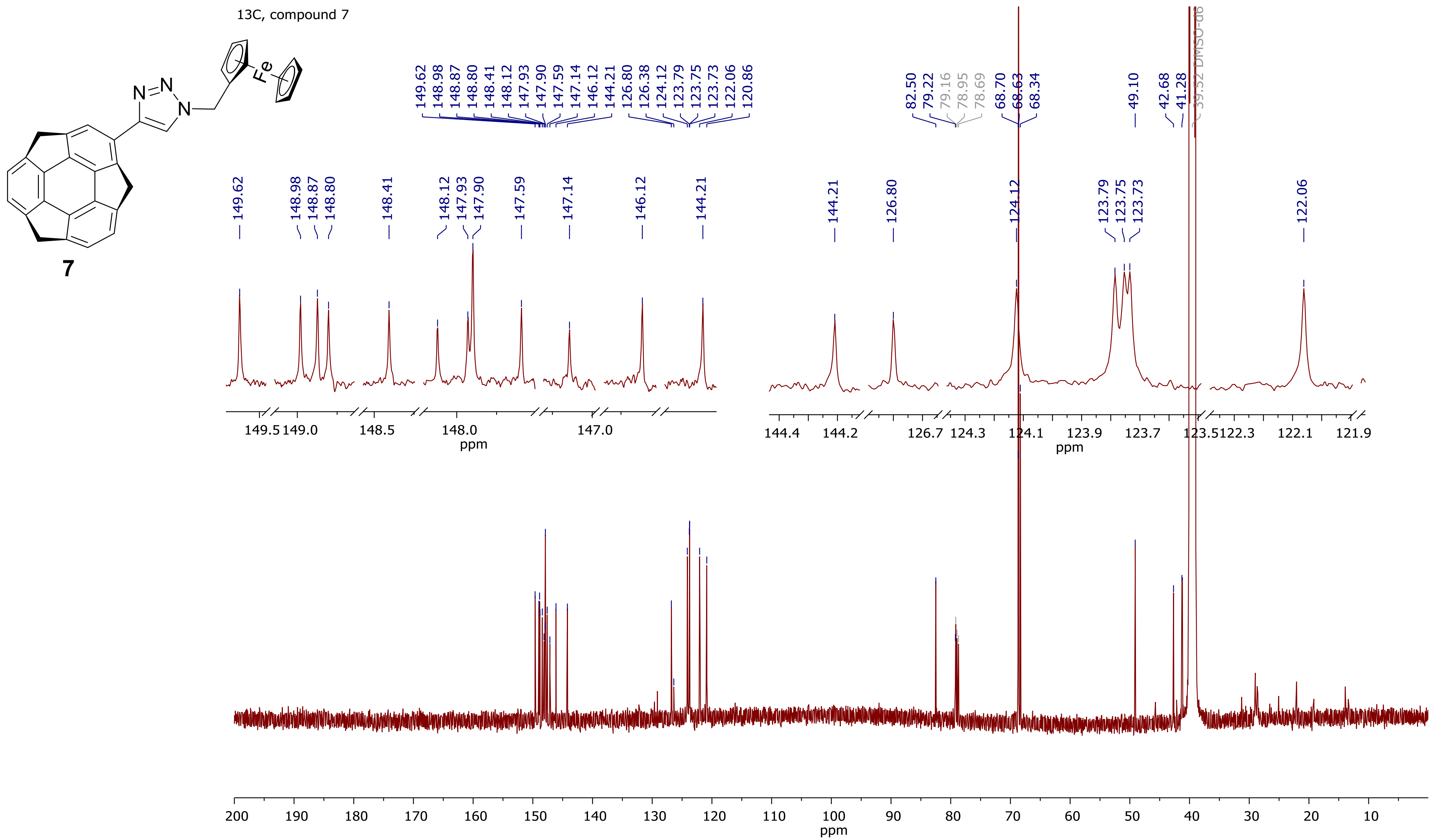

Figure S18. $^{13}C\{^1H\}$ NMR (125 MHz, DMSO-d_6) spectrum of monoferrocenylsumanene **7**.

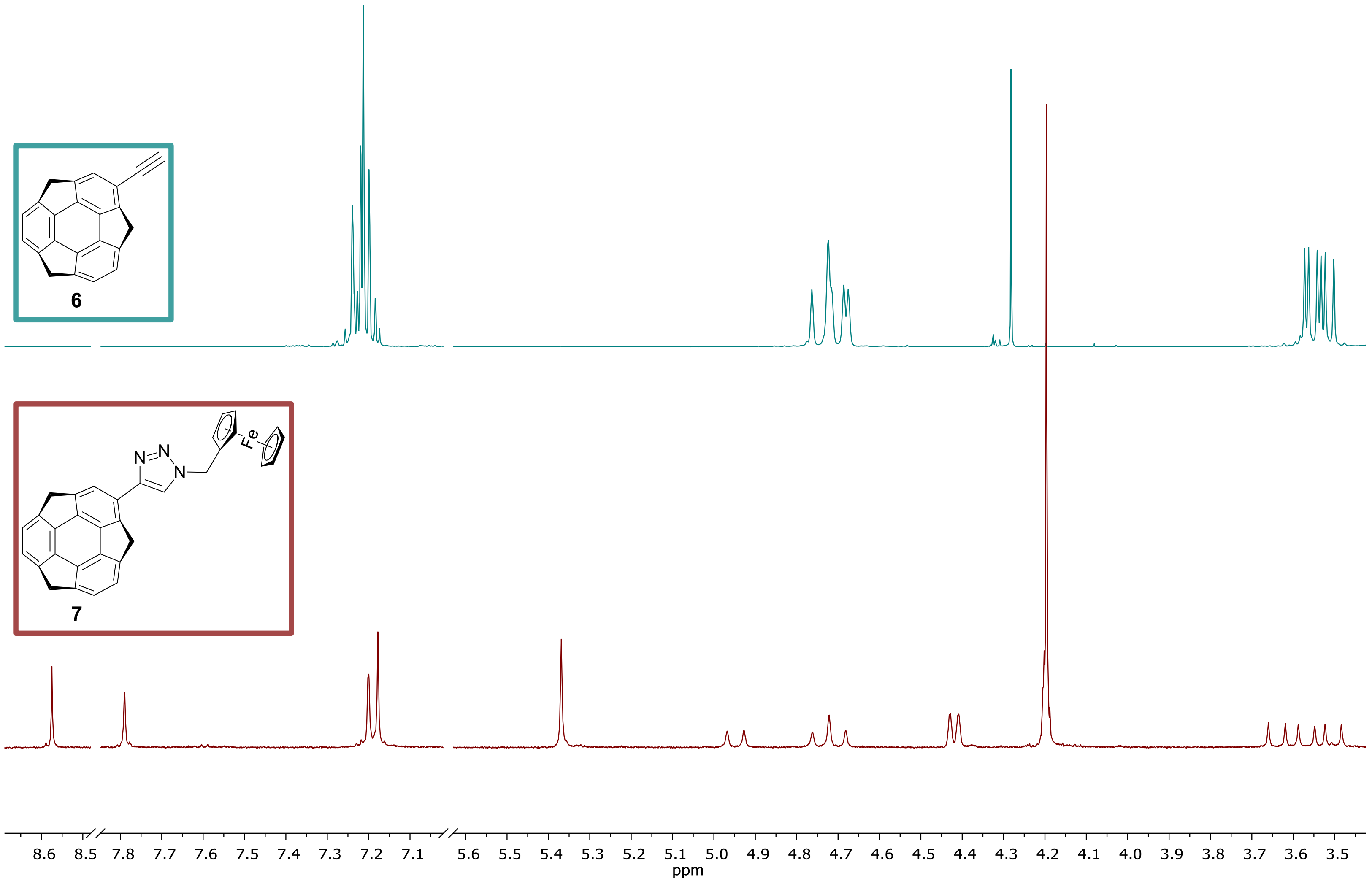

Figure S19. Comparison of ^{1}H NMR spectra (500 MHz, DMSO-d_6) 2-ethynylsumanene (**6**), and monoferrocenylsumanene **7**. Selected insets of the spectra are presented.

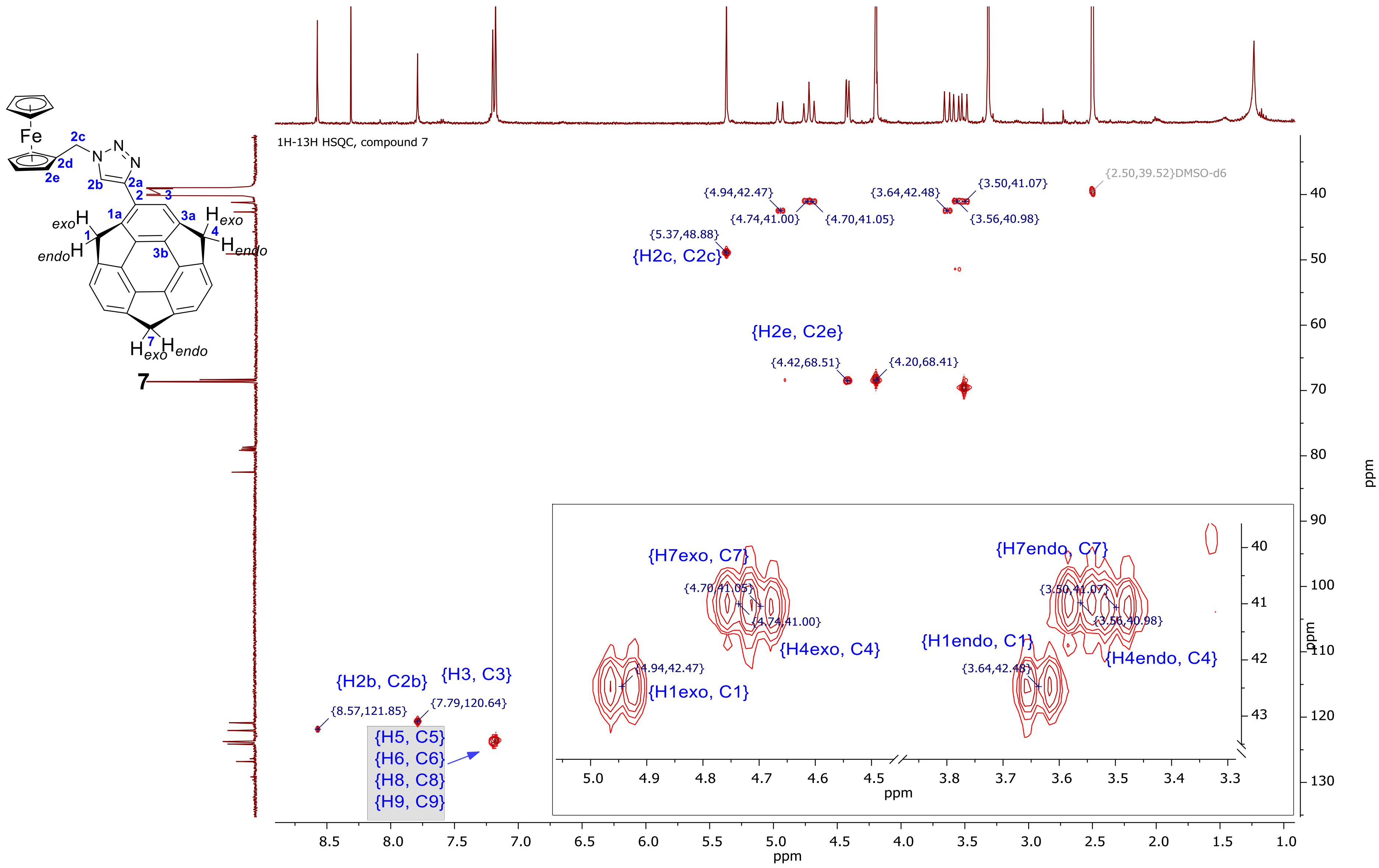

Figure S20. ^{1}H-^{13}C HSQC NMR (DMSO-d_6) spectrum of monoferrocenylsumanene **7**.

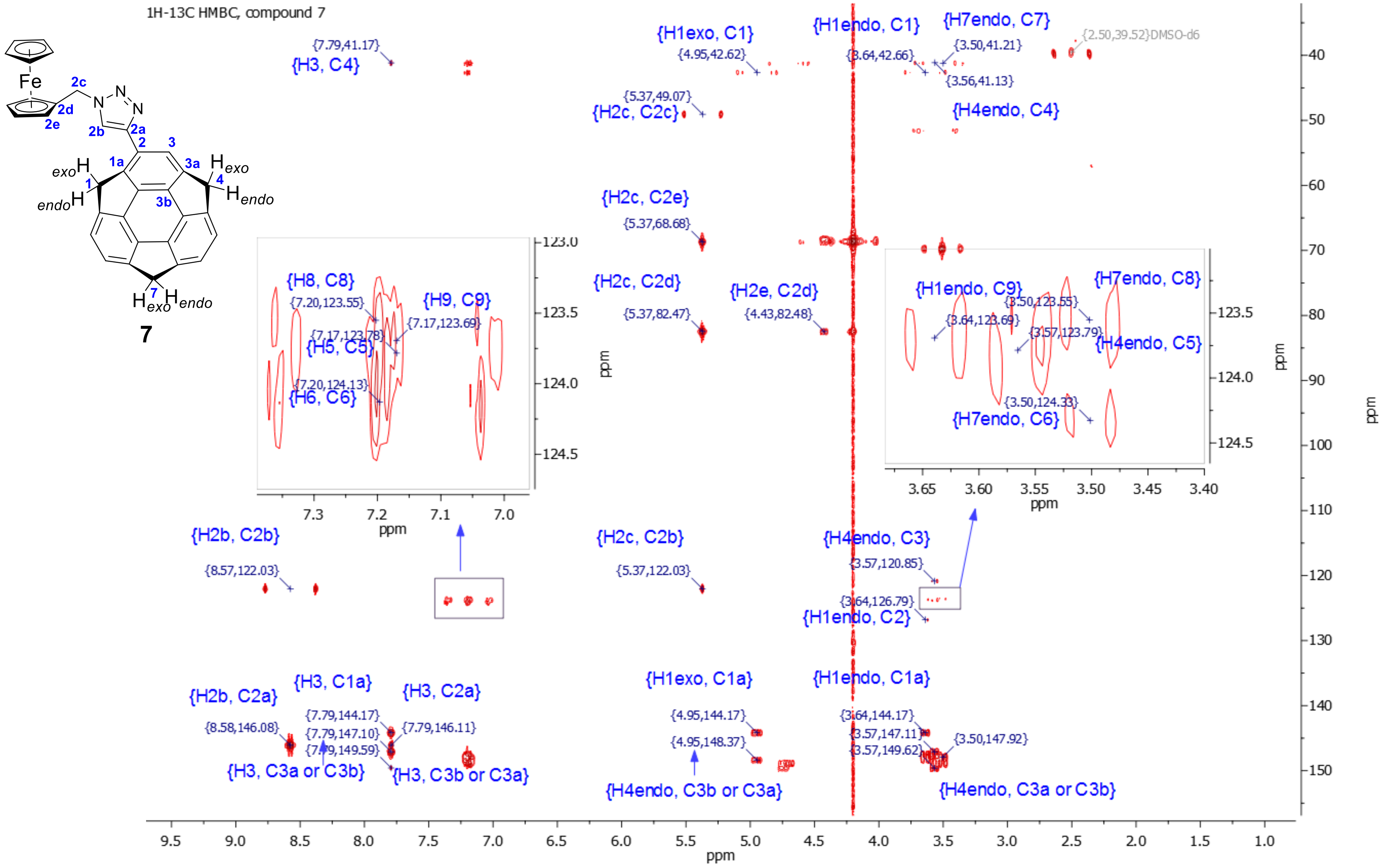

Figure S21. ^{1}H-^{13}C HMBC NMR (DMSO-d_6) spectrum of monoferrocenylsumanene **7**.

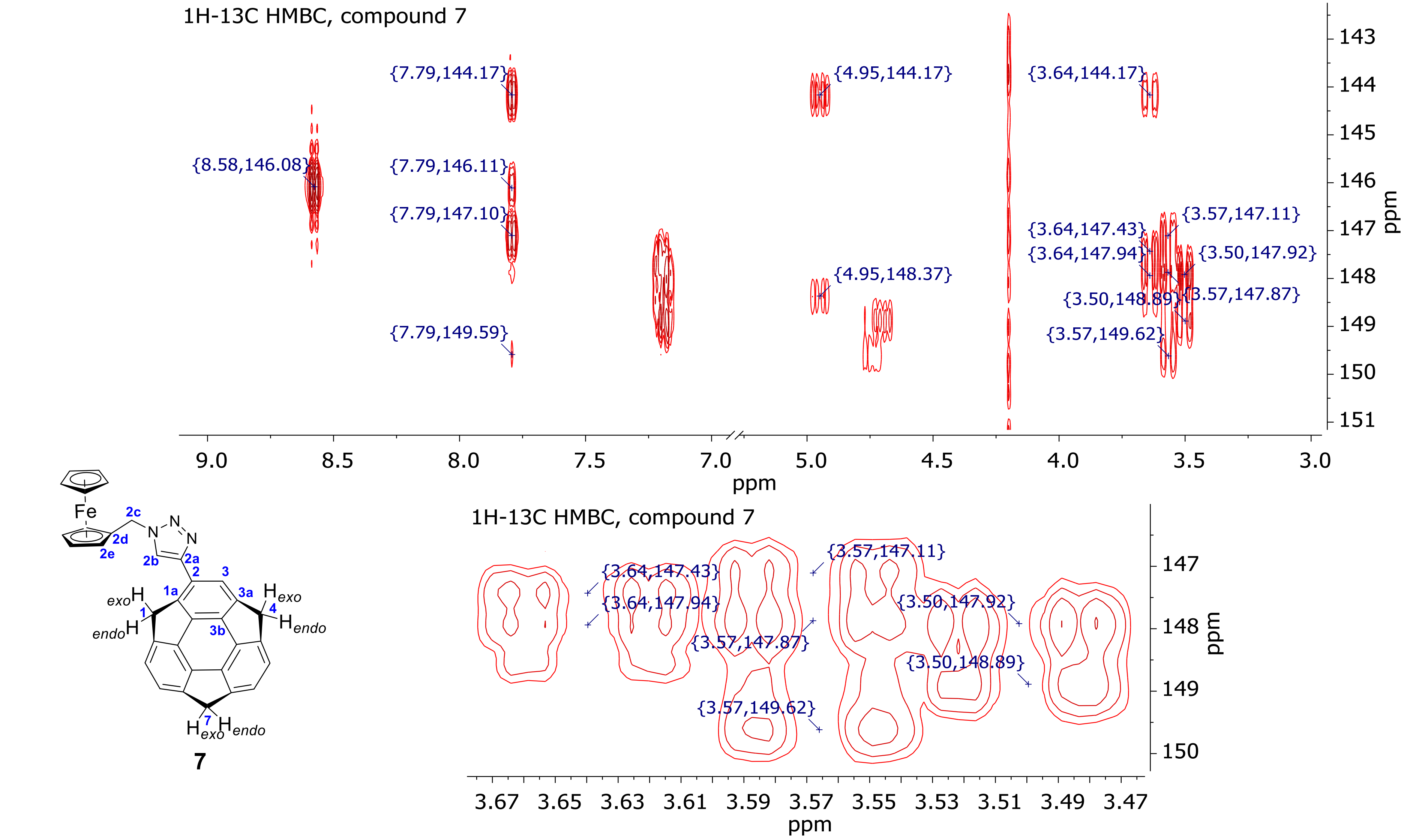

Figure S22. ^{1}H-^{13}C HMBC NMR (DMSO-d_6) spectrum of monoferrocenylsumanene **7** – insets.

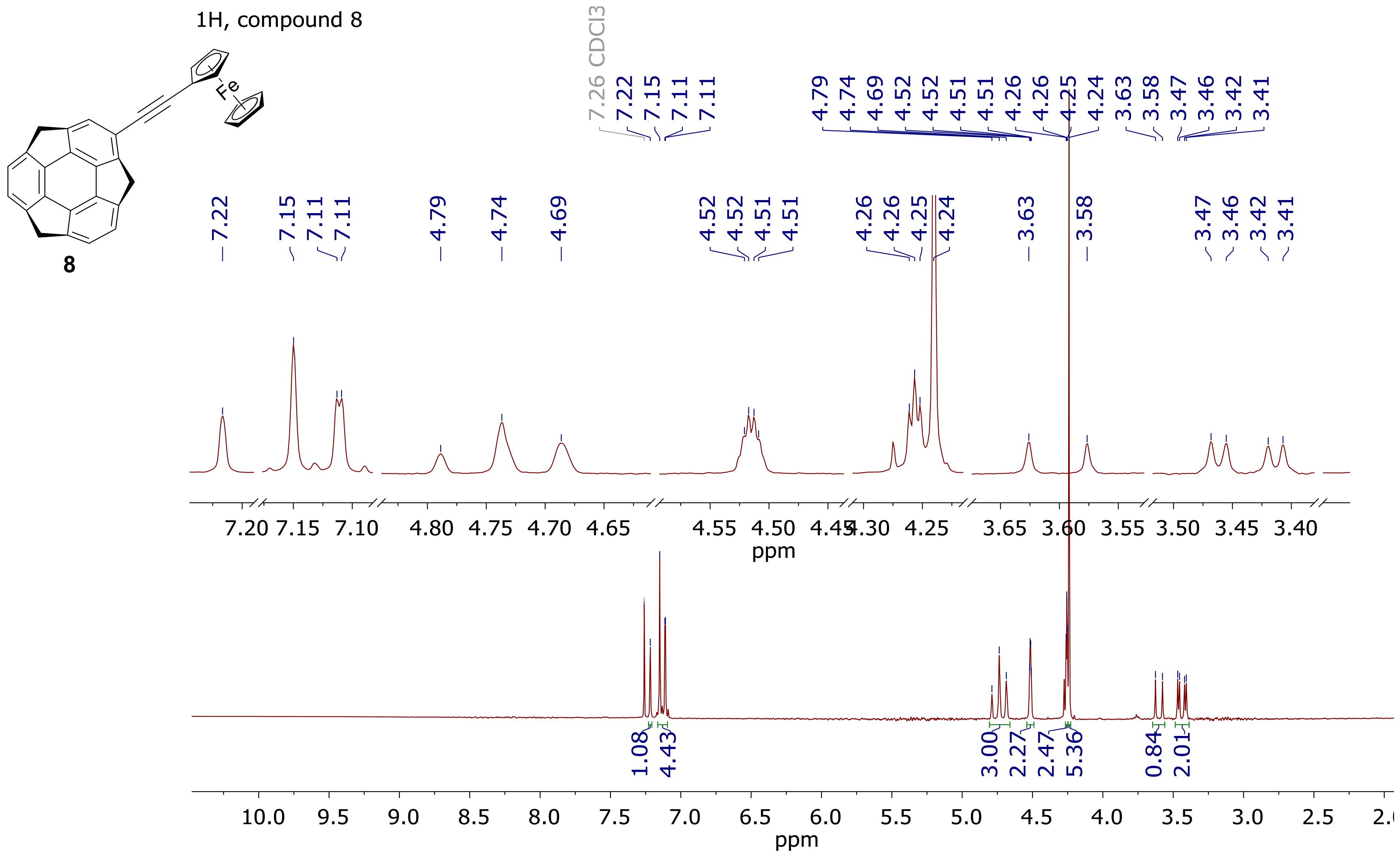

Figure S23. ^{1}H NMR (500 MHz, $CDCl_3$) spectrum of monoferrocenylsumanene **8**.

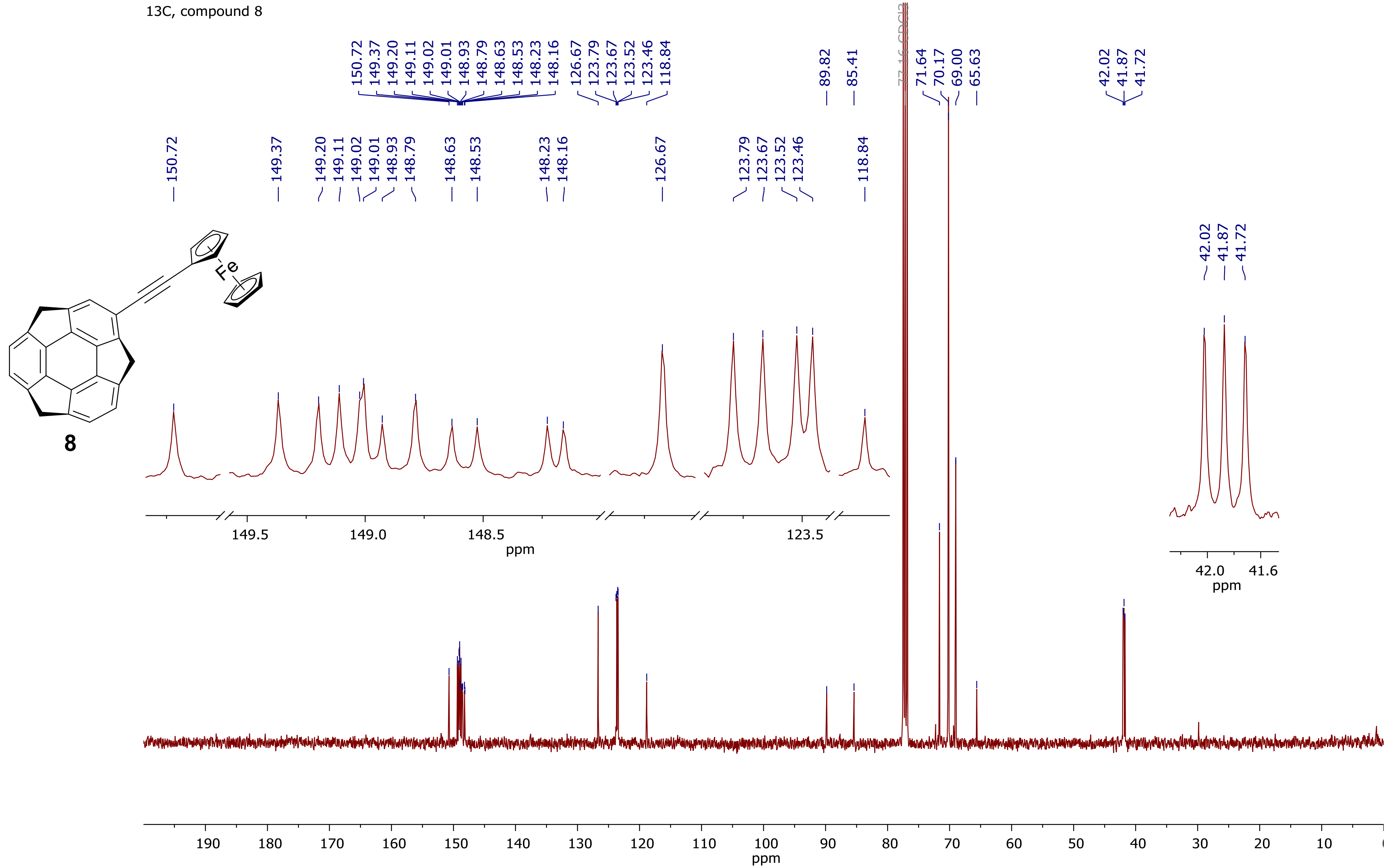

Figure S24. $^{13}C\{^{1}H\}$ NMR (125 MHz, $CDCl_3$) spectrum of monoferrocenylsumanene **8**.

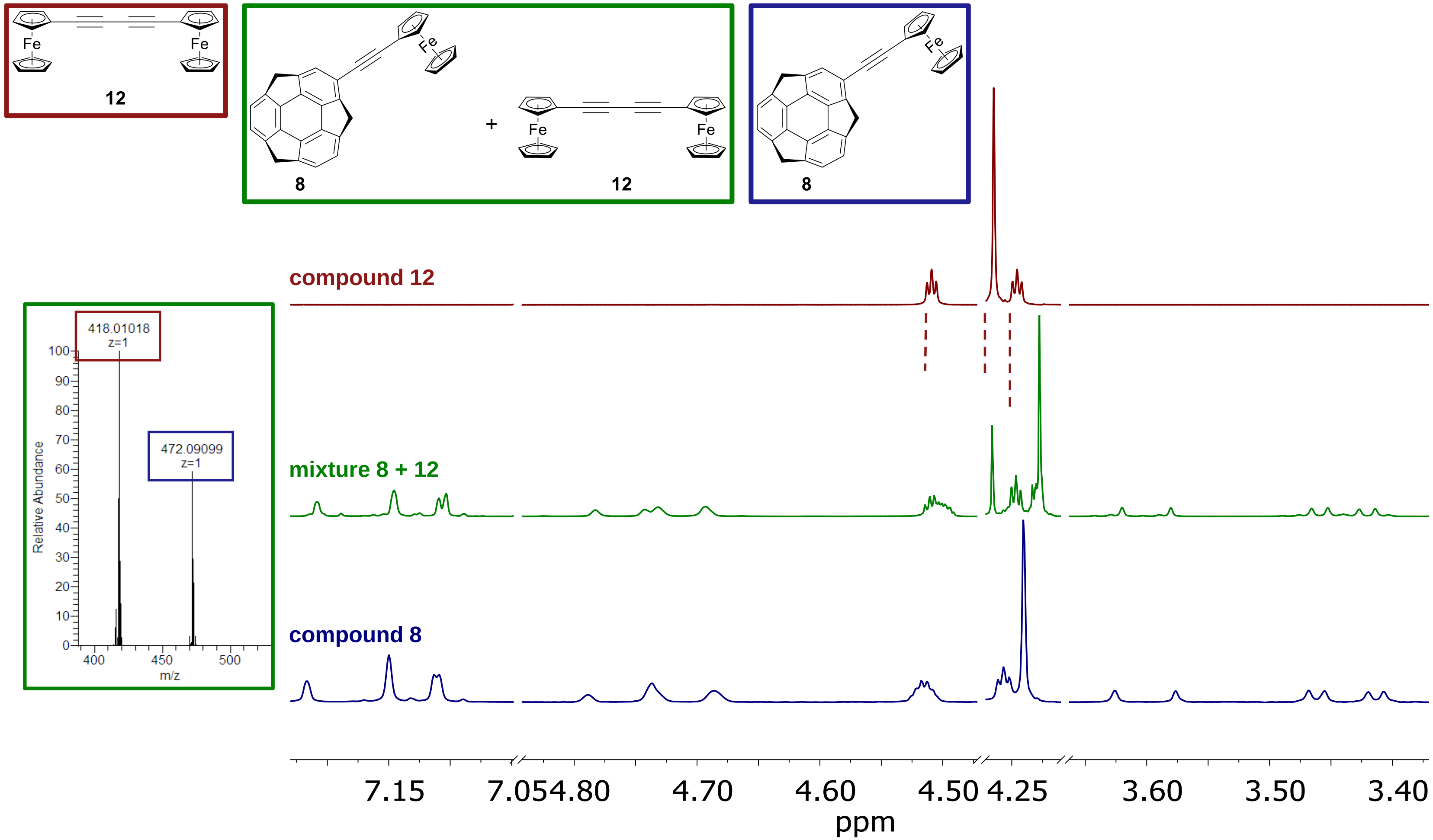

Figure S25. Comparison of ^{1}H NMR spectra (500 MHz) of 1,4-diferrocenylbuta-1,3-diyne (**12**; brown spectrum), the product mixture from the first PTLC purification (green spectrum), and the pure **8** (from the second PTLC purification; blue spectrum). The inset of ESI-HRMS spectrum of the green spectrum sample is also presented. Labels of signals are also presented. The same color does not correspond to the same chemical shift. Selected insets of the spectra are presented. The signals of impurity **12** were clearly seen in the ^{1}H NMR spectrum of the sample obtained from the first PTLC purification process (eluent: 25% CH_2Cl_2/hexane) and the presence of residual **12** in that sample was also detected with high-resolution mass. The second PTLC purification (eluent: 50% THF/hexane) yielded pure **8**.

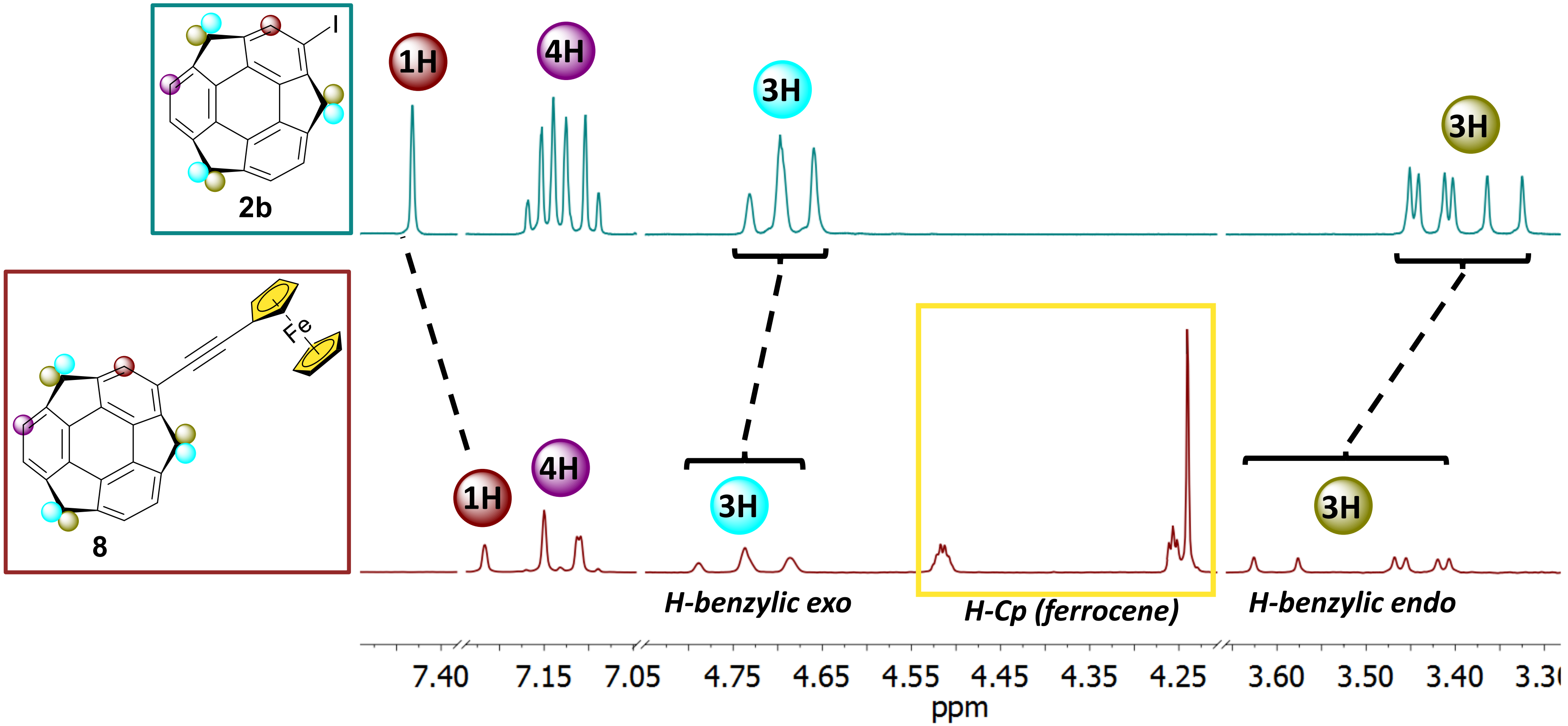

Figure S26. The selected insets of the ^{1}H NMR spectra (500 MHz, $CDCl_3$; 2-iodosumanene **2b** - top and monoferrocenylsumanene **8** - bottom). The signals of the ferrocene protons are marked with the yellow frame. In a relation to the ^{1}H NMR spectrum of compound **2b**, the spectrum of compound **8** showed: (a) signals of the ferrocene protons at 4.52-4.51 ppm and 4.26-4.25 ppm (multiplets, protons from the monosubstituted cyclopentadienyl (Cp) ring), and at 4.24 ppm (singlet, protons from the unsubstituted Cp ring), (b) signal of the sumanene aromatic proton from the substituted ring (brown) less separated from the signals of aromatic protons from the unsubstituted ring (violet), (c) downfield-shifted signals of the benzylic H_{exo} protons (light green) and benzylic H_{endo} protons (dark green). The linking of sumanene **1** with the 2-ferroceneethynyl moiety was also evidenced with the $^{13}C\{^{1}H\}$ NMR spectrum by signals at 89.8 ppm (1xC), 85.4 ppm (1xC), 71.6 ppm (2xC), 70.2 ppm (5xC), 69.0 ppm (2xC) and 65.6 ppm (1xC).

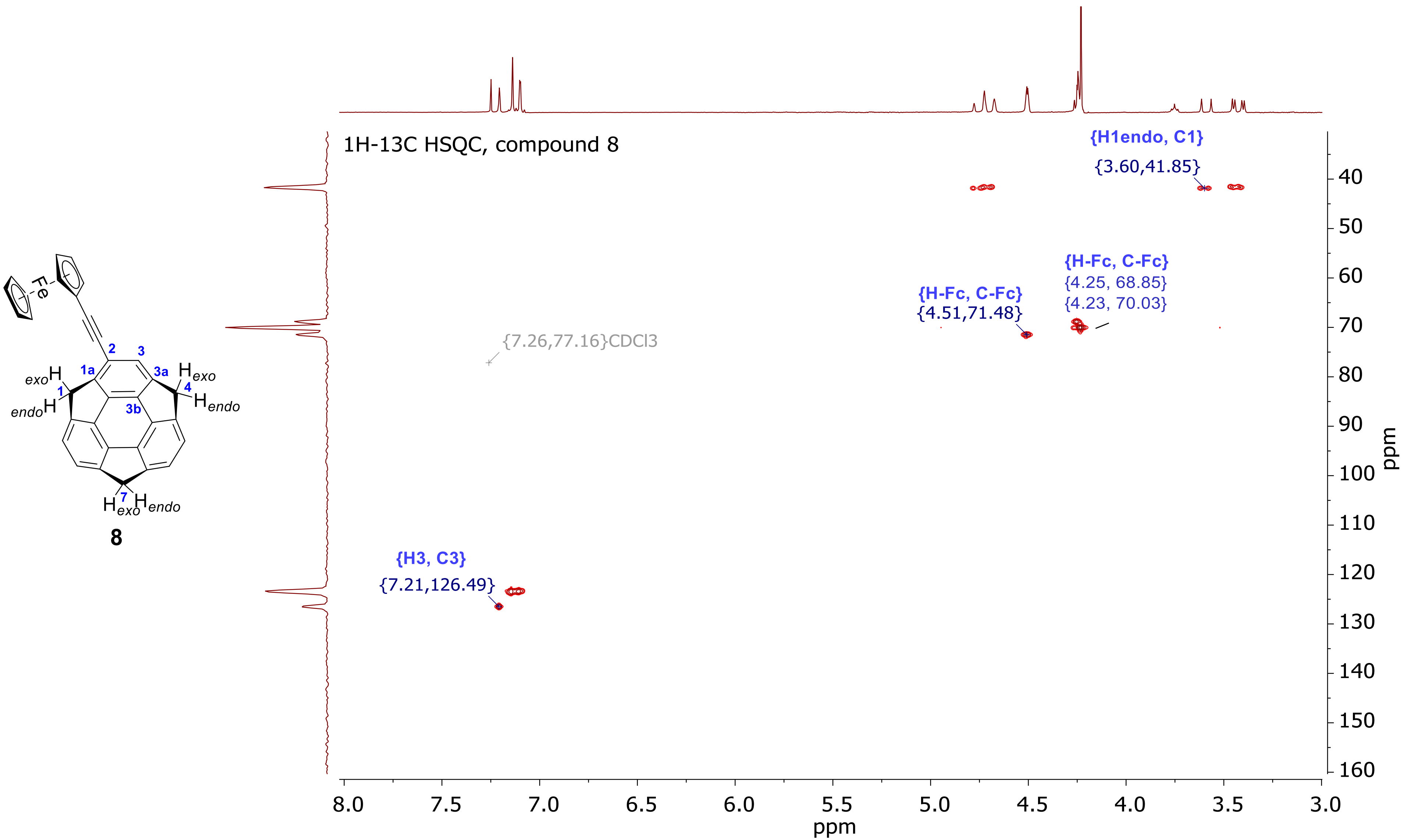

Figure S27. ^{1}H-^{13}C HSQC NMR ($CDCl_3$) spectrum of monoferrocenylsumanene **8**.

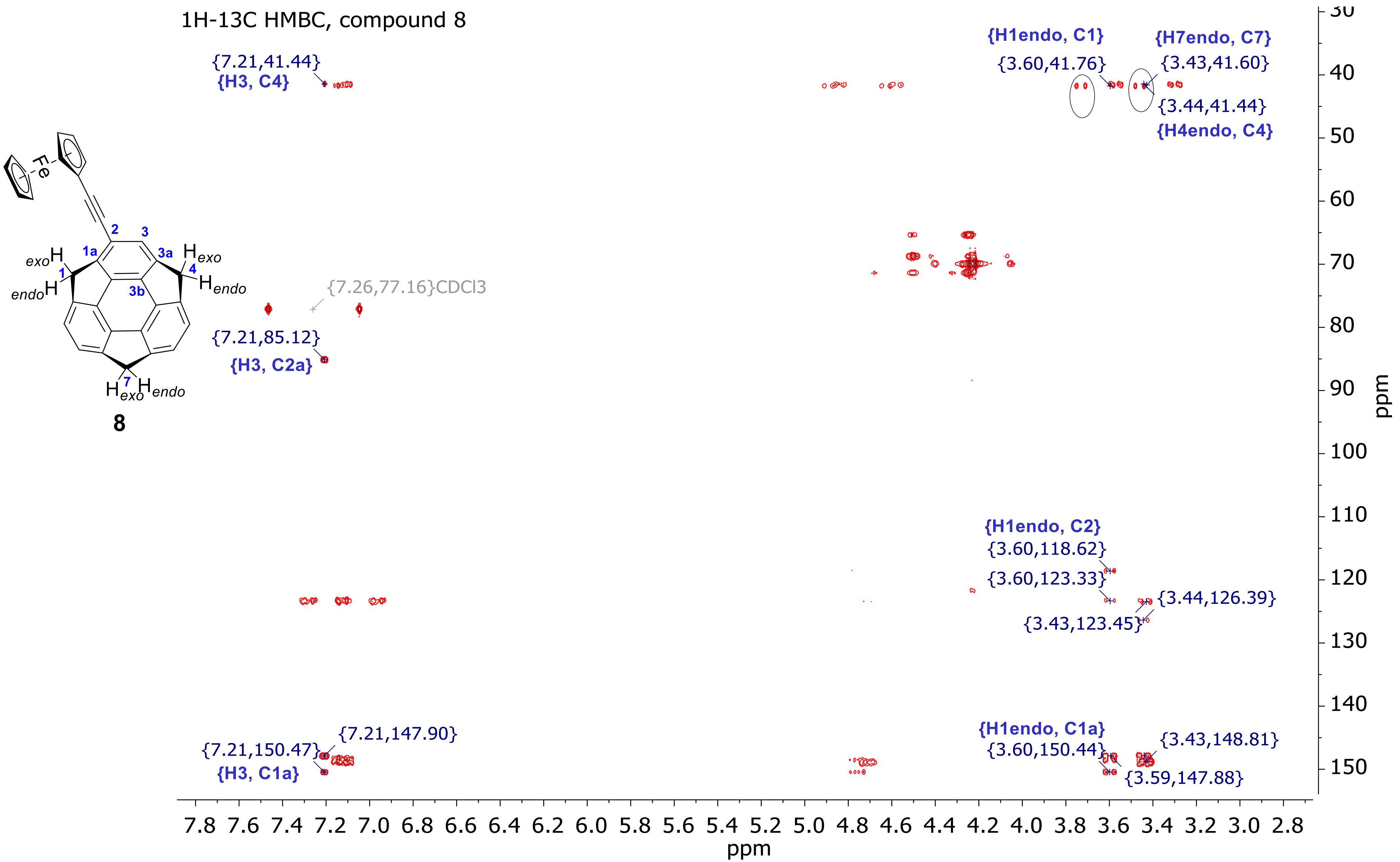

Figure S28. ^{1}H-^{13}C HMBC NMR ($CDCl_3$) spectrum of monoferrocenylsumanene **8**.

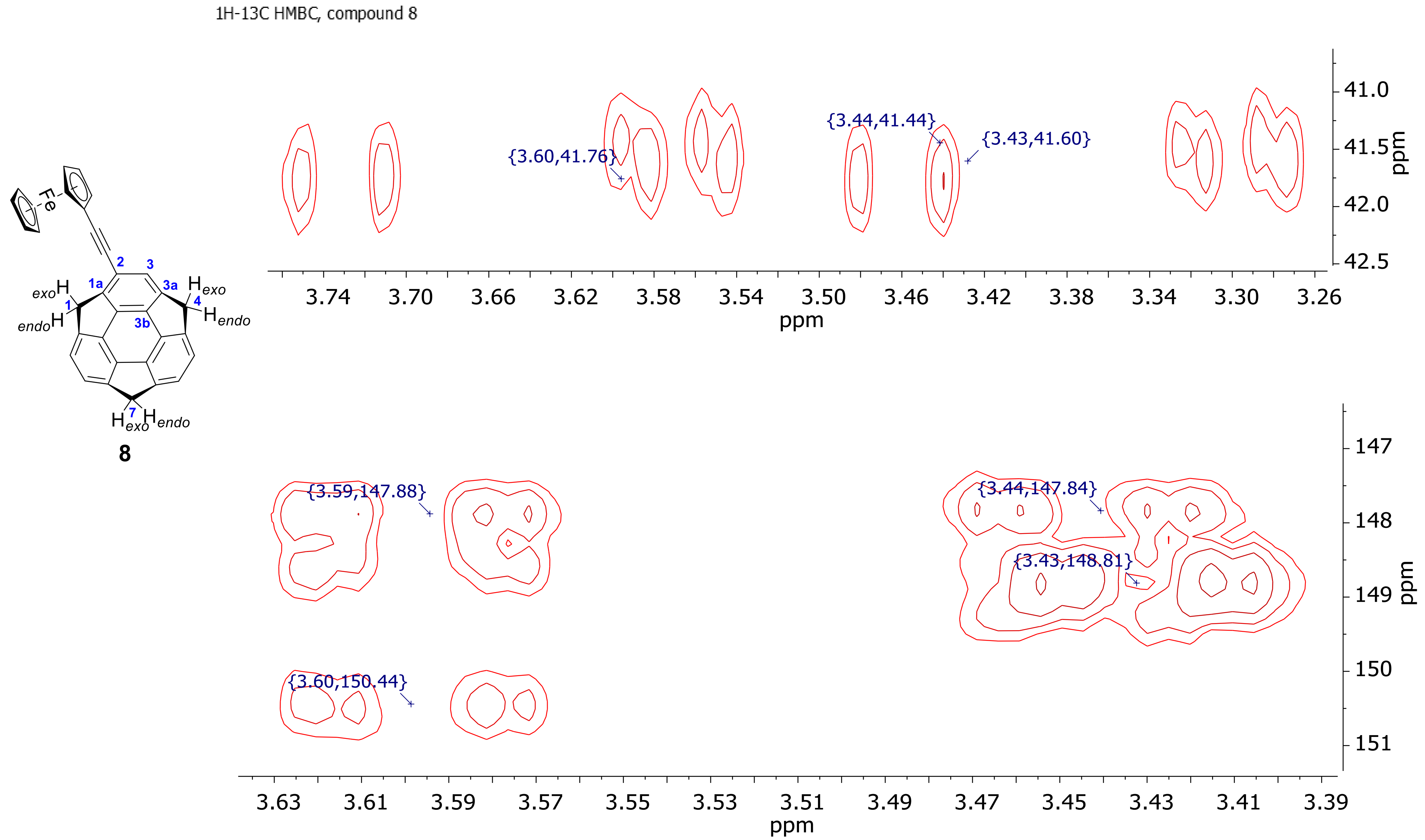

Figure S29. ^{1}H-^{13}C HMBC NMR ($CDCl_3$) spectrum of monoferrocenylsumanene **8** – insets.

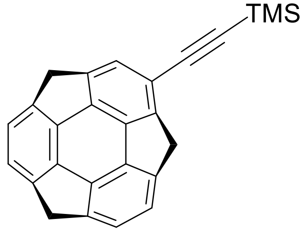

9

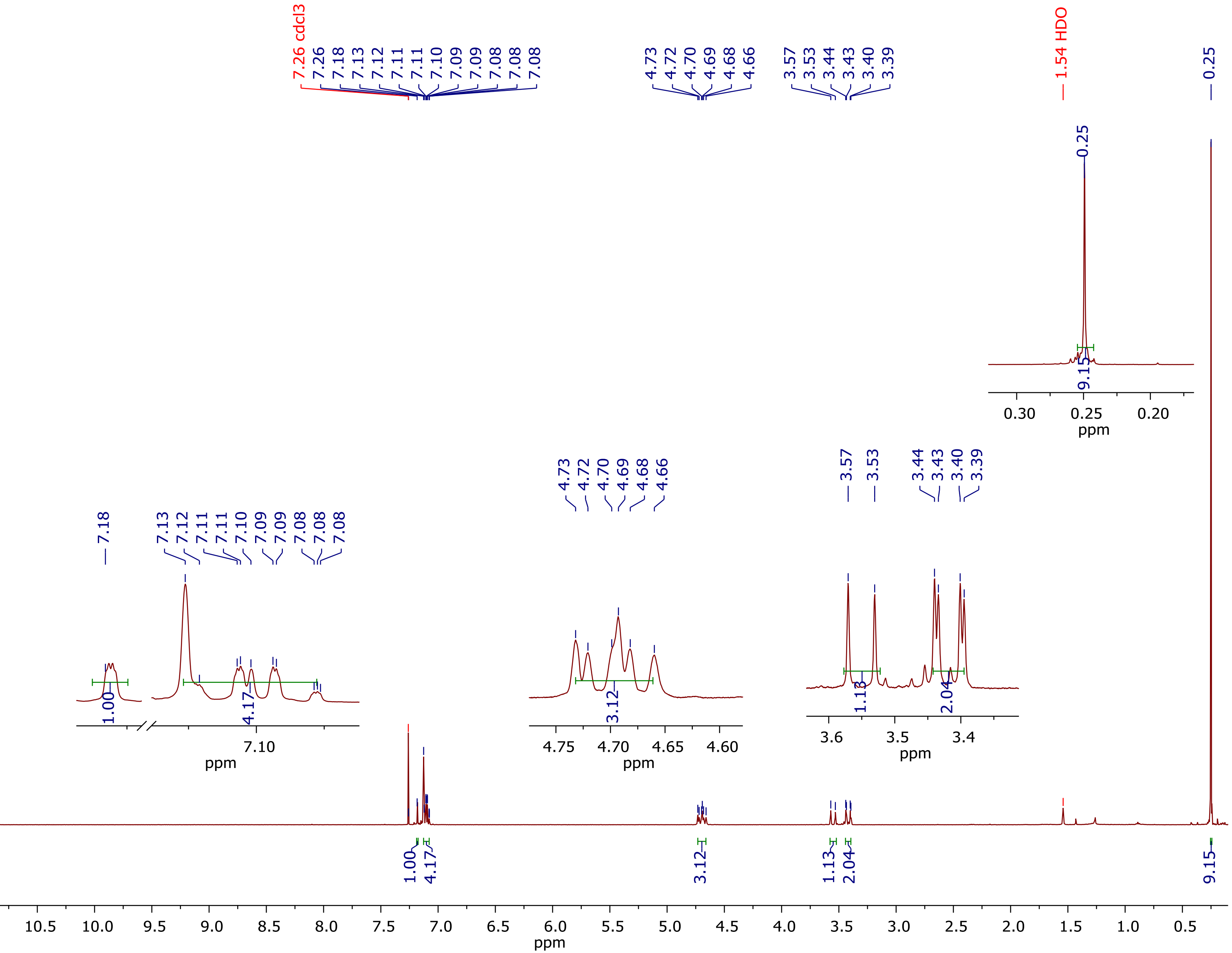

Figure S30. ^{1}H NMR (500 MHz, $CDCl_3$) spectrum of compound **9**.

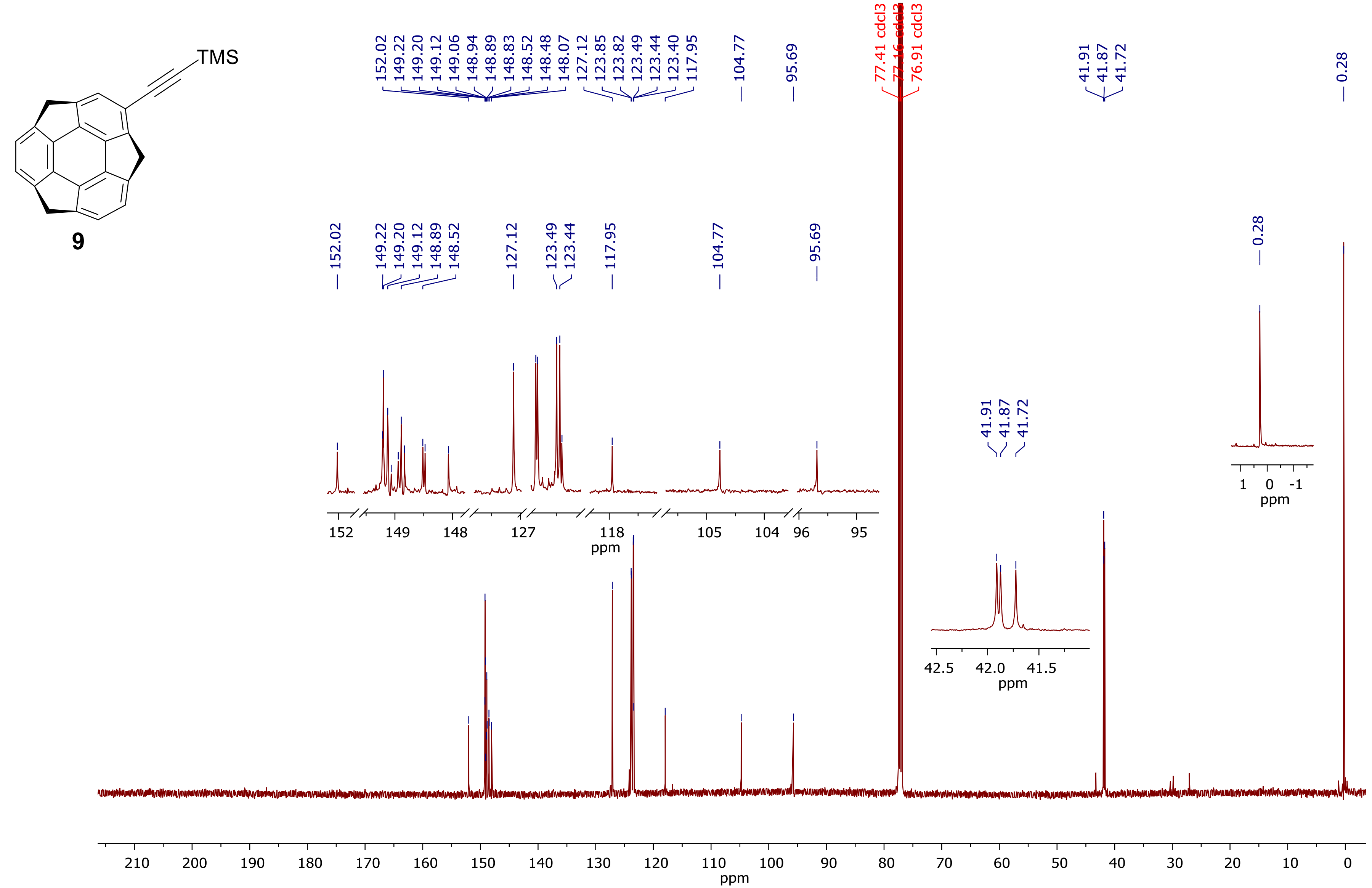

Figure S31. $^{13}C\{^{1}H\}$ NMR (125 MHz, $CDCl_3$) spectrum of compound **9**.

3. Explanation of the specific ^{1}H NMR profiles of compounds 2a, 2b and 6-8

The 1D and 2D NMR spectra were employed in order to explain the specific ^{1}H NMR profiles of compounds **2a**, **2b** and **6-8** (for the corresponding spectra, see Supporting Information, Section 2). Interestingly, in a relation to the ^{1}H NMR profile of sumanene **1**, a signal corresponding to the H3 proton was separated from a set of signals of aromatic protons. Further, signals of the benzylic H1-, H4- and H7 protons were separated in the range of both the H_{endo} and H_{exo} protons (for the numbering system of the examined compounds, see **Table S2**). The most significant changes in the spectra were observed in the rage of signals corresponding to the benzylic H_{endo} protons. For instance, in the spectrum of compound **7**, three well separated doublets were observed. The signal assignments were based on the diagnostic ^{1}H-^{13}C HSQC and ^{1}H-^{13}C HMBC correlations (**Table S2**). Briefly, the applied methodology involved: (a) an identification of the C3 carbon atom signal from the H3↔C3 HSQC correlation (confirmed from the H3↔C3 HMBC correlations in compounds **2a** and **2b**); (b) a localization of both the C4 carbon atom signal from the H3↔C4 HMBC correlation and the $H4_{endo}$ proton signal from the $H4_{endo}$↔C4 HSQC and $H4_{endo}$↔C4 HMBC correlations (signals of $H4_{endo}$ proton and the $H7_{endo}$ proton in the spectra of compounds **7** and **8** were successfully differentiated); (c) a confirmation of the $H4_{endo}$ proton assignment by the $H4_{endo}$↔C3 HMBC correlation in all the examined compounds, accompanied by the $H4_{endo}$↔C3a and H3↔C3a HMBC correlations in compounds **2a** and **2b**; (e) an identification of the $H1_{endo}$ proton signal from two sets of the HMBC correlations: $H1_{endo}$↔C1a and H3↔C1a (compounds **2b** and **6-8**), as well as $H1_{endo}$↔C2 (compounds **2a**, **2b** and **6-8**) and H3↔C2 (compounds **2a**, **2b** and **6**). The $H1_{exo}$↔C1 and $H1_{exo}$↔C1a HMBC correlations in compounds **2a**, **2b** and **7** were also detected. The ^{1}H-^{13}C HMBC correlations within substituents of compounds **6**-**8** are also listed in **Table S2**.

Our analysis revealed the most significant changes in the chemical shifts of signals corresponding to the $H1_{endo}$ proton in the examined compounds, taking the chemical shifts of the H_{endo} protons in sumanene **1** as the reference (**Table S2**). The downfield shift of these signals in the spectrum of compound **2a** ($\delta(H1_{endo})$ = 3.50 ppm) was attributed to the deshielding effect of the highly electronegative bromine atom. On the contrary, the upfield shift of the $H1_{endo}$ proton signals in the spectrum of compound **2b** ($\delta(H1_{endo})$ = 3.36 ppm) was assumed to result from two opposite effects, *i.e.*, a lower electronegativity of the iodine atom (than that of the bromine atom) and the rigid structure of sumanene. Our hypothesis was supported by the literature data where the same tendency was reported for 1-chloro-9*H*-fluorene[6] and 1-iodo-9*H*-fluorene[7] when the ^{1}H NMR chemical shifts of signals corresponding to their benzylic protons were compared with those of 9*H*-fluorene. On the other hand, the ^{1}H NMR chemical shift of a signal corresponding to the benzylic protons in 1-benzyl-2-bromobenzene or 1-benzyl-2-iodobenzene was reported to be essentially constant.[8] Therefore, based on the literature data on the bromine or iodine substituent ^{1}H NMR chemical shift effect in halocyklohexanes[9], we believe that the steric $H1_{endo}\cdots$I shielding might be one of the factors determining the observed $H1_{endo}$ chemical upfield shift in compound **2b**.

Further, the downfield shift of the $H1_{endo}$ proton signals in the spectra of compounds **6**-**8** were observed in a relation to the chemical shift of the H_{endo} protons in sumanene **1**. Our structural optimizations of compounds **6** and **8** suggested a location of the $H1_{endo}$ proton in the deshielding area around alkyne group as a consequence of the bowl shape of the sumanene core (**Figure 4**). Thus, the anisotropic deshielding effect of the alkyne group might be considered one of the factors responsible for chemical shifts of the $H1_{endo}$ proton in compounds

6 and **8** (δ(H1$_{endo}$): 3.58 ppm (**6**) *vs* 3.50 ppm (**1**), and 3.60 ppm (**8**) *vs* 3.50 ppm (**1**)). On the other hand, the strong downfield shift of signals corresponding to the protons H1$_{endo}$, H1$_{exo}$ and H3 the observed in the spectrum of compound **7** (δ(H1$_{endo}$), 3.66 ppm (**7**) *vs* 3.50 ppm (**1**); δ(H1$_{exo}$), 4.95 ppm (**7**) *vs* 4.70 ppm (**1**), and δ(H3), 7.79 ppm (**7**) *vs* 7.18 ppm (**1**)), might be ascribed to a significant electron-withdrawing inductive effect of the 1*H*-1,2,3-triazole ring.[10]

The abovementioned electron-withdrawing inductive effect of the 1*H*-1,2,3-triazole ring could also be considered responsible for the observed differences in the ^{1}H NMR pattern of signals corresponding to the aromatic protons of H5, H6, H8 and H9 spectrum of compounds **7** and **6** (see, **Figure S19**, and **Figure S17** *vs* **Figure S11**). Based on the corresponding ^{1}H-^{13}C$_{ipso}$ HMBC correlations in the spectrum of compound **7** (**Figure 2**, **Figure S17** and **Table S2**), the following assignments were found: H5 (7.18) and C5 (123.8); H6 (7.20) and C6 (124.5); H8 (7.20) and C8 (123.7); H9 (7.18) and C9 (123.8). The diagnostic ^{1}H-^{13}C HMBC correlations of H1$_{endo}$↔C9, H4$_{endo}$↔C5, H7$_{endo}$↔C6 and H7$_{endo}$↔C8 were crucial. Interestingly, the signal corresponding to the C6 carbon atom (δ 124.1 ppm) featured a significant downfield shift in a relation to the chemical shift of signals corresponding to the carbon atoms of C5, C8 and C9 (δ 123.73-123.79 ppm). Again, this phenomenon was attributed to the electron-withdrawing inductive effect of the 1*H*-1,2,3-triazole ring decreasing an electron density around the C6 carbon nuclei. Indeed, literature data showed a decreasing effect of the electron-withdrawing substituents on an electron density around the C6 carbon nuclei of the 2-nitro-, 2-formyl- or 2-acylsumanenes.[4]

Table S2. The NMR signal assignments and diagnostic 2D NMR correlations in compounds **2a**, **2b**, **6-8**.[a,b]

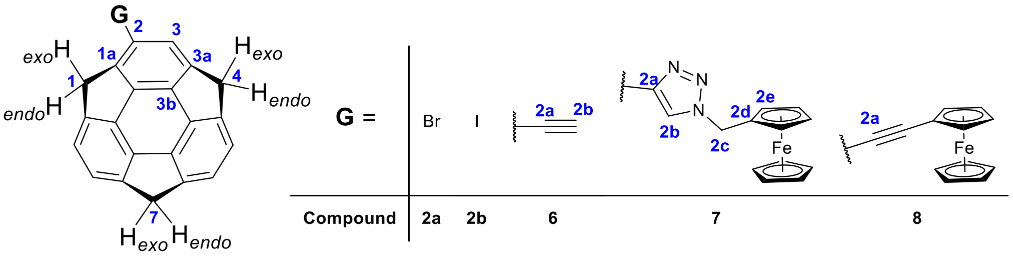

Compound<br>Solvent	**A. Diagnostic ^{1}H NMR signals/H (δ/ppm, multiplicity, $^{2}J_{H-H}$ [c]/Hz)**<br>**B. Diagnostic ^{13}C NMR signals/C (δ/ppm)**<br>**C. Diagnostic ^{1}H-^{13}C HSQC correlations**<br>**D. Diagnostic ^{1}H-^{13}C HMBC correlations**
**2a**<br>$CDCl_3$	**A.** H1$_{endo}$ (3.50, d, $^{2}J_{H-H}$ 19.6), H1$_{exo}$ (4.65[d]), H3 (7.21, s), H4$_{endo}$ and H7$_{endo}$ (3.44, d, $^{2}J_{H-H}$ 19.5)[e]<br>**B.** C1 (43.3), C2 (116.7), C3 (127.0), C3a (151.8), C4 and C7 (41.7 and 41.9)[d]<br>**C.** H1$_{endo}$↔C1, H1$_{exo}$↔C1, H3↔C3<br>**D.** H1$_{endo}$↔C1, H1$_{endo}$↔C2, H1$_{exo}$↔C1, H3↔C2, H3↔C3, H3↔C3a, H3↔C4, H4$_{endo}$↔C3, H4$_{endo}$↔C3a
**2b**<br>$CDCl_3$	**A.** H1$_{endo}$ (3.34,d, $^{2}J_{H-H}$ 19.6), H1$_{exo}$ (4.65[d]), H-3 (7.43, s), H4$_{endo}$ and H7$_{endo}$ (3.43, d, $^{2}J_{H-H}$ 19.6 and 3.42,d, $^{2}J_{H-H}$ 19.6)[e]<br>**B.** C1 (46.2), C1a (153.2), C2 (89.4), C3 (132.3), C3a (151.6), C4 and C7 (41.4 and 41.9)[e]<br>**C.** H1$_{endo}$↔C1, H1$_{exo}$↔C1, H3↔C3<br>**D.** H1$_{endo}$ ↔C1, H1$_{endo}$↔C1a, H1$_{endo}$↔C2, H1$_{exo}$↔C1, H3↔C1a, H3↔C2, H3↔C3, H3↔C3a, H3↔C4, H4$_{endo}$↔C3, H4$_{endo}$↔C3a
**6**<br>$CDCl_3$	**A.** H1$_{endo}$ (3.58, d, $^{2}J_{H-H}$ 20.0), H3 (7.21, s), H4$_{endo}$ and H7$_{endo}$ (3.44, d, $^{2}J_{H-H}$ 19.6 and 3.43, d, $^{2}J_{H-H}$ 19.5)[e]<br>**B.** C1a (152.2), C2 (116.7); C2a (83.4), C2b (78.3), C3 (127.4)<br>**C.** H1$_{endo}$↔C1, H2b↔C2a, H2b↔C2b, H3↔C3<br>**D.** H1$_{endo}$↔C1, H1$_{endo}$↔C1a, H1$_{endo}$↔C2, H2b↔C1a, H2b↔C2a, H2b↔C2b, H2b↔C3, H3↔C1a, H3↔C2, H3↔C2a,H3↔C3, H3↔C4, H4$_{endo}$↔C3
**7**<br>DMSO-d_6	**A.** H1$_{endo}$ (3.66, d, $^{2}J_{H-H}$ 20.5)**,** H1$_{ex\ o}$(4.95[d])**,** H2b (8.57, s), H2c (5.37, s), H3 (7.79, s), H4$_{endo}$ (3.57, d, $^{2}J_{H-H}$ 19.7), H4$_{exo}$ (4.70), H5 (7.18), H6 (7.20), H7$_{endo}$ (3.50, d, $^{2}J_{H-H}$ 19.7), H7$_{exo}$ (4.74[d]), H8 (7.20), H9 (7.18),<br>**B.** C1 (42.7), C1a (144.2), C2 (126.8), C2a (146.1), C2b (122.1), C2c (49.1), C2d (82.5), C2e (68.7), C3 (120.9), C3a (147.1 or 149.6), C3b (149.6 or 147.1), C4 (41.3), C5 (123.8), C6 (124.5), C7 (41.0[d]), C8 (123.7), C9 (123.8)<br>**C.** H1$_{endo}$↔C1, H1$_{exo}$↔ C1, H2b↔C2b, H2c↔C2c, H2e↔C2e, H3↔C3,<br>**D.** H1$_{endo}$↔C1, H1$_{endo}$↔C1a, H1$_{endo}$↔C2, H1$_{endo}$↔C9, H1$_{exo}$↔C1, H1$_{exo}$↔C1a, H2b↔C2a, H2b↔C2b, H2c↔C2b, H2c↔C2c, H2c↔C2d, H2c↔C2e, H3↔C1a, H3↔C2a, H3↔C3a or C3b, H3↔C3b or C3a, H3↔C4, H4$_{endo}$↔C3, H4$_{endo}$↔ C3a or C3b, H4$_{endo}$↔ C3b or C3a, H4$_{endo}$↔C4, H4$_{endo}$↔C5, H5↔C5, H6↔C6, H7$_{endo}$↔C7, H7$_{endo}$↔C6, H7$_{endo}$↔C8, H8↔C8, H9↔C9,
**8**<br>$CDCl_3$	**A.** H1$_{endo}$ (3.60, d, $^{2}J_{H-H}$ 19.7), H3 (7.22, s), H4$_{endo}$ (3.44, d, $^{2}J_{H-H}$ 19.4), H7$_{endo}$ (3.43, d, $^{2}J_{H-H}$ 19.3)<br>**B.** C1 (42.0), C2 (118.8), C2a (85.4), C3 (126.7[d]), C4 (41.7), C7 (41.9)<br>**C.** H1$_{endo}$↔C1, H3↔C3<br>**D.** H1$_{endo}$↔C1, H1$_{endo}$↔C1a, H1$_{endo}$↔C2, H3↔C1a, H3↔C2a, H3↔C3, H3↔C4, H4$_{endo}$↔C3, H4$_{endo}$↔C4, H7$_{endo}$↔C7

[a] **1** ($CDCl_3$) δ(H1$_{endo}$, H4$_{endo}$ and H7$_{endo}$) 3.42 (d, $^{2}J_{H-H}$ 18.1); **1** (DMSO-d_6) δ(H1$_{endo}$, H4$_{endo}$ and H7$_{endo}$) 3.50 (d, $^{2}J_{H-H}$ 18.8). [b] Otherwise noted, δ(H) or δ(C) from the corresponding 1D NMR spectra is given. [c] When applicable. [d] From ^{1}H-^{13}C HMBC NMR spectrum. [e] Precise chemical shifts not distinguished.

4. Calculations

Otherwise noted, all structure optimizations, self-consistent field (SCF) energies, and thermal energy correction calculations using density functional theory (DFT) were performed using Gaussian 16 suite of programs (revision C.01)[11] at ωB97X-D[12] level of theory in gas phase with Def2-SVP[13] as a basis set. The DFT optimized structures of 2-ethynylsumanene (**6**), monoferrocenylsumanenes **7** and **8** at ωB97X-D/Def2-SVP level of theory, are presented in **Figure S32-S36**. **Figure S37** presents the differences in bowl depth values for those derivatives. The introduction of the substituents to the sumanene (**1**) molecule slightly affected the observed bowl depth. The bowl depth for newly synthesized **6**, **7** and **8** was defined as the distance between the plane formed by the six-membered ring in the center of the sumanene skeleton and the rim carbon[14,15], see **Figure S37**a. The bowl depth values were taken as the values for the carbon atom at the substituted aromatic ring of sumanene[15] (marked with a black arrow in **Figure S37**c). This way, the bowl depth for compounds **6**, **7** and **8** equaled to 1.18, 1.16 and 1.19 Å, respectively. Those values are slightly higher than the bowl depth for the unmodified sumanene (1.15 Å, estimated from the DFT optimized structure; **Figure S37**b)[14,15]. It suggested that introducing acetylene or 1,2,3-triazole moieties to the aromatic sumanene ring might cause a slight distortion of bowl structure.[15]

Optimized Cartesian coordinates of sumanene **1**

C	-1.34950600	-3.09750600	0.44981600
C	-2.95055900	1.64647500	0.44972400
C	0.04932600	-3.37840900	0.44990500
C	0.97133400	-2.46376500	-0.06762200
C	0.43608500	-1.34018100	-0.70061700
C	2.46780000	-2.17190500	0.18742500
C	-2.61938900	0.39069200	-0.06772300
C	3.35749700	0.38006700	0.44934600
C	-3.11489800	-1.05117300	0.18735800
C	-2.00791700	2.71749600	0.44980600
C	-0.72020900	2.54849200	-0.06760400
C	-1.84698700	-1.89788500	-0.06770600
C	-0.91957400	-1.06800800	-0.70063300
C	2.56717400	-0.65056300	-0.06796800
C	1.38463600	-0.26236400	-0.70074300
C	-1.37866300	0.29237100	-0.70066400
C	2.90130100	1.73195100	0.44948000
C	0.64710800	3.22305600	0.18762300
C	-0.46513800	1.33035900	-0.70060500
C	1.64802600	2.07304400	-0.06771300
C	0.94251700	1.04777100	-0.70066600
H	-2.00542900	-3.78794700	0.98706500

H	-3.89071800	1.79832800	0.98697100
H	0.38779300	-4.26854600	0.98719000
H	3.12192400	-2.74775900	-0.48971900
H	4.28349500	0.15724700	0.98644400
H	-2.27795900	3.63070600	0.98711600
H	3.50301200	2.47015400	0.98667600
H	0.81862400	4.07745600	-0.48957800
H	-3.94047500	-1.32979200	-0.48999000
H	2.76209600	-2.43074400	1.21549500
H	-3.48648100	-1.17655200	1.21533700
H	0.72433400	3.60735400	1.21567800

Imaginary frequency = zero

Sum of electronic and thermal Free Energies = –806.370027 Hartree

Optimized Cartesian coordinates of compound **6**

C	-0.23840200	3.37643300	0.35386000
C	2.94755600	-0.47023900	0.00100300
C	-1.63581800	3.13594000	0.50977400
C	-2.22219600	1.95119000	0.05461800
C	-1.39544700	1.09524100	-0.67551900
C	-3.47662500	1.14114800	0.45379100
C	2.12211600	0.58142500	-0.43577100
C	-3.35917500	-1.55965600	0.70678800
C	2.09914900	2.09883100	-0.16627400
C	2.44507800	-1.81670100	0.05644200
C	1.13675600	-2.11684300	-0.31712800
C	0.59385600	2.43573800	-0.26023300
C	-0.04120000	1.32946100	-0.82790000
C	-3.05345100	-0.31414000	0.15013000
C	-1.88950200	-0.25151100	-0.61830100
C	0.86936000	0.22371200	-0.92991000
C	-2.45325300	-2.65727300	0.60627700
C	0.13945300	-3.23907900	0.05049700
C	0.39294300	-1.07267100	-0.87341400
C	-1.22782200	-2.52562800	-0.05317900
C	-1.01193500	-1.31600000	-0.71679600
H	0.18344500	4.25536600	0.84865200
H	-2.20773100	3.84451500	1.11501900

H	-4.36513100	1.44235500	-0.12737300
H	-4.23677300	-1.68396700	1.34699200
H	3.08592000	-2.56450300	0.52951500
H	-2.68666800	-3.56143700	1.17492800
H	0.21157500	-4.09642500	-0.64043700
H	2.70197100	2.65712800	-0.90260700
H	-3.72623600	1.27748700	1.51658600
H	2.51354200	2.33258100	0.82596000
H	0.32047600	-3.62603800	1.06421300
C	4.24309400	-0.18924700	0.55270200
C	5.33285200	0.06590600	1.01596600
H	6.30127500	0.28673200	1.42401300

Imaginary frequency = zero
Sum of electronic and thermal Free Energies = –882.424555 Hartree

Optimized Cartesian coordinates of compound **8**

C	3.43482800	0.14697600	1.21522300
C	4.10053400	1.41874300	1.18610600
H	3.67614000	2.34031800	0.79381300
C	5.39804800	1.24819800	1.74239300
H	6.15259500	2.02516500	1.84783000
C	5.54735300	-0.12010100	2.11568300
H	6.43579400	-0.56897300	2.55533900
C	4.34287300	-0.80334000	1.79276000
H	4.13394900	-1.86051000	1.94095500
C	4.59087100	-0.60861300	-1.75699000
H	3.56707500	-0.85600300	-2.03319500
C	5.54604800	-1.50333100	-1.19278900
H	5.38589500	-2.55930100	-0.98365700
C	6.73612300	-0.76543500	-0.92245400
H	7.64598000	-1.15995500	-0.47398500
C	6.51587000	0.58576900	-1.32029100
H	7.22812700	1.40363600	-1.22861600
C	5.18968900	0.68210200	-1.83627000
H	4.70975900	1.58681100	-2.20453500
C	2.11631600	-0.12560600	0.74282200
C	0.99601100	-0.36265100	0.33788400
Fe	5.14942800	0.03129600	0.10653900
C	-0.32909700	-0.61489200	-0.14344000

C	-1.12988100	0.44796000	-0.60149900
C	-0.88655000	-1.93418000	-0.00224100
C	-2.41529400	0.12186400	-1.02770300
C	-2.21996300	-2.19482400	-0.31054200
H	-0.26041600	-2.68520100	0.48526800
C	-3.27455700	1.26963900	-0.94377200
C	-2.94265500	-1.14877000	-0.89098900
C	-2.57153600	2.37284500	-0.45529800
C	-4.63005000	1.10202700	-0.72724000
C	-4.34844900	-1.32257200	-0.66505300
C	-3.33805400	3.37651100	0.14455500
C	-5.17708500	-0.21791400	-0.58526900
C	-5.38975200	2.02654200	-0.00776400
C	-4.58665900	-2.48820300	0.06637500
C	-4.73666000	3.20469700	0.36628500
H	-2.85988200	4.25808300	0.57992400
C	-6.30942000	-0.19345600	0.23110900
C	-5.78777800	-2.53503300	0.77974900
H	-5.25332400	3.96480400	0.95857900
C	-6.64309100	-1.39604600	0.86109100
H	-6.03380700	-3.39977400	1.40195100
H	-7.49752500	-1.45103100	1.54110400
C	-6.65913000	1.29144100	0.47942000
H	-6.85888200	1.48859600	1.54322900
H	-7.55756900	1.60312400	-0.08061600
C	-1.07904000	1.97536500	-0.40257500
H	-0.61783700	2.23383100	0.56273000
H	-0.48254300	2.47517500	-1.18481300
C	-3.24659300	-3.25376400	0.15218300
H	-3.03985500	-3.59775500	1.17644600
H	-3.23843100	-4.14643800	-0.49651600

Imaginary frequency = zero
Sum of electronic and thermal Free Energies = –2531.392266 Hartree

Optimized Cartesian coordinates of compound **7**

C	-4.05899000	0.02009500	-0.12552000
C	-4.13595500	0.34062600	1.26559100
H	-3.85460900	-0.31910300	2.08480400
C	-4.67423800	1.65186700	1.38593800

H	-4.88528200	2.17167900	2.31846500
C	-4.92386400	2.15204400	0.07477900
H	-5.35784400	3.11995100	-0.16831700
C	-4.53583800	1.15124200	-0.85987700
H	-4.61944800	1.21445300	-1.94409500
C	-7.25033900	-0.91369400	-0.52977800
H	-7.00351500	-1.52211300	-1.39817500
C	-7.80954000	0.39699000	-0.55748300
H	-8.05965100	0.96657400	-1.45056500
C	-7.95718500	0.84136900	0.78811700
H	-8.33870100	1.81065900	1.10342400
C	-7.48660100	-0.19317700	1.64748800
H	-7.44608500	-0.15292400	2.73435100
C	-7.05089700	-1.27883600	0.83363400
H	-6.62347600	-2.21409100	1.19106500
Fe	-5.98568000	0.41775400	0.38810200
C	-3.54842700	-1.25815300	-0.70824800
H	-3.93578100	-1.39197700	-1.72982300
H	-3.86283800	-2.12574800	-0.11439800
N	-2.09277200	-1.30166600	-0.74099400
C	-1.21440800	-0.29300700	-0.93079800
C	0.02245800	-0.91524500	-0.92636100
H	-1.53302100	0.73911800	-1.03628300
N	-1.46283700	-2.46824700	-0.62808400
N	-0.19683900	-2.24738900	-0.73993300
C	1.37396500	-0.35892800	-1.07959300
C	2.50570800	-1.18897400	-0.97725900
C	1.53609300	1.06263100	-1.19289000
C	3.74452500	-0.57179300	-1.15104900
C	2.79426200	1.66183300	-1.21951600
H	0.63881600	1.68328700	-1.12292300
C	4.78562500	-1.34894000	-0.54034600
C	3.88862100	0.79729000	-1.27277300
C	4.25394200	-2.49358000	0.05502300
C	5.92504200	-0.72611500	-0.06388100
C	5.06742800	1.44497400	-0.77430500
C	5.01630000	-3.06252400	1.08070600
C	6.06768600	0.69735800	-0.18097300

C	6.62256200	-1.19813500	1.04905400
C	4.77894700	2.75284600	-0.37919600
C	6.18976300	-2.41964900	1.57474000
H	4.65540000	-3.94308100	1.61912200
C	6.85966200	1.19840100	0.85388200
C	5.64814500	3.32030100	0.55707600
H	6.66373700	-2.84304200	2.46461300
C	6.68133300	2.54886300	1.16877600
H	5.47335500	4.32897400	0.94141600
H	7.24074300	3.00954500	1.98761400
C	2.77213700	-2.58176200	-0.36647100
H	2.10452800	-2.77409500	0.48481900
H	2.57322700	-3.39028700	-1.08891300
C	3.33747200	3.06086700	-0.84715000
H	3.32185200	3.75253500	-1.70699300
H	2.74262000	3.52733100	-0.04767400
C	7.45944500	-0.01842400	1.59548800
H	7.35615100	0.08095000	2.68640700
H	8.53560400	-0.14103700	1.38352600

Imaginary frequency = zero

Sum of electronic and thermal Free Energies = –2735.326215 Hartree

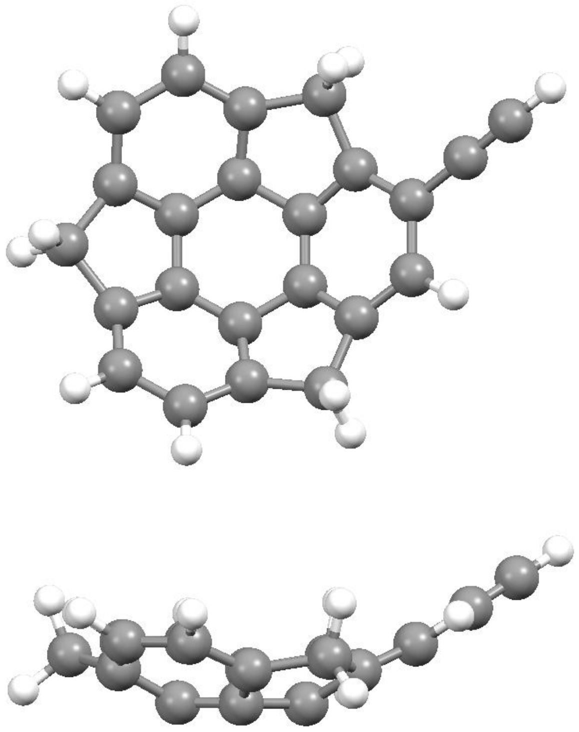

Figure S32. DFT-optimized structure of the compound **6** viewed from two different perspectives.

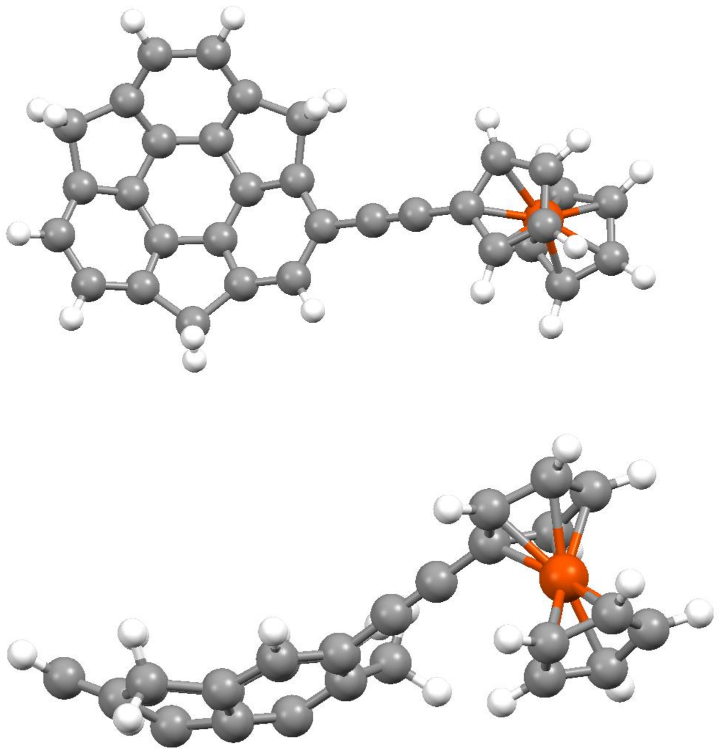

Figure S33. DFT-optimized structure of the monoferrocenylsumanene **8** viewed from two different perspectives.

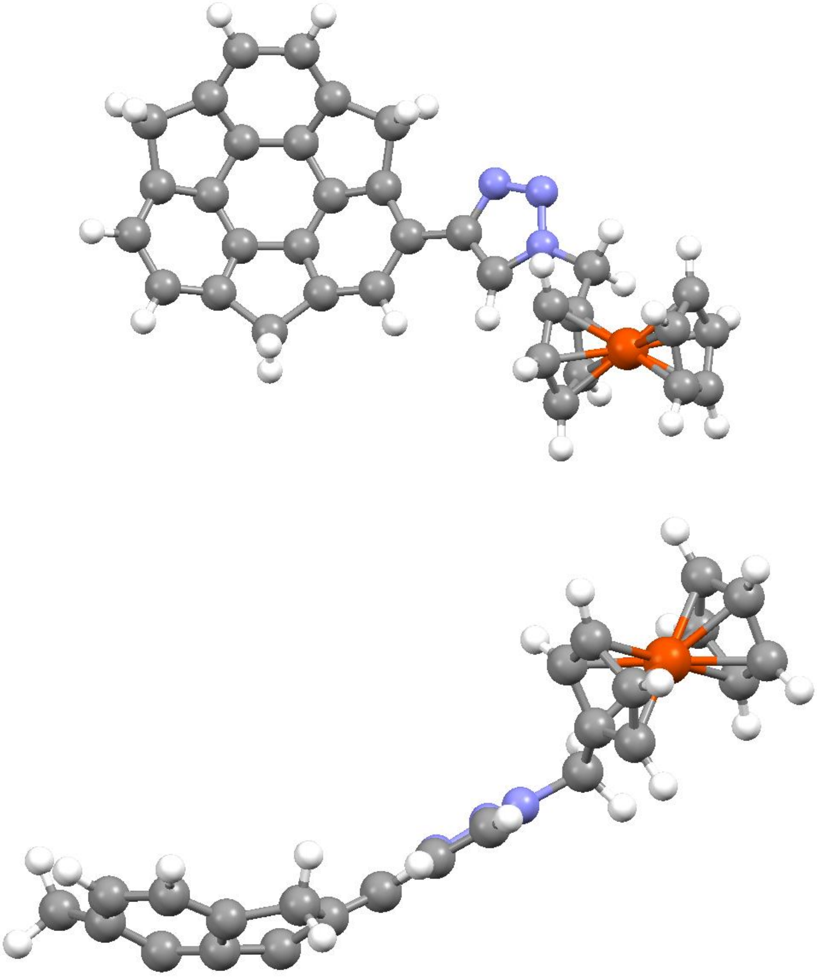

Figure S34. DFT-optimized structure of the monoferrocenylsumanene **7** viewed from two different perspectives.

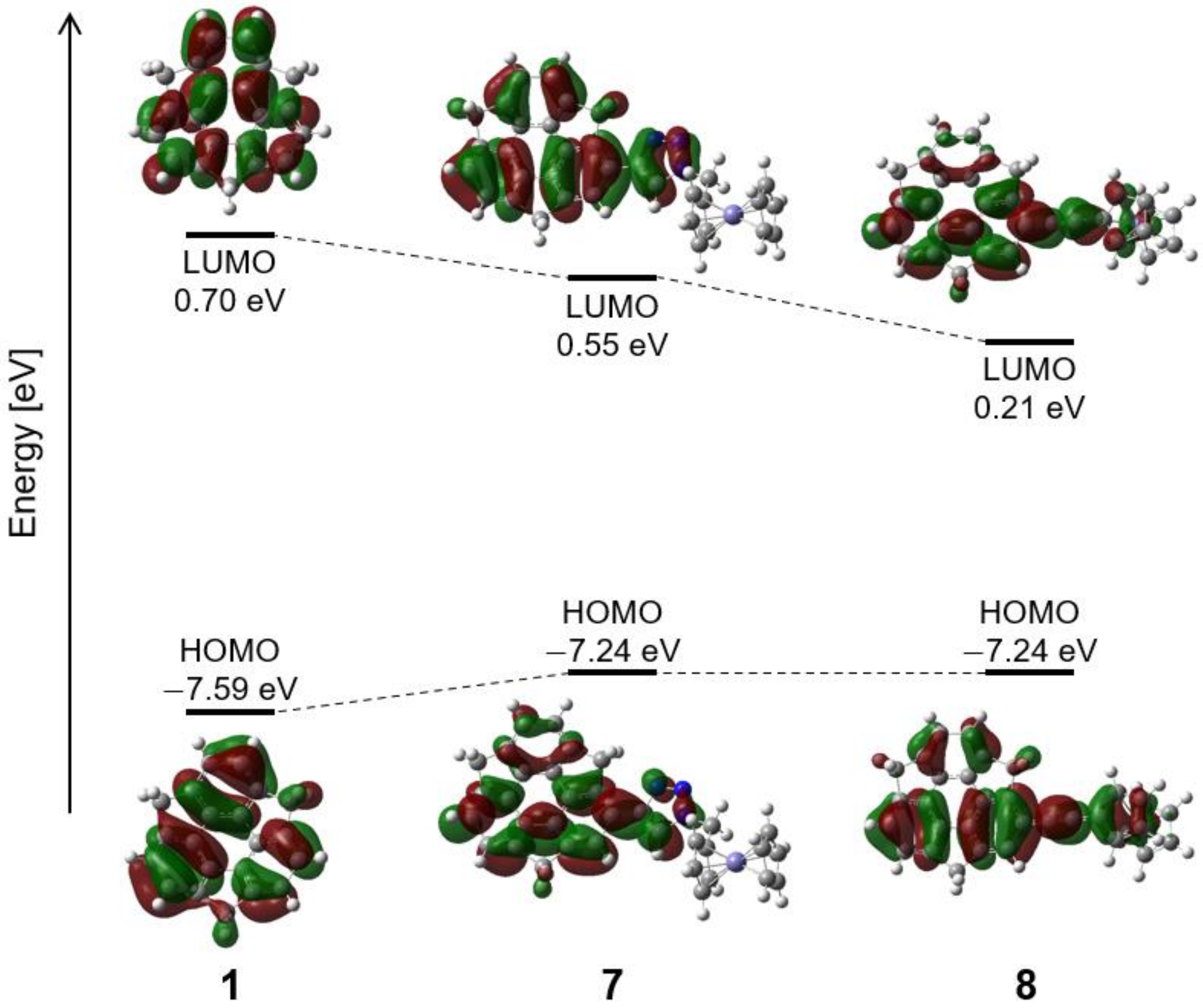

Figure S35. Calculated energy level and Kohn‑Sham orbitals at the HOMO and LUMO of **1**, **7**, and **8**.

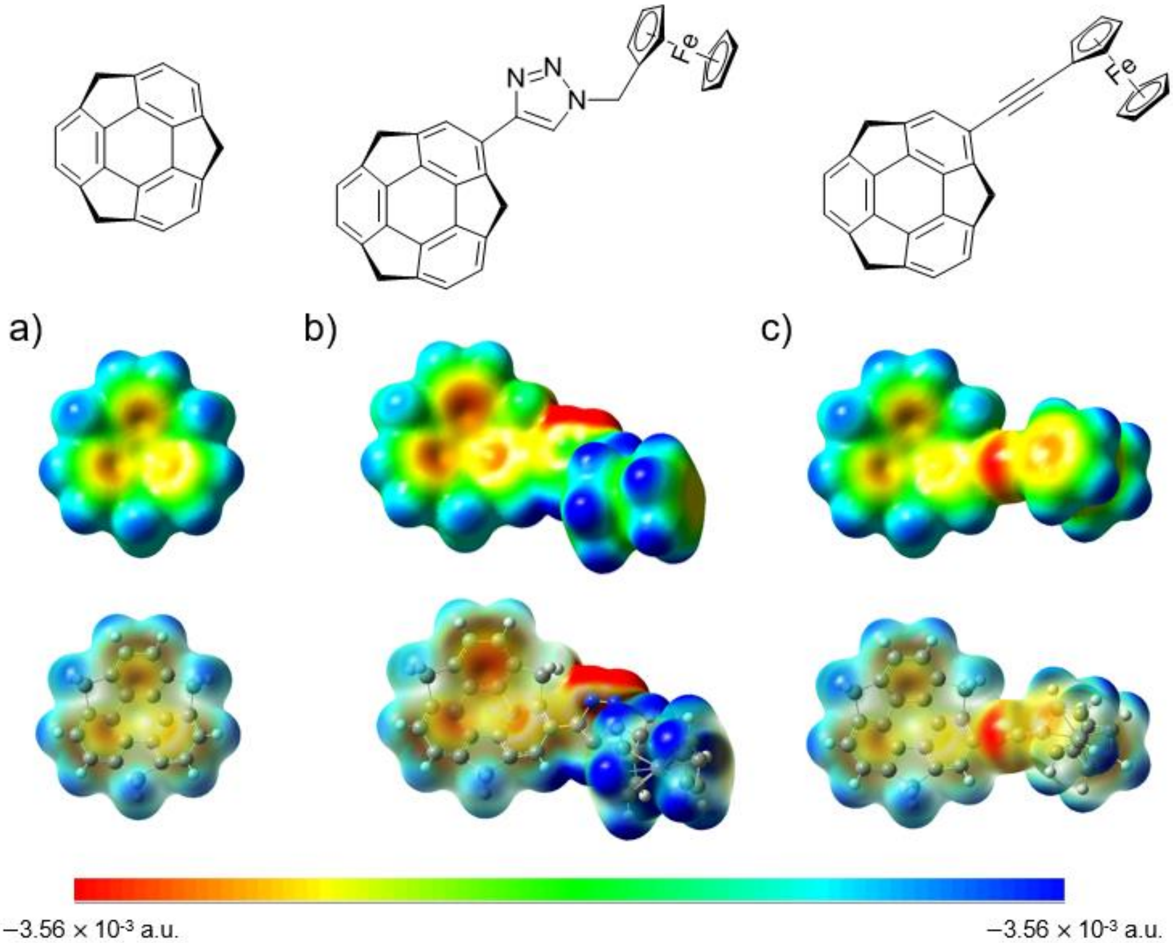

Figure S36. Calculated electrostatic potential (ESP) of a) **1**, b) **7**, and c) **8** from concave face of sumanene bowl**.** Isosurface for electrostatic potential was 0.002 au. The calculation was performed at the ωB97X-D/Def2-SVP level.

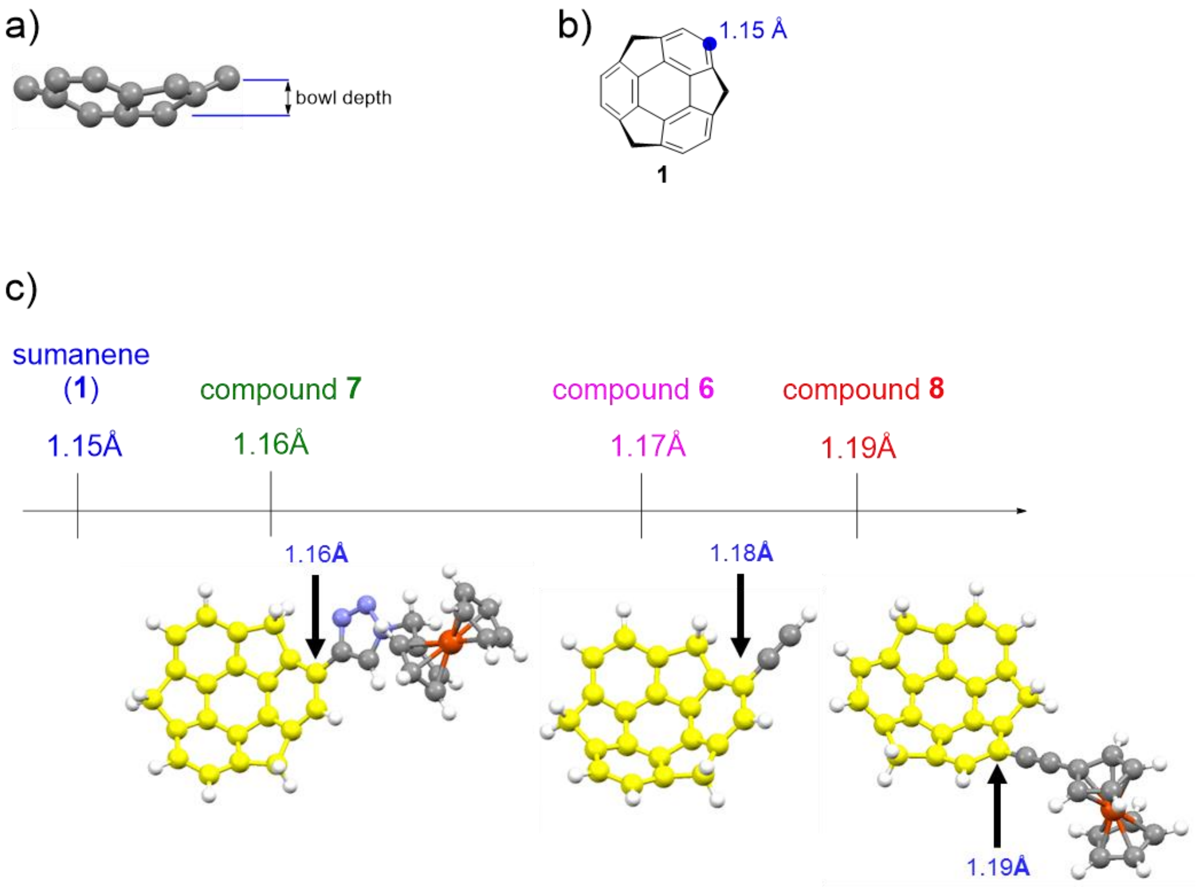

Figure S37. (**a**) Method of defining the bowl depth. (**b**) Bowl depth for sumanene (**1**). (**c**) Structures of sumanene derivatives **6**, **7** and **8** together with their DFT optimized structures (the sumanene bowl is marked yellow) and bowl depths (black arrow denotes the carbon atom for which bowl depth was taken) at the ωB97X-D/Def2-SVP level of theory. The bowl depth for sumanene (**1**) was estimated based on the DFT-optimized structure.

5. Spectrofluorimetric analyzes of the interactions between monoferrocenylsumanene 7 or 8 and cesium cations

To provide the solubility of **7** and **8** and cesium salt, the measurements were carried out in methanol-chloroform mixture (1:1 *v*/*v*). Appropriate volumes of $1{\cdot}10^{-4}$ M CsCl solution were mixed with $1{\cdot}10^{-4}$ M solution of monoferrocenylsumanene (**7** or **8**) to reach given sumanene-to-metal cation molar ratio. The concentration of monoferrocenylsumanene in each sample was $2{\cdot}10^{-5}$ M. The excitation wavelength was 285 nm.

Complex stoichiometries were estimated with the Job's plot method (continuous variation method).[16–19]

The apparent binding constants (K_{app}) were estimated with the Benesi-Hildebrand method[20,21], using the following equation:

$$\frac{1}{I - I_0} = \frac{1}{a} + \frac{1}{a \cdot K_{app} \cdot C(\mathrm{Cs}^+)}$$

where I_0 and I are the fluorescence intensities of sumanene-ferrocene conjugate in the absence and presence of cesium cations, respectively, a is a constant, and $C(\mathrm{Cs}^+)$ is the concentration of cesium cations in solution. The association constant was determined as a ratio of intercept-to-slope of $1/(I - I_0)$ vs. $1/C(\mathrm{Cs}^+)$ linear plot.

The above-discussed spectra and data are presented below.

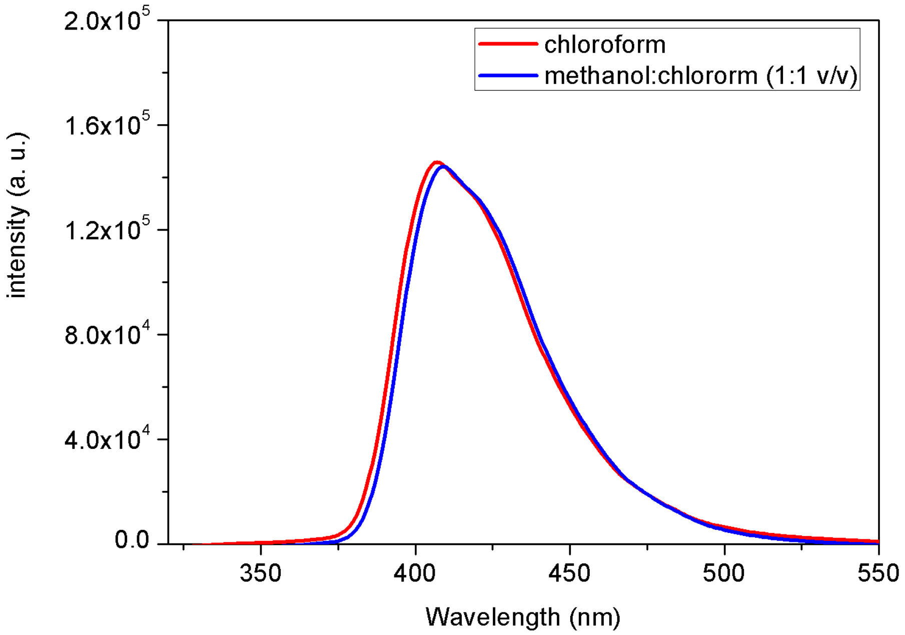

Figure S38. Comparison between emission spectra (λ_{ex} = 285 nm) of **8** in $CHCl_3$ and $CHCl_3$:CH_3OH (1/1 *v*/*v*).

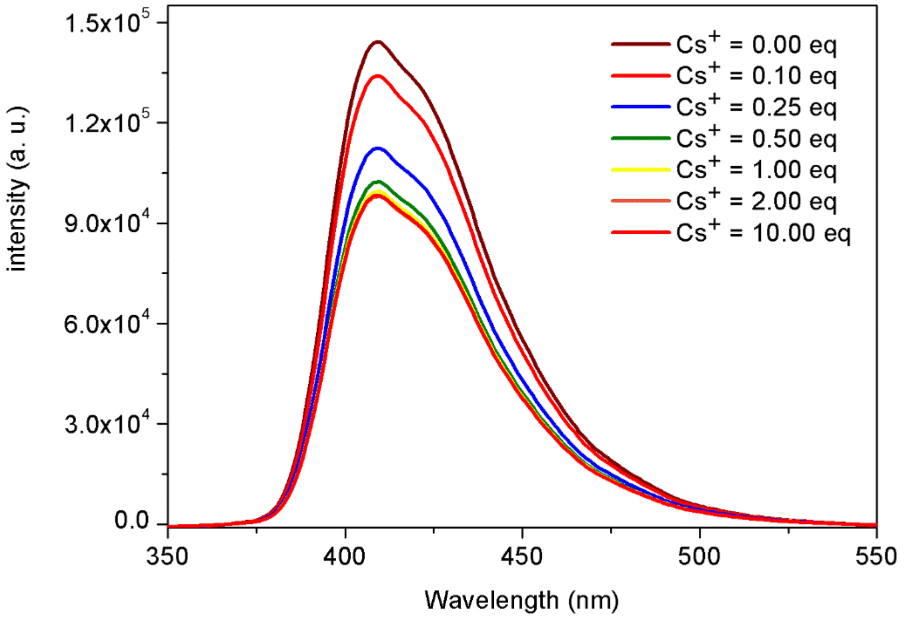

Figure S39. Emission spectra (λ_{ex} = 285 nm) of **8** in the presence of various amounts (equivalents = eq) of cesium cations.

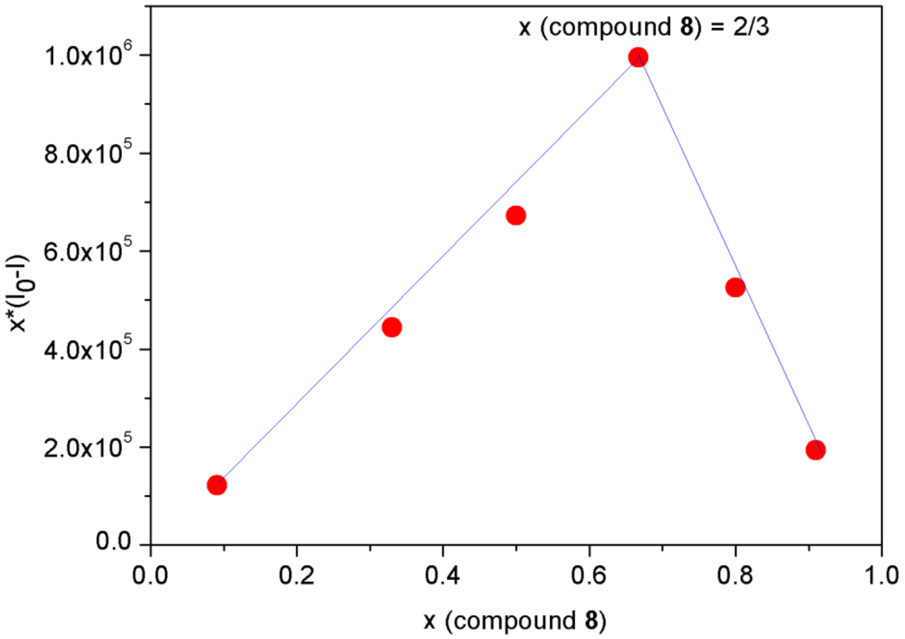

Figure S40. Job's plot regarding the interactions between monoferrocenylsumanene **8** and cesium cations (*x* stands for the molar fraction of **8**; I_0 and I are the fluorescence intensities of **8** in the absence and in the presence of cesium cations).

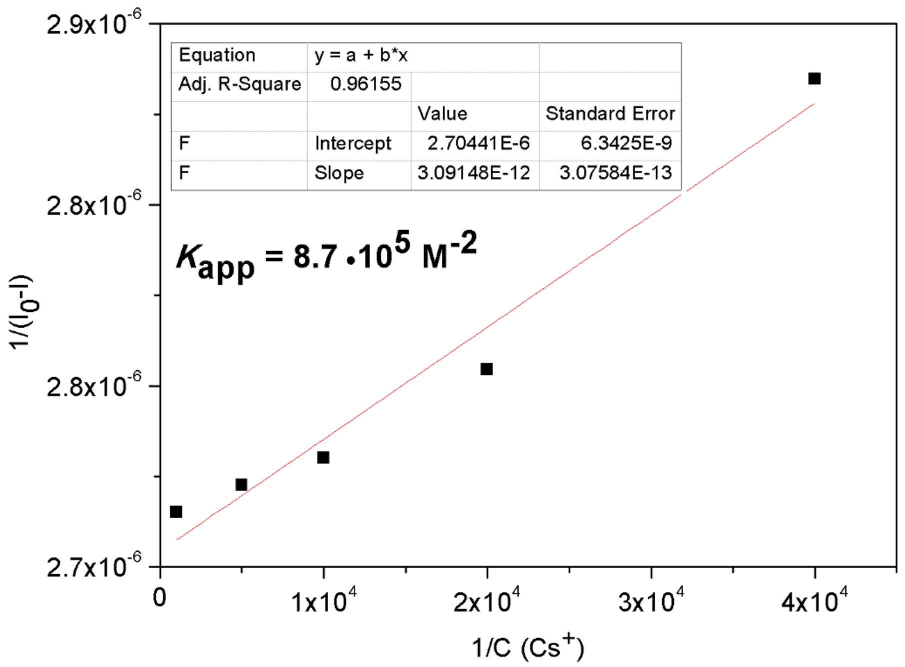

Figure S41. Benesi-Hildebrand plot regarding the interactions between **8** and cesium cations (C stands for the molar concentration of cesium cations in the sample; I_0 and I are the fluorescence intensities of **8** in the absence and in the presence of cesium cations). The linear fit data and the calculated K_{app} are also presented.

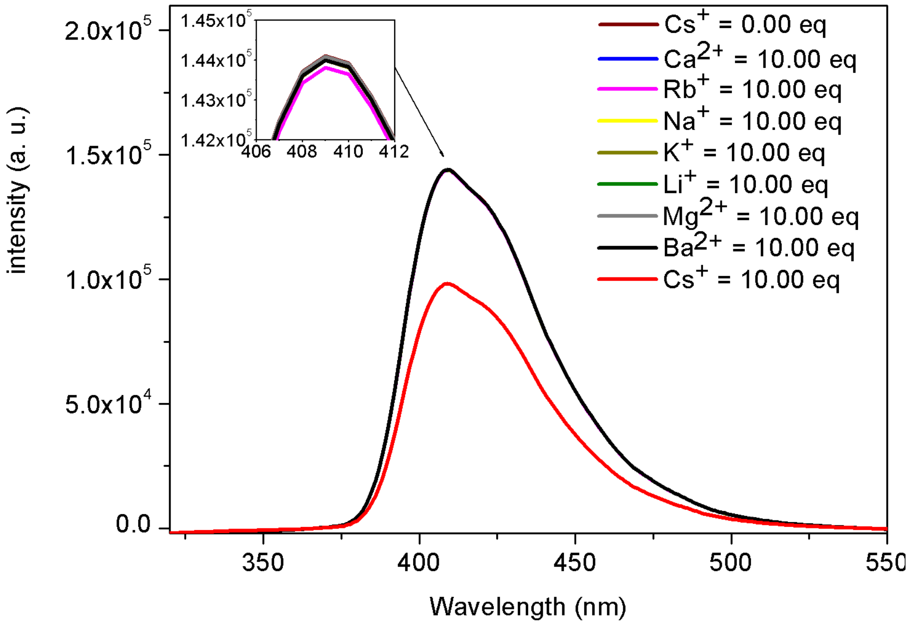

Figure S42. Emission spectra (λ_{ex} = 285 nm) of **8** in the absence or in the presence of various cations (10 eq).

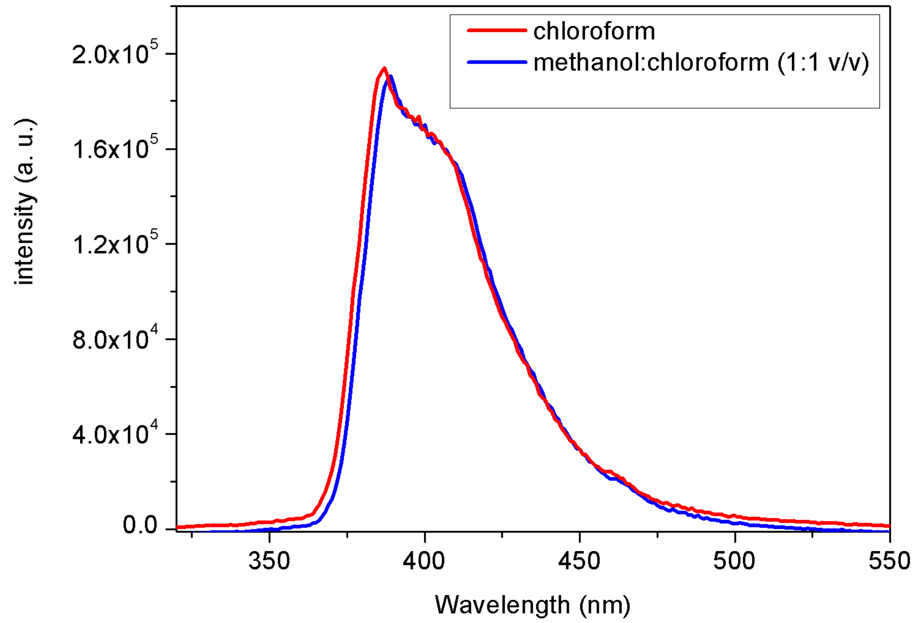

Figure S43. Comparison between emission spectra (λ_{ex} = 285 nm) of **7** in $CHCl_3$ and $CHCl_3$:CH_3OH (1/1 *v*/*v*).

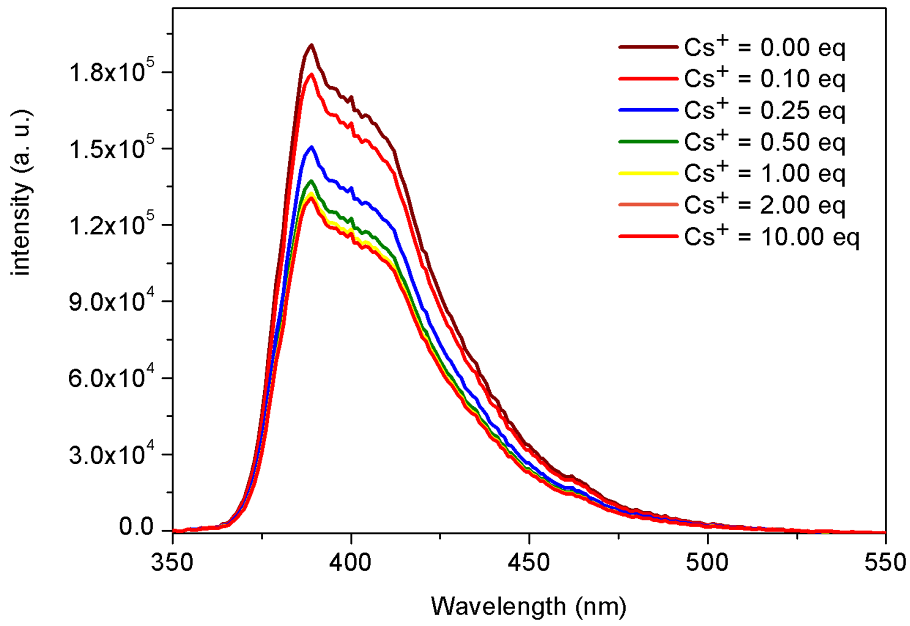

Figure S44. Emission spectra (λ_{ex} = 285 nm) of **7** in the presence of various amounts (equivalents = eq) of cesium cations.

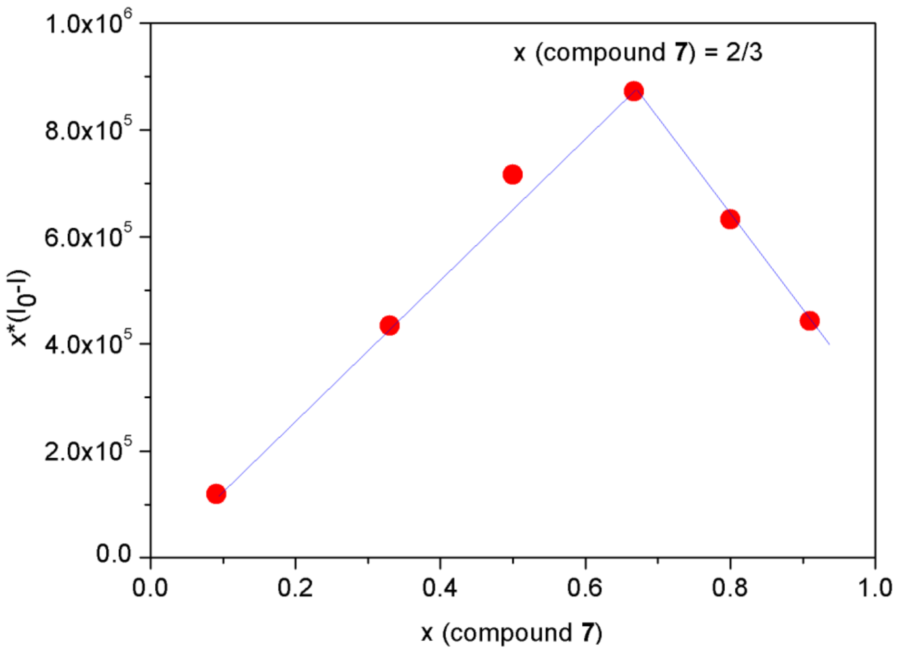

Figure S45. Job's plot regarding the interactions between **7** and cesium cations (*x* stands for the molar fraction of **7**; I_0 and I are the fluorescence intensities of **7** in the absence and in the presence of cesium cations).

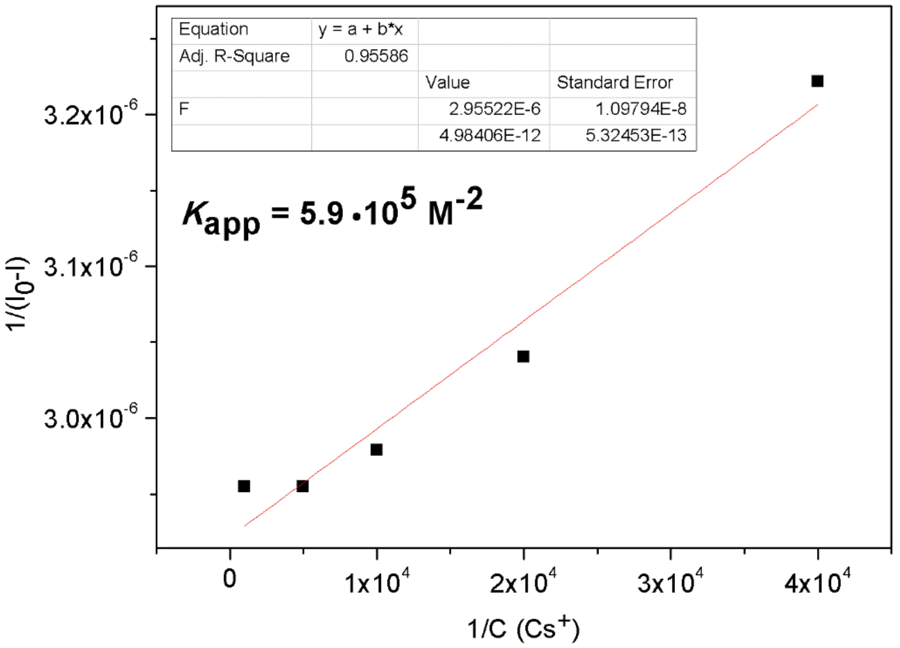

Figure S46. Benesi-Hildebrand plot regarding the interactions between monoferrocenylsumanene **7** and cesium cations (*C* stands for the molar concentration of cesium cations in the sample; I_0 and I are the fluorescence intensities of **7** in the absence and in the presence of cesium cations). The linear fit data and the calculated K_{app} are also present.

6. Electrochemical characterization of monoferrocenylsumanenes 7 and 8

Cyclic voltammetry (CV) experiments were performed to electrochemically characterize the synthesized monoferrocenylsumanenes **7** and **8**. The voltammograms were recorded in DCM at different scan rates, ranging from 0.002 to 1 $V \cdot s^{-1}$. The typical cycling voltammograms of **7** and **8** are presented in **Figure S47**. The voltammograms of both compounds are characterized by one pair of current signals (anodic and cathodic) corresponding to the $Fe^{2+/3+}$ redox couple in the ferrocene unit.[22–24] In the case of fast, reversible and one-electron process ,the peak potential separation ($\Delta E_p = E_{pa} - E_{pc}$) should equal to 0.059 V (298 K) and the peak current ratio (I_{pa}/I_{pc}) should equal to 1.[25] Considering the electrochemical parameters of the studied ferrocene derivatives **7** and **8** (refer to **Table S3**), it can be concluded that the conjugation of ferrocene unit with sumanene through acetylene or 1,2,3-triazole does not significantly affect the electrode process. The dependencies of anodic peak heights in the function of the square root of the scan rate are shown in the insets in **Figure S47**. In the whole studied scan rate range, the relationship of I_{pa} *versus* $(v)^{0.5}$ was linear, what indicates clear diffusion character of the electrode process. From the slope of the plot $I_{pa} = f(v)^{0.5}$ the diffusion coefficients of the studied ferrocene derivatives were calculated. The obtained values (**Table S3**) are very similar to the value for unmodified ferrocene. The $\ln(I_{pa}) = f(E_{pa}\text{-}E_f)$ dependencies were plotted to get the information about the influence of the type of linker on the electron-transfer rate constant (k_0). The value of the electron-transfer rate constant was determined from the slope of the curve, according to the formula:

$$I_{pa}=0.227nFAC_0^{*}k_0 \exp\left[-\frac{\alpha nF}{RT}(E_{pa}-\mathrm{E_f})\right] \qquad (1)$$

where: I_{pa} is a current intensity of the anodic peak, n – number of electron exchange during electrode process, F is Faraday constant, A – electrode surface area, C_0^{*} concentration of the electroactive species, E_{pa} – potential of the anodic peak, E_f – formal potential, R – gas constant, T – temperature and α is transition coefficient. Similarly, as in the case of the diffusion coefficient values, no significant differences were found compared to the standard (unmodified ferrocene).

The results of selectivity studies with cyclic voltammetry are presented in **Figure S46**.

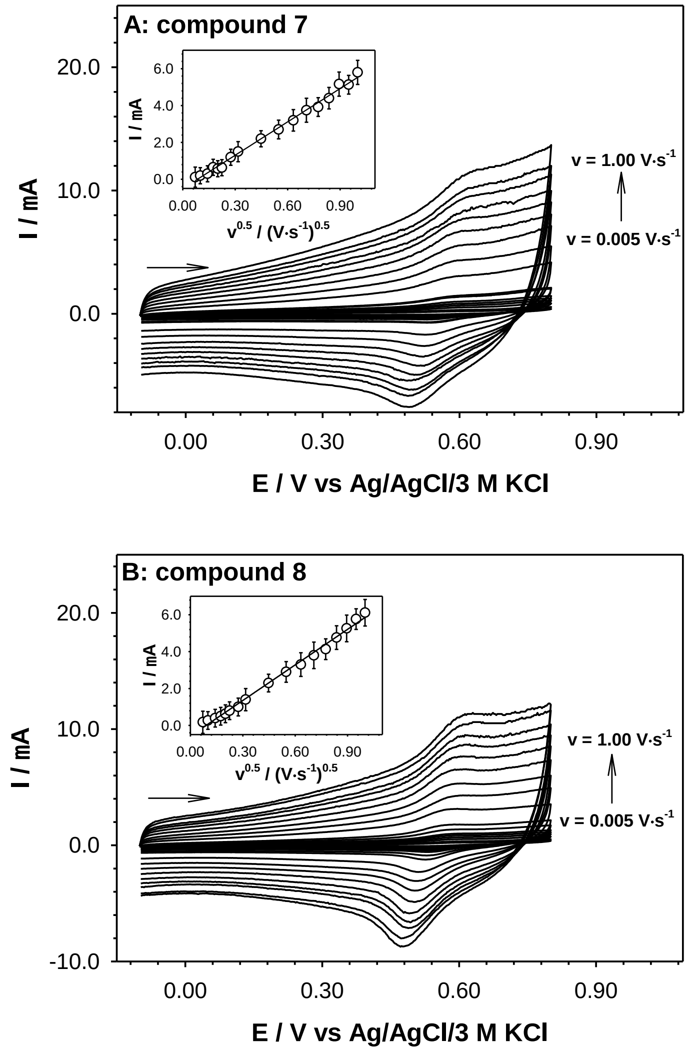

Figure S47. Cyclic voltammograms (plotted in IUPAC convention) of monoferrocenylsumanenes **7** (A) and monoferrocenylsumanene **8** (B) recorded in DCM. Insets: Dependencies of anodic peak currents *vs.* square root of scan rate. Experimental conditions: solvent: dichloromethane (DCM), supporting electrolyte: tetrabutylammonium hexafluorophosphate ($TBAPF_6$), $C_{compound}$ = 0.02 mM, C_{TBAPF6} = 100 mM, T = 21 °C, working electrode: disc glassy electrode (ϕ = 3 mm), counter electrode: Pt plate (A = *ca.* 1 cm^2, reference electrode: Ag/AgCl/3 M KCl.

Table S3. The NMR signal assignments Electrochemical parameters (I_{pa}: current intensity of the anodic peak, E_{pa}: potential of the anodic peak, I_{pc}: current intensity of the cathodic peak, E_{pc}: potential of the cathodic peak, E_f: formal potential, D: diffusion coefficient, k_0: electron-transfer rate constant) of monoferrocenylsumanenes **7** and **8** estimated from CV voltammograms recorded at scan rate equal 0.1 V·s^{-1} in DCM.

Compound	I_{pa} [μA]	E_{pa} [V]	I_{pc} [μA]	E_{pc} [V]	I_{pa}/I_{pc}	ΔE_p [V]	E_f [V]	$D_S \cdot 10^5$ [cm^2·s^{-1}]	$k_0 \cdot 10^3$ [cm·s^{-1}]
**7**	1.5 ± 0.2	0.608	-(1.4 ± 0.3)	0.527	1.07	0.081	0.568	1.22	1.35
**8**	1.4 ± 0.1	0.598	-(1.3 ± 0.1)	0.527	1.11	0.071	0.563	1.86	3.95
**ferrocene**	1.9 ± 0.2	0.448	-(2.0 ± 0.1)	0.376	0.95	0.072	0.412	1.67[22]	2.83

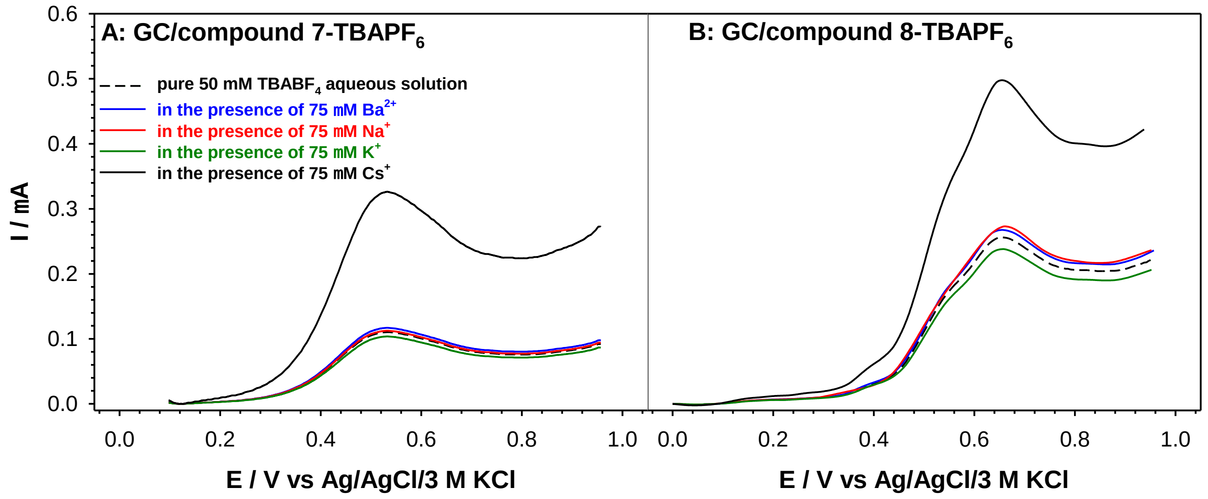

Figure S48. Cyclic voltammograms (plotted in IUPAC convention) of monoferrocenylsumanenes **7** (A) and monoferrocenylsumanene **8** (B) recorded in DCM. Insets: Dependencies of anodic peak currents *vs.* square root of scan rate. Experimental conditions: solvent: dichloromethane (DCM), supporting electrolyte: tetrabutylammonium hexafluorophosphate ($TBAPF_6$), $C_{compound}$ = 0.02 mM, C_{TBAPF6} = 100 mM, T = 21 °C, working electrode: disc glassy electrode (ϕ = 3 mm), counter electrode: Pt plate (A = *ca.* 1 cm^2, reference electrode: Ag/AgCl/3 M KCl.

7. Additional data on LA-ICP-MS

Elemental distribution of Fe, Cs, Ba, Na and K baed on LA-ICP-MS analyzes at the surface of the samples labelled as compound **7** and compound **8** are presented in **Table S4**.

Table S4. Elemental distribution of Fe, Cs, Ba, Na and K at the surface of the samples labelled as compound **7** and compound **8**.

	Fe	Cs	Ba	Na	K
Compound 7	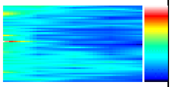	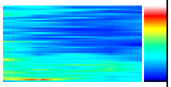	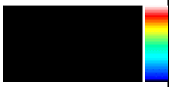	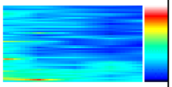	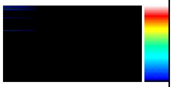
Compound 8	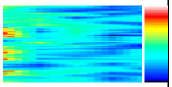	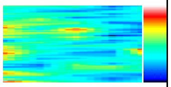	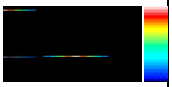	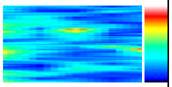	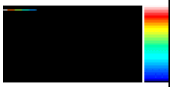

8. Supporting references

(1) Sakurai, H.; Daiko, T.; Hirao, T. A Synthesis of Sumanene, a Fullerene Fragment. *Science* **2003**, *301* (5641), 1878–1878. https://doi.org/10.1126/science.1088290.

(2) Amaya, T.; Seki, S.; Moriuchi, T.; Nakamoto, K.; Nakata, T.; Sakane, H.; Saeki, A.; Tagawa, S.; Hirao, T. Anisotropic Electron Transport Properties in Sumanene Crystal. *J. Am. Chem. Soc.* **2009**, *131* (2), 408–409. https://doi.org/10.1021/ja805997v.

(3) Shrestha, B. B.; Higashibayashi, S.; Sakurai, H. Columnar/Herringbone Dual Crystal Packing of Pyrenylsumanene and Its Photophysical Properties. *Beilstein J. Org. Chem.* **2014**, *10*, 841–847. https://doi.org/10.3762/bjoc.10.80.

(4) Shrestha, B. B.; Karanjit, S.; Panda, G.; Higashibayashi, S.; Sakurai, H. Synthesis of Substituted Sumanenes by Aromatic Electrophilic Substitution Reactions. *Chem. Lett.* **2013**, *42* (4), 386–388. https://doi.org/10.1246/cl.121273.

(5) Casas-Solvas, J. M.; Ortiz-Salmerón, E.; Giménez-Martínez, J. J.; García-Fuentes, L.; Capitán-Vallvey, L. F.; Santoyo-González, F.; Vargas-Berenguel, A. Ferrocene-Carbohydrate Conjugates as Electrochemical Probes for Molecular Recognition Studies. *Chem. Eur. J.* **2009**, *15* (3), 710–725. https://doi.org/10.1002/chem.200800927.

(6) Wang, Y.; McGonigal, P. R.; Herlé, B.; Besora, M.; Echavarren, A. M. Gold(I) Carbenes by Retro-Buchner Reaction: Generation and Fate. *J. Am. Chem. Soc.* **2014**, *136* (2), 801–809. https://doi.org/10.1021/ja411626v.

(7) Zhu, D.; Peng, H.; Sun, Y.; Wu, Z.; Wang, Y.; Luo, B.; Yu, T.; Hu, Y.; Huang, P.; Wen, S. Modular Metal-Free Catalytic Radical Annulation of Cyclic Diaryliodoniums to Access π-Extended Arenes. *Green Chem.* **2021**, *23* (5), 1972–1977. https://doi.org/10.1039/D0GC04183A.

(8) Vasilopoulos, A.; Zultanski, S. L.; Stahl, S. S. Feedstocks to Pharmacophores: Cu-Catalyzed Oxidative Arylation of Inexpensive Alkylarenes Enabling Direct Access to Diarylalkanes. *J. Am. Chem. Soc.* **2017**, *139* (23), 7705–7708. https://doi.org/10.1021/jacs.7b03387.

(9) Abraham, R. J.; Warne, M. A.; Griffiths, L. Proton Chemical Shifts in NMR. Part 10.1 Bromine and Iodine Substituent Chemical Shifts (SCS) and an Analysis of the Contributions to the SCS in Halocyclohexanes. *J. Chem. Soc., Perkin Trans. 2* **1997**, No. 11, 2151–2160. https://doi.org/10.1039/a704537i.

(10) Butler, R. N. A Study of the Proton Nuclear Magnetic Resonance Spectra of Aryl and Mono- and Disubstituted *N* -Methylazoles. *Can. J. Chem.* **1973**, *51* (14), 2315–2322. https://doi.org/10.1139/v73-346.

(11) Frisch, M. J.; Trucks, G. W.; Schlegel, H. B.; Scuseria, G. E.; Robb, M. A.; Cheeseman, J. R.; Scalmani, G.; Barone, V.; Petersson, G. A.; Nakatsuji, H.; Li, X.; Caricato, M.; Marenich, A. V.; Bloino, J.; Janesko, B. G.; Gomperts, R.; Mennucci, B.; Hratchian, H. P.; Ortiz, J. V.; Izmaylov, A. F.; Sonnenberg, L.; Williams-Young, D.; Ding, F.; Lipparini, F.; Egidi, F.; Goings, J.; Peng, B.; Petrone, A.; Henderson, T.; Ranasinghe, D.; Zakrzewski, V. G.; Gao, J.; Rega, N.; Zheng, G.; Liang, W.; Hada, M.; Ehara, M.; Toyota, K.; Fukuda, R.; Hasegawa, J.; Ishida, M.; Nakajima, T.; Honda, Y.; Kitao, O.; Nakai, H.; Vreven, T.; Throssell, K.; Montgomery, Jr., J. A.; Peralta, J. E.; Ogliaro, F.; Bearpark, M. J.; Heyd, J. J.; Brothers, E. N.; Kudin, K. N.; Staroverov, V. N.; Keith, T. A.; Kobayashi, R.; Normand, J.; Raghavachari, K.; Rendell, A. P.; Burant, J. C.; Iyengar, S. S.; Tomasi, J.; Cossi, M.; Millam, J. M.; Klene, M.; Adamo, C.; Cammi, R.; Ochterski, J. W.; Martin, R. L.; Morokuma, K.; Farkas, O.; Foresman, J. B.; Fox, D. J. 2016.

(12) Chai, J.-D.; Head-Gordon, M. Long-Range Corrected Hybrid Density Functionals with Damped Atom–Atom Dispersion Corrections. *Phys. Chem. Chem. Phys.* **2008**, *10* (44), 6615. https://doi.org/10.1039/b810189b.

(13) Weigend, F.; Ahlrichs, R. Balanced Basis Sets of Split Valence, Triple Zeta Valence and Quadruple Zeta Valence Quality for H to Rn: Design and Assessment of Accuracy. *Phys. Chem. Chem. Phys.* **2005**, *7* (18), 3297. https://doi.org/10.1039/b508541a.

(14) Yakiyama, Y.; Hasegawa, T.; Sakurai, H. Formation of a Large Confined Spherical Space with a Small Aperture Using Flexible Hexasubstituted Sumanene. *J. Am. Chem. Soc.* **2019**, *141* (45), 18099–18103. https://doi.org/10.1021/jacs.9b07902.
(15) Amaya, T.; Nakata, T.; Hirao, T. Synthesis of Highly Strained π-Bowls from Sumanene. *J. Am. Chem. Soc.* **2009**, *131* (31), 10810–10811. https://doi.org/10.1021/ja9031693.
(16) Renny, J. S.; Tomasevich, L. L.; Tallmadge, E. H.; Collum, D. B. Method of Continuous Variations: Applications of Job Plots to the Study of Molecular Associations in Organometallic Chemistry. *Angew. Chem. Int. Ed.* **2013**, *52* (46), 11998–12013. https://doi.org/10.1002/anie.201304157.
(17) Kasprzak, A.; Kowalczyk, A.; Jagielska, A.; Wagner, B.; Nowicka, A. M.; Sakurai, H. Tris(Ferrocenylmethidene)Sumanene: Synthesis, Photophysical Properties and Applications for Efficient Caesium Cation Recognition in Water. *Dalton Trans.* **2020**, *49* (29), 9965–9971. https://doi.org/10.1039/D0DT01506G.
(18) Kasprzak, A.; Sakurai, H. Disaggregation of a Sumanene-Containing Fluorescent Probe towards Highly Sensitive and Specific Detection of Caesium Cations. *Chem. Commun.* **2021**, *57* (3), 343–346. https://doi.org/10.1039/D0CC07226E.
(19) Kasprzak, A.; Sakurai, H. Site-Selective Cation–π Interaction as a Way of Selective Recognition of the Caesium Cation Using Sumanene-Functionalized Ferrocenes. *Dalton Trans.* **2019**, *48* (46), 17147–17152. https://doi.org/10.1039/C9DT03162F.
(20) Benesi, H. A.; Hildebrand, J. H. A Spectrophotometric Investigation of the Interaction of Iodine with Aromatic Hydrocarbons. *J. Am. Chem. Soc.* **1949**, *71* (8), 2703–2707. https://doi.org/10.1021/ja01176a030.
(21) Goswami, S.; Aich, K.; Das, S.; Das, A. K.; Manna, A.; Halder, S. A Highly Selective and Sensitive Probe for Colorimetric and Fluorogenic Detection of Cd2+ in Aqueous Media. *Analyst* **2013**, *138* (6), 1903. https://doi.org/10.1039/c3an36884j.
(22) Tsierkezos, N. G. Cyclic Voltammetric Studies of Ferrocene in Nonaqueous Solvents in the Temperature Range from 248.15 to 298.15 K. *J Solution Chem* **2007**, *36* (3), 289–302. https://doi.org/10.1007/s10953-006-9119-9.
(23) Lewandowski, A.; Waligora, L.; Galinski, M. Ferrocene as a Reference Redox Couple for Aprotic Ionic Liquids. *Electroanalysis* **2009**, *21* (20), 2221–2227. https://doi.org/10.1002/elan.200904669.
(24) Neghmouche, N.; Khelef, A.; Lanez, T. Electrochemistry Characterization of Ferrocene/Ferricenium Redox Couple at Glassycarbon Electrode. *J. Fundam and Appl Sci.* **2015**, *1* (2), 23. https://doi.org/10.4314/jfas.v1i2.3.
(25) Bard, A. J.; Faulkner, L. R. *Electrochemical Methods: Fundamentals and Applications*, 2nd ed.; Wiley: New York, 2001.
